# Photocatalytic cyclization of nitrogen-centered radicals with carbon nitride through promoting substrate/catalyst interaction

**DOI:** 10.1038/s41467-022-32623-3

**Published:** 2022-08-20

**Authors:** Mingcheng Yang, Ronghong Lian, Xirui Zhang, Chong Wang, Jiajia Cheng, Xinchen Wang

**Affiliations:** 1 State Key Laboratory of Photocatalysis on Energy and Environment, College of Chemistry, Fuzhou University, Fuzhou, 350116 China; 2 Qingyuan Innovation Laboratory, Quanzhou, 362801 China

**Keywords:** Catalyst synthesis, Synthetic chemistry methodology, Photocatalysis

## Abstract

The use of metal-free carbon nitride and light to drive catalytic transformations constitutes a sustainable strategy for organic synthesis. At the moment, enhancing the intrinsic activity of CN catalysts by tuning the interfacial coupling between catalyst and substrate remains challenging. Herein, we demonstrate that urea-derived carbon nitride catalysts with the abundant −NH_2_ groups and the relative positive charged surface could effectively complex with the deprotonated anionic intermediate to improve the adsorption of organic reactants on the catalyst surface. The decreased oxidation potential and upshift in its highest occupied molecular orbital position make the electron abstraction kinetics by the catalyst more energetically favorable. The prepared catalyst is thus utilized for the photocatalytic cyclization of nitrogen-centered radicals for the synthesis of diverse pharmaceutical-related compounds (33 examples) with high activity and reusability, which shows competent performance to the homogeneous catalysts.

## Abstract (web summary)

Carbon nitride catalysts with positively charged surfaces and abundant −NH2 are found to be effective photocatalysts for dihydropyrazole synthesis. A surface-mediated mechanism where deprotonated intermediates interact with the surface is proposed.

## Introduction

Five-membered heterocyclics, such as the dihydropyrazole moiety as well as their unsaturated counterpart pyrazoles, are extensively found in pharmacologically active molecules, agrochemicals, natural products, and synthetic ligands^1,2^. The synthetic approaches towards the aza-heterocycles through photocatalytic intramolecular addition of nitrogen-centered radicals hold several advantages^3–5^. Nonetheless, due to the high bond dissociation free energies (BDFEs) of most N-H bonds (typically > 100 kcal/mol)^6^, giving rise to the nitrogen-centered radicals directly from N-H bonds meets several challenges^7,8^. Irradiating by the proton-coupled electron transfer (PCET) in the natural photosynthesis process^9^, an oxidative deprotonation electron transfer strategy combining base and photocatalyst has been developed^10,11^. Acridinium salts, as well as transition metal complexes based on ruthenium and iridium, have been shown to be good photocatalysts for preparing the dihydropyrazole frameworks^12–15^. Regardless of the advances made in the field of homogeneous catalysts, many issues remain regarding costs, catalyst recovery, stability, and particularly metal leaching^16–18^. As a result, the development of efficient and selective strategies for metal-free heterogeneous photocatalytic heterocycle synthesis is still highly desirable.

Tailoring the interfacial interaction between semiconductor photocatalyst and the substrate is considered an important approach in the pursuit of advanced heterogeneous catalysis. The surface properties of the heterogeneous catalyst are crucial because they affect both the substrate-catalyst interaction and the mobility of the charge carriers^19–21^. The use of surface properties in heterogeneous catalysts also offers considerable opportunities to facilitate selective organic reactions through modulation of the reaction kinetics. Several important industrial transformations are catalyzed or promoted via the corporation of acid and base surface sites of catalysts, such as oil cracking, alkylation, and isomerization^22,23^. The electron density of the metal nanoparticle and consequently the adsorption of organic molecules on their surface could also be effectively modified through the interaction between metal catalyst and support. For instance, adjustment of the catalyst surface basicity may improve the photocatalytic performance in the oxidation of the primary alcohols to aldehydes^24^, while the effective promotion of hydrogen transfer by basic sites on bismuth oxybromide can enhance solar-driven reduction and oxidation reactions^25^.

Benefitting from the high thermal and chemical stability, superior photovoltaic characteristics, and unique electronic structure, polymeric carbon nitride (CN) materials have been demonstrated to be efficient photocatalysts for a variety of catalytic reactions^26–28^. The CN materials possess distinctive surface properties such as functional Brønsted acid and base sites^29–31^, which can endow the system with a catalytic activity that is absent in the pristine materials^32,33^. However, most of the current CN-photocatalysts for selective organic synthesis have concentrated on thermodynamic processes. Enhancing the intrinsic activity by tuning the interfacial interaction between catalyst and substrate remains challenging^34–36^. As a result, we propose that the redox potentials of critical organic reactants, specifically the deprotonated anionic intermediate in this case, can be altered by surface complexation with the CN catalyst, which can then be used in conjunction with photocatalyst to enable demanding conversions under mild conditions.

**Fig. 1:**
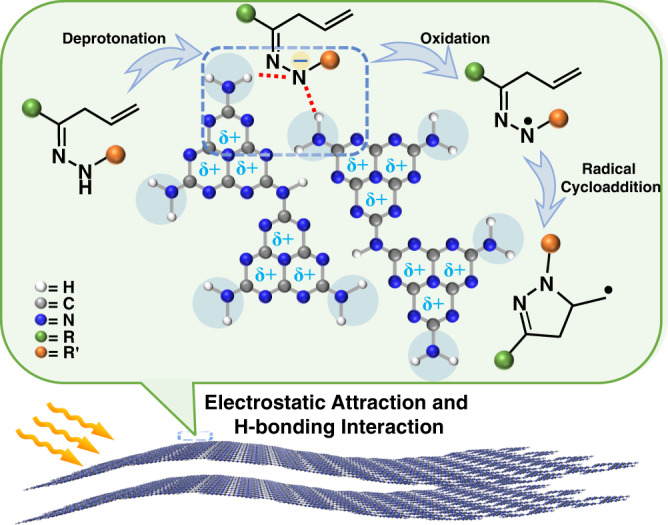
Interface assisted catalytic cyclization of nitrogen-centered radicals over carbon nitride. Photocatalytic oxidative cyclization of hydrazones through promoting substrate/catalyst interaction.

We report herein the general heterogeneous photocatalytic strategy for the synthesis of functionalized dihydropyrazoles from N-H hydrazone^37^ over CN (Fig. 1). Three CN samples (g-CN-U, g-CN-DCDA, and K-PHI) with different surface properties were prepared^38,39^. It is observed that urea-derived carbon nitride (g-CN-U) with abundant −NH_2_ groups and relative positive surface could effectively complex with the in-situ generated anionic intermediate to improve the adsorption and activation of the key species on the catalyst surface. Moreover, the upshift in its highest occupied molecular orbital (HOMO) position makes the electron abstraction more energetically favorable. Therefore, the highly selective oxidation of the hydrazone N-H bond to nitrogen-centered radical could be achieved over g-CN-U, whereas g-CN-DCDA and K-PHI showcased inferior activity^40^. This approach would open up the possibility of utilizing surface complexation between the catalysts and vital reaction intermediates to promote a broad range of photoredox reactions.

## Results and discussion

### Photocatalytic cyclization of nitrogen-centered radicals over carbon nitride

**Fig. 2:**
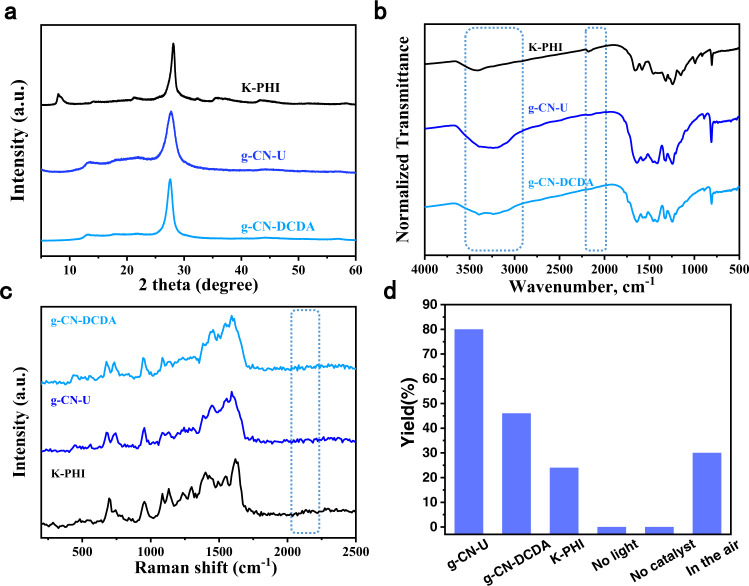
Structural characterizations. **a** PXRD result of g-CN-U, g-CN-DCDA, K-PHI. **b** FT-IR spectra of g-CN-U, g-CN-DCDA, K-PHI. **c** Raman spectra of g-CN-U, g-CN-DCDA, K-PHI. **d** Photocatalytic activities under the different photocatalysts: 1a (0.1 mmol), NaOH (1.5 equiv.), catalyst (5 mg), CHCl_3_ (2 mL), 3 W 420 nm LED, under N_2_ at room temperature for 8 h. Yields determined by ^1^H NMR using benzyl ether as an internal standard.

For comparison, three CN samples with distinct surface properties were prepared. The g-CN-U photocatalyst, which possesses a positive surface^38^, is synthesized by pyrolysis of urea at 550 ^o^C. The g-CN-DCDA sample with fewer −NH_2_ groups and different surface properties is synthesized from dicyandiamide (DCDA). The K-PHI catalyst, which is derived from preheated melamine after KCl/LiCl molten salt treatment is chosen as another comparative photocatalyst owing to its negatively charged nitrogen atoms^41,42^. Powder X-ray diffraction (PXRD) analysis confirms the crystalline structure and layered-stacking mode of the photocatalysts (Fig. 2a). The solid-state ^13^C NMR (CP-MAS) of the samples all exhibited two intensive peaks at 160 and 168 ppm, respectively, associated with the chemical shifts of the C(i) and C(e) atoms in the heptazine units. This result suggested a similar heptazine-based structure of the prepared samples (Supplementary Fig. 1). In the Fourier transform infrared (FT-IR) spectroscopy, the characteristic peaks associated with the heptazine units could be observed, suggesting the identical framework of the samples. Notably, the intensity of the peak located at around 3250 cm^−1^ of g-CN-U is stronger than g-CN-DCDA and K-PHI, indicating the existence of abundant NH_2_ groups on the g-CN-U surface. Whereas, K-PHI shows an additional 1000 cm^−1^ peak corresponding to the N-K bond and a 2180 cm^−1^ peak to the cyanamide groups (Fig. 2b). The observed intensity at 2180 cm^−1^ in the Raman spectra of K-PHI may also correlate with the cyanamide-functionality vibrational frequency (Fig. 2c). Elemental analysis (EA) results showed that the samples all contained C, N, H elements with a similar C/N ratio (Supplementary Table 1), whereas the H element of g-CN-U is the largest, consistent with the richness of NH_2_ groups from the IR results. Inductively coupled plasma mass spectrometry atomic emission spectroscopy (ICP-AES) analysis revealed that except for a small amount of K ions existed in the K-PHI sample, none of the Co, Ni, Cu, Ru, or Pd atoms were present in the g-CN-U, g-CN-DCDA and K-PHI samples (Supplementary Table 2). The robustness of the photocatalysts could be demonstrated by the thermogravimetric analysis (TGA), which would not decompose until 400 ^o^C (Supplementary Fig. 2).

The photocatalytic reaction was commenced with hydrazone **1a** as the model substrate, the synthesized CN samples as the photocatalysts, and NaOH as the base under an N_2_ atmosphere. The cyclization cannot proceed via heating conditions due to the high BDFE of the N-H bonds (Supplementary Fig. 3). No product was obtained without the photocatalyst either (Fig. 2d). Basically, a semiconductor with the valence-band edge lower than the nitrogen-centered anion/radical redox potential may photocatalyze the transformation. However, of all the examined CN catalysts, dihydropyrazole was formed as a major product only over g-CN-U. The major byproduct was β,γ-unsaturated ketone derived from hydrolysis of hydrazone and desulfonation derivative of the dihydropyrazole product (Supplementary Fig. 4). g-CN-DCDA led to a lower yield, whilst the K-PHI catalyst shows the least catalytic efficiency (Fig. 2d). Only a trace amount of product was obtained without base (Supplementary Fig. 5a). Other bases such as Na_2_CO_3_, NaHCO_3_, or Et_3_N also delivered the product, albeit in a lower yield. The optimal reaction system was performed in CHCl_3_ (Supplementary Fig. 5b), indicating the possible involvement of the hydrogen atom transfer (HAT) process^43^. Examination of light wavelength showed that the reaction could proceed in extended light wavelength (Supplementary Fig. 5c). In comparison to other commonly used heterogeneous photocatalysts, such as TiO_2_, CdS, BCN, or ZnIn_2_S_4_, g-CN-U showed the best performance (Supplementary Fig. 5d). Consequently, the desired five-membered heterocycle could be produced in 80% isolated yield over metal-free g-CN-U photocatalyst, which is comparable to that of the homogeneous catalytic systems (Supplementary Table 3).

### Mechanism study

**Fig. 3:**
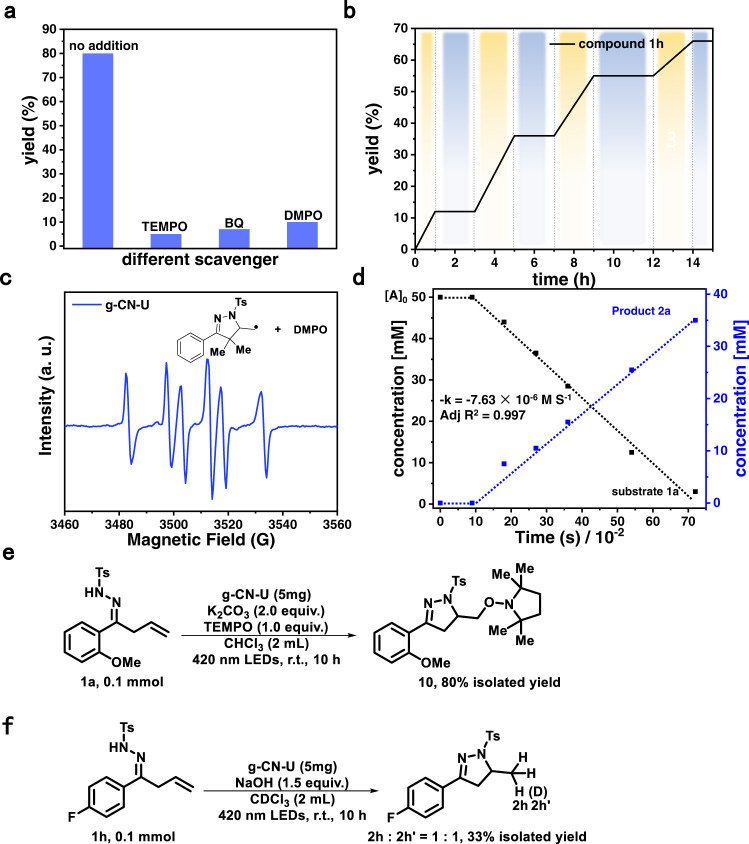
Mechanistic studies. **a** Radical scavenging experiments. **b** Light on-off experiments. **c** ESR spin trapping experiments. **d** Time profile of photocatalytic hydroamination of unsaturated β,γ-hydrazone over g-CN-U. **e** Isolation of the TEMPO-trapping product. **f** Reaction conducted in the deuterated solvent.

As observed from ^1^H NMR, addition of NaOH to the hydrazone solution led to the full deprotonation and the formation of a nitrogen-centered anion swiftly (Supplementary Fig. 6). The oxidation potential of the anion/radical (+0.76 V vs. NHE) by cyclic voltammetry (Supplementary Fig. 7) is substantially less than that of NH/NH^•+^ (+1.0 V vs. NHE) (Supplementary Fig. 8). The inclusion of a radical scavenger (TEMPO, BQ, or DMPO) significantly suppressed the product formation (Fig. 3a). The light on-off switching experiments excluded a radical chain process (Fig. 3b). In situ-electron spin resonance (ESR) spectroscopy revealed a carbon-centered radical species (Fig. 3c). The isolation of TEMPO-trapping product **10** in excellent yield confirmed this hypothesis (Fig. 3e and Supplementary Fig. 9). The reaction was then conducted in CDCl_3_ (Fig. 3f and Supplementary Fig. 10) and a 1:1 ratio of **2** **h** and **2** **h’** in 33% combined yield was observed, confirming the origin of the partial hydrogen source from CHCl_3_. A zero-order kinetic profile indicated that the rate-determining step occurs as a result of adsorption of the reactive species at the g-CN-U surface (Fig. 3d)^44^.

Based on our results and previous reports^11,12^, this reaction possibly proceeds through the following reaction pathway (Supplementary Fig. 11). Deprotonation of hydrazone first occurs to generate the anionic intermediate **Int A**. The single-electron oxidation by the light-excited valence band (VB) hole of the photocatalyst took place to deliver the nitrogen-centered radical **Int B**. A 5-exo-trig cyclization of **Int B** furnished the carbon-centered radical **Int C**. Due to the high instability of the carbon anion (e.g., *t*-butyl radical, E_1/2_^red^ = −2.54 V vs SCE in MeCN)^45^, the reduction of **Int C** to afford the carbon anion may not be feasible. Therefore, HAT from CHCl_3_ (CHCl_3_ exhibited a relatively weak C-H BDE of 93 kJ/mol)^46^ contributed to product **2** and trichloromethyl radical. The formed trichloromethyl radical (Supplementary Fig. 12) could be readily reduced by the conduction band (CB) electron to give trichloromethanide anion (Supplementary Fig. 13) and finally obtained one proton from the environment to complete the catalytic cycle^47^.

### Interface-assisted substrate-catalyst coupling to activate the key deprotonated hydrazone intermediate and promote the reaction

As shown in the UV-vis absorption features (Supplementary Fig. 14), g-CN-U both exhibited one blue-shift and less intensified light absorption with a bandgap of 2.92 eV (Supplementary Fig. 15). The VB maximum of g-CN-U exists at +1.76 V, similar to g-CN-DCDA, while K-PHI exhibit a stronger oxidation capability at +1.90 V (Supplementary Fig. 17). According to the results of photoluminescence (PL) spectrum (Supplementary Fig. 18), photocurrent response and electrochemical impedance spectroscopy (EIS) (Supplementary Fig. 19), the charge generation, separation, and transfer properties of g-CN-U was not optimal. Based on the foregoing findings, it was concluded that the markedly improved activity of g-CN-U did not arise from their differences in light absorption intensity, energy band positions, or charge transfer properties.

**Fig. 4:**
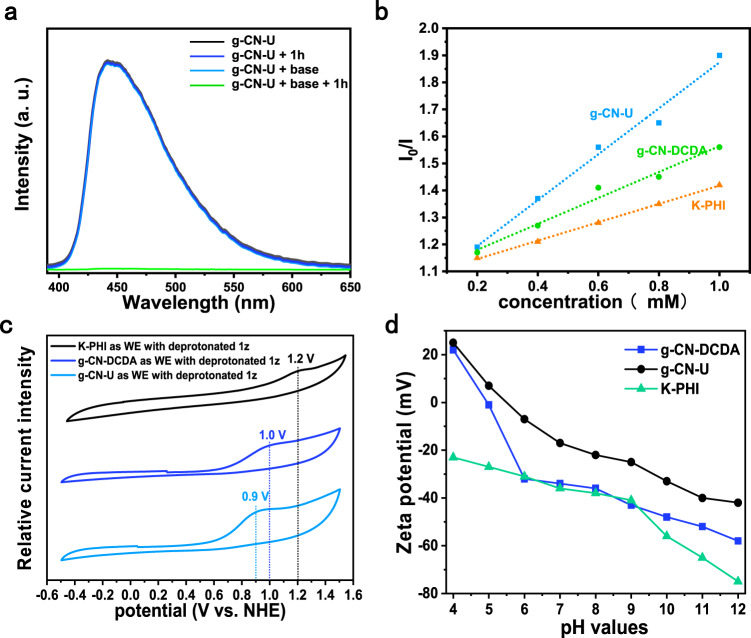
Surface property studies. **a** Steady-state photoluminescence spectra of g-CN-U, g-CN-U/substrate, g-CN-U/base, and g-CN-U/substrate/base in CHCl_3_ (λ_exc_ = 390 nm). **b** Stern-Volmer analysis of the steady-state emission intensity at 450 nm (λ_exc_ = 390 nm) with varied substrate concentration. **c** CVs obtained in a 0.1 M tetrabutylammonium hexafluorophosphate solution containing 2 mM substrate, 2 mM base, and CN as working electrodes (WE). **d** Zeta potential of the samples under various pH values.

The emission quenching spectrum of the suspension was then utilized to probe the photoinduced electron transfer process. As shown in Fig. 4a, the mixture displayed unvaried emission intensity when base or hydrazone was present. However, significant weakening of the emission signal occurred when hydrazone and base were both added. The strengthened interaction between g-CN-U and the deprotonated hydrazone could also be supported by the Stern-Volmer studies. As shown in Fig. 4b and Supplementary Fig. 20, the electron transfer rate between the deprotonated hydrazone and g-CN-U occurred more rapidly than other catalysts. This result could also be supported by the electrochemical study. As shown in Fig. 4c, of all the examined catalysts, g-CN-U showed the lowest oxidation overpotential, suggesting the favored kinetic due to the stronger interaction between g-CN-U and the anion intermediate.

The adsorption and interaction of the deprotonated hydrazone over CN catalyst are carefully investigated. As revealed by the scanning electron microscopy (SEM) results (Supplementary Fig. 21), g-CN-U possessed a platelets structure with lateral dimensions of 20-60 nm, whereas the transmission electron microscope (TEM) image (Supplementary Fig. 22) exhibited one layered carbon nitride structure consisting of thin and transparent nanosheets. The Brunauer-Emmett-Teller (BET) surface area (Supplementary Fig. 23) showed g-CN-U and K-PHI showcased a much higher 56 and 76 m^2^g^−1^ BET surface area than g-CN-DCDA. The Barrett-Joyner-Halenda (BJH) total pore volume of g-CN-DCDA is 0.060 cm^3^/g, whereas g-CN-U and K-PHI possess a much larger 0.240 and 0.204 cm^3^/g pore volume, respectively (Supplementary Table 4). Therefore, the increased surface area of g-CN-U would benefit the mass transfer and substrate adsorption process and hence play a favorable role in the photocatalytic reaction. Zeta-potential measurement showed that K-PHI and g-CN-DCDA generally exhibited more negative potential than g-CN-U in most of the pH ranges (Fig. 4d)^38^, indicating a stronger interaction is more likely to be formed during g-CN-U and the deprotonated hydrazone. The CO_2_-temperature programmed desorption (TPD) showed that the CO_2_ desorption peak of g-CN-U owns a much lower peak intensity (Supplementary Fig. 24). The XPS spectrum of the CN samples before and after KOH treatment (denoted as g-CN-U-b, g-CN-DCDA-b, and K-PHI-b) was compared. The N1*s* and C1*s* peaks of g-CN-U-b exhibited none of the noticeable shifts compared with g-CN-U (Supplementary Fig. 25), showing the less affected surface properties under the basic conditions. In contrast, the C-N=C, N-(C)_3_, and C-N-H peaks of g-CN-DCDA-b and K-PHI-b moved obviously to the lower binding energy, suggesting a higher electron density after base treatment. The relative positive surface of g-CN-U may originate from the abundant NH_2_ groups on the large solid surface area, which could thoroughly expose to the solution and avoid large influence by the environmental conditions^48^. We have performed further DFT calculations to understand the relationship of NH_2_ groups and the relative positive charged surface in the g-CN-U catalyst. g-C_3_N_4_-type carbon nitride structure is used to simulate the structure of carbon nitride without primary and secondary amino groups and melon-type carbon nitride structure is used to simulate the structure of g-CN-U photocatalyst. As shown in Supplementary Fig. 26, the g-C_3_N_4_-type carbon nitride structure exhibits a + 0.1 positive charge distribution in the heptazine ring, whereas the heptazine rings of melon-type g-CN-U catalyst exhibit a much more positive charge distribution compared with that of the g-C_3_N_4_ sample. This result supports that the heptazine rings in g-CN-U exhibit a positive charge distribution possibly due to the presence of rich attached NH_2_ groups^49^.

Interfacial interaction of the key intermediate on the catalyst surface was investigated with ^1^HNMR measurement. The reaction of hydrazone and base led to the disappearance of the N-H signal at around 7.45 ppm (Supplementary Fig. 6). Addition of g-CN-DCDA to the solution led to the movement of the hydrazone proton chemical shift to the higher magnetic field, possibly resulting from the increased electron density between g-CN-DCDA negative charged surface and the nitrogen-centered anion. K-PHI could not remove the N-H proton from the hydrazone, which may originate from the stronger acidity of the NH-CN groups on the K-PHI surface. Conversely, g-CN-U led to a small proton chemical shift movement to the lower field, suggesting a decreased intermediate electron density resulting from the adsorption at the g-CN-U surface. To investigate the key role of the abundant NH_2_ groups and the relative positive charge distribution on the catalyst surface, for reference, H-PHI, of which the potassium cations in K-PHI are replaced by protons, was prepared by treatment of the K-PHI sample with hydrochloric acid (Supplementary Figs. 27 and 28). However, the transformation with H-PHI as the photocatalyst showed no conversion of the reaction at all. Investigation of the surface charge properties indicates that the H-PHI sample does exhibit a more positive zeta-potential at a low pH value (Supplementary Fig. 29). While due to the presence of plentiful NH-CN groups at the H-PHI surface, these acidic functional groups would quickly deprotonate under high pH value, resulting in a faster zeta potential drop rate than that of K-PHI. And the zeta potential of both samples would reach a similar value at about pH 11. This result implies that the abundant amino groups and the relative positive charged surface are both indispensable for promoting the photocatalytic activity of the transformation.

The interfacial substrate-catalyst coupling is also validated through DFT calculations on free hydrazone, deprotonated hydrazone (Supplementary Fig. 11, **Int A**), and deprotonated hydrazone adsorbed on the melon-based structure of g-CN-U (Supplementary Fig. 30)^50^. As shown in Supplementary Fig. 31, the HOMO of the three systems mainly consists of N-*2p* orbitals. The HOMO energy of the free hydrazone molecule was calculated to be −5.76 eV vs. vacuum. The molecular orbital position moved upward to −5.56 eV when the base existed. The DOS diagrams for the adsorption of the deprotonated hydrazone onto the g-CN-U terminal −NH_2_ sites result in a further upward shift to −4.73 eV vs. vacuum. The distance from the nitrogen atom of hydrazone to the hydrogen atom in the −NH_2_ group of g-CN-U is 1.76 Å and the corresponding adsorption energy is −1.95 eV, suggesting the formation of strong hydrogen-bonding and van der Waals interaction (Supplementary Fig. 30). Therefore, the superiority of the g-CN-U photocatalyst could be ascribed to its appropriate energy band position and large specific surface areas. More importantly, attributed to the abundant NH_2_ groups and positively charged surface of g-CN-U, the strong interfacial substrate-catalyst coupling greatly improved the adsorption and activation of the organic molecules. Furthermore, the upward shift HOMO position and decreased oxidation potential make the electron abstraction kinetics by the photocatalyst more energetically favorable.

### Substrate scope

**Fig. 5:**
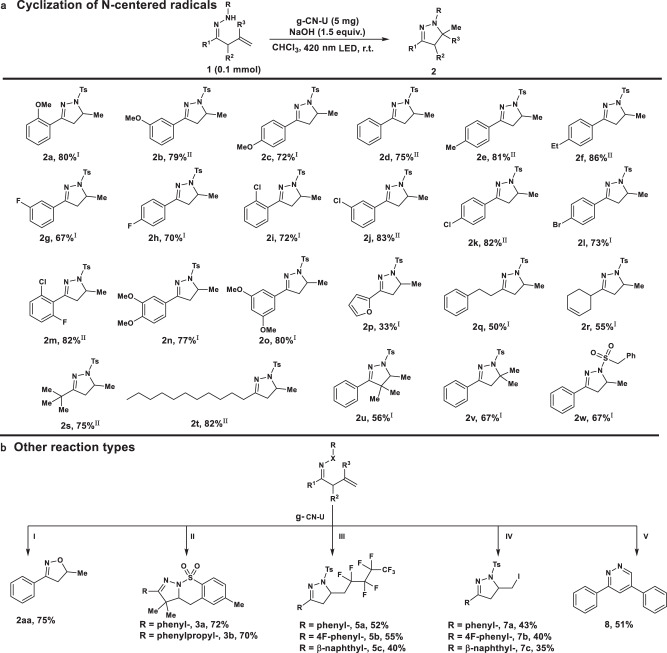
Substrate scope. **a** Substrate scope of β,γ-unsaturated hydrazones cyclization. Reaction conditions 1 (0.1 mmol), I: NaOH or II: LiOH (1.5 equiv.), g-CN-U (5 mg) in dry CHCl_3_ (2 mL) under N_2_ at room temperature for 8–12 h under a 3 W 420 nm LED. Isolated yields. **b** Other reaction types. I: under the 365 nm irradiation. II: [Co^III^(dmgH)_2_PyCl] as co-catalyst. III and IV: K_2_CO_3_ as base for 6 h. V: TEMPO (1.0 equiv.) was added. Details in the supplementary information.

The preparative scope of the reaction was investigated using a series of substituted β,γ-unsaturated hydrazones as the substrates. As illustrated in Fig. 5a, the reaction proceeded smoothly with the phenyl-derived substrates with either electron-donating (methyl, ethyl, methoxyl) or electron-withdrawing (F−, Cl−, Br−) groups at various positions (ortho, meta, para) in good yields (**2a**-**2l**). Bis-substituted β,γ-unsaturated hydrazone derivatives acted as competent cyclization substrates (**2m**–**2o**). The hydrazone containing a heterocyclic moiety was well tolerated to deliver the product in a 33% yield. Alkyl-substituted hydrazone, including primary, secondary, and tertiary aliphatic groups, proceed smoothly to furnish the desired products (**2q**–**2t**). The scope could be expanded to include hydrazone with alkyl groups at the α-position or terminal olefin moiety (**2** **u** and **2** **v**). N-Benzylsulfonyl-substituted hydrazones proved to be applicable to afford **2w** in 67% yield. Notably, the apparent quantum efficiency (AQE) at 420 nm for the photocatalytic production of **2a** is 7.47%, showcasing the outstanding capability of g-CN-U photocatalyst in the solar to chemical energy conversion.

As shown in Fig. 5b, the g-CN-U catalyst could promote the smooth photocatalytic cyclization of β,γ-unsaturated oxime (**2aa**). g-CN-U also proved to be a suitable photocatalyst in combination with the [Co] co-catalyst, delivering the fused tricyclic products in around 70-72% isolated yield (**3a**, **3b**)^51^. Beyond that, the g-CN-U exhibited satisfying activity toward radical olefin perfluoroalkylation with concomitant β-functionalization (**5a**–**5c**)^52^. And this result suggested the effective generation of one reactive perfluoroalkyl radical species over g-CN-U photocatalyst, which may directly couple with the carbon-centered radical intermediate or attack the electron-rich C-C double bond of the substrate to initiate the transformation (Supplementary Fig. 32). The selectivity of the transformation was switched with iodocyclohexane as the coupling substrate (**7a**–**7c**), and the halocyclization product was obtained as the major product^53^. Finally, we were delighted to find that the corresponding biologically important pyridazines, which usually required multiple synthetic steps to prepare, could be easily afforded through a 6-endo trig cyclization of the substrate with -Ph terminal substituent in good yield (**8**)^12^.

### Derivatization, scalability, and catalyst recyclization

**Fig. 6:**
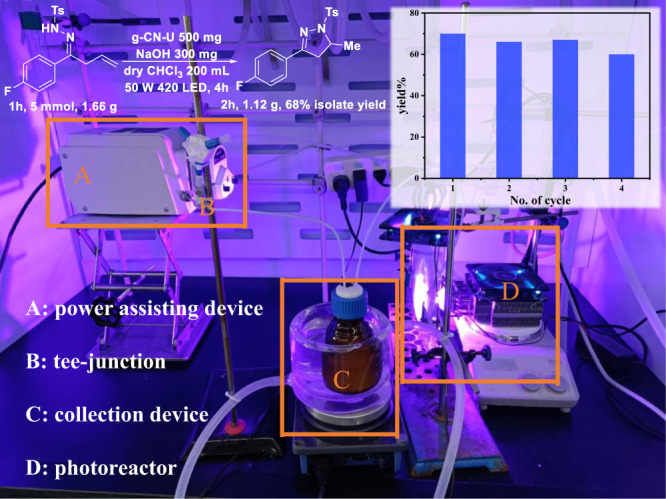
Gram-scale reaction with a continuous-flow setup and recycling of the photocatalyst. Reaction conditions **1** **h** (5 mmol, 1.66 g), NaOH (300 mg), g-CN-U (500 mg) in dry CHCl_3_ (200 mL) under N_2_ at room temperature for 4 h under a 50 W 420 nm LED. Isolated yields.

The dihydropyrazole product could be effectively transformed into the biologically significant N-H pyrazole derivatives in excellent yield (Supplementary Fig. 33). The photocatalytic process employing g-CN-U exhibited favorable scaling-up potential in terms of the reaction volume, reactant starting concentration, and stability (Fig. 6 and Supplementary Fig. 34). With the continuous flow protocol, the dihydropyrazole derivative could be obtained in 68% yield (<10% yield in batch) and the reaction period could be further reduced to only half. Furthermore, by recovering and reusing the g-CN-U photocatalyst for further characterizations and catalytic reactions, we were able to assess its stability and recyclability. The g-CN-U catalyst could be reused at least four times (Fig. 6) with only a slight attenuation of activity. There is no observed change in the crystal structure of the sample after the photocatalytic reaction, as demonstrated by the XRD, IR, and XPS characterization results (Supplementary Fig. 35-37). All the above results unambiguously confirmed the robustness and high efficiency of the metal-free g-CN-U photocatalyst for the photocatalytic cyclization process.

In conclusion, polymeric semiconductor g-CN-U is fabricated as an efficient and stable metal-free catalyst for the photocatalytic production of various pharmaceutically related heterocycles, including dihydropyrazoles, pyrazoles, tricyclic benzosultams, and pyradazines in high yields and broad substrate scope. The association between the characteristics of catalysts and their performances reveals that the interfacial coupling between catalyst and substrate is the key to the promotion of the reaction. g-CN-U with the surface complex systems could promote the highly selective oxidation of the N-H bond to nitrogen-centered radical with the synergistic combination of base and visible light, whereas g-CN-DCDA and K-PHI showed inferior activity. The formation of the chemisorbed substrate-g-CN-U surface complex is attributed to the abundant NH_2_ groups and the relative positive charged surface on g-CN-U, which could effectively couple with the deprotonated anionic hydrazone through electrostatic attraction and H-bonding interaction. This induces a shift in the oxidation potential of the hydrazone, making it easier to remove electrons and generate the nitrogen-centered radical. This work serves as a useful reference and source of inspiration for effectively modifying carbon nitride or other metal-free photocatalysts from a kinetics-based interfacial interaction perspective. This study also demonstrates that a green and mild strategy could be achieved for the one-pot synthesis of important pharmaceutical compounds using the carbon nitride photocatalytic system.

## Methods

### Synthesis of g-CN-U

10.0 g of urea as the precursor was removed into a covered crucible and heated in a muffle furnace from room temperature to 550 °C for 2 h, followed by a further heat treatment at 550 °C for 2 h. After that, the polymer is cooled to room temperature naturally. The obtained faint yellow solid was washed with hot deionized water and ethanol three times, dried out, and collected. This sample is denoted as g-CN-U.

### Synthesis of g-CN-DCDA

10.0 g of dicyandiamide as the precursor was removed into a covered crucible and heated in a muffle furnace from room temperature to 550 °C for 2 h, followed by a further heat treatment at 550 °C for 2 h. After that, the polymer is cooled to room temperature naturally. The obtained faint yellow solid was washed with hot deionized water and ethanol three times, dried out, and collected. This sample is denoted as g-CN-DCDA.

### Synthesis of K-PHI

0.60 g of the pre-heated carbon nitride (melamine as the monomer and heated to 550 °C in a muffle furnace for 2 h and lasted for 2 h) was fully mixed with KCl (3.30 g) and LiCl (2.70 g). Then, the mixture was heated to 550 °C with 4 ^o^C /min for 4 h under an N_2_ atmosphere. After that, the product was washed with hot deionized water several times to remove salts and collected by filtration, followed by drying at 60 °C under vacuum. This sample is denoted as K-PHI.

### Cyclization of β,γ-unsaturated hydrazone

A solution of β,γ-Unsaturated hydrazone **1** (0.1 mmol, 1 equiv.), bulk carbon nitride g-CN-U (5 mg), anhydrous sodium hydroxide, or lithium hydroxide (1.5 equiv.) in 2 mL dry chloroform were added into a Schlenk tube equipped with a stirring bar. The Schlenk tube was degassed in vacuo and purged with nitrogen five times. After finishing the freeze-pump-thaw process for several cycles, the reaction mixture was irradiated with a 3 W 420 nm LED at 25 °C for 8 ~ 12 h. After completion of the reaction monitored by TLC, the crude product was purified by short flash chromatography on silica gel (petroleum ether/ethyl acetate 12:1~8:1) to give the aim product in separation yields.

### Cyclization of β,γ-unsaturated oxime

A solution of β,γ-Unsaturated oxime **1aa** (0.1 mmol, 1 equiv.), bulk carbon nitride g-CN-U (5 mg), anhydrous sodium hydroxide, or lithium hydroxide (1.5 equiv.) in 2 mL dry chloroform were added into a Schlenk tube equipped with a stirring bar. The Schlenk tube was degassed in vacuo and purged with nitrogen five times. After finishing the freeze-pump-thaw process for several cycles, the reaction mixture was irradiated with a 3 W 365 nm LED at 25 °C for 12 h. After completion of the reaction monitored by TLC, the crude product was purified by short flash chromatography on silica gel (petroleum ether/ethyl acetate 12:1~8:1) to give the product in separation yields.

### Synthesis of dihydropyrazole-fused benzosultams

A solution of **1** (0.1 mmol), g-CN-U (5.0 mg), K_2_CO_3_ (27.6 mg, 0.2 mmol), [Co^III^(dmgH)_2_PyCl] (4.0 mg, 0.01 mmol) in MeCN (2.0 mL) were dissolved into a Schlenk tube. Then, the mixture was degassed via the ‘freeze-pump-thaw’ procedure (3 times) under an argon atmosphere. After that, the solution was stirred at 50 W blue LEDs (420 nm) at 80 ^o^C for about 24 h until completion (monitored by TLC analysis). The crude product was purified by flash chromatography on silica gel (petroleum ether/ethyl acetate 10:1~3:1) directly to give the desired product **3** as a white solid.

## Supplementary information

Supplementary Information

## Data Availability

The authors declare that all data generated in this study are provided in the article and the Supplementary Information file, and are also available from the corresponding author upon request. Source data are provided with this paper.

## Source data

Source Data
