# Supplementary material for: Photocatalytic cyclization of nitrogen-centered radicals with carbon nitride through promoting substrate/catalyst interaction
Source: Nat Commun. 2022 Aug 20;13:4900. doi: 10.1038/s41467-022-32623-3 (PMC9392757; doi:10.1038/s41467-022-32623-3)

Supplementary Information

Photocatalytic Cyclization of Nitrogen-Centered Radicals with Carbon Nitride through Promoting Substrate/Catalyst Interaction

Mingcheng Yang[1], Ronghong Lian[1], Xirui Zhang[1], Chong Wang[1], Jiajia Cheng[1,2], and Xinchen Wang[1,2]

[1]State Key Laboratory of Photocatalysis on Energy and Environment, College of Chemistry, Fuzhou University, Fuzhou 350116, China.

[2]Qingyuan Innovation Laboratory, Quanzhou 362801, China.

Correspondence and requests for materials should be addressed to J.C. (email: jjcheng@fzu.edu.cn) or to X. W. (email: xcwang@fzu.edu.cn)

Contents

Supplementary Experimental Details 3
Supplementary Figures and Tables 6
General Procedure for the Photocatalytic Reaction 32
Characterization of Products 35
Supplementary References 50
NMR Spectra 52

Supplementary Experimental Details

General information.

Powder X-ray diffraction (XRD) measurements were performed on Bruker D8 Advance diffractometer. UV-vis diffuse reflectance spectra (DRS) were performed on Agilent Technologies Cary 500. The X-ray photoelectron spectroscopy (XPS) data were obtained using a Thermo ESCALAB250 instrument. Solid-state ^{13}C NMR experiments were acquired on JEOL RESONANCE 600M spectrometer. The Fourier transform infrared (FTIR) analyses were recorded on a BioRad FTS 6000 spectrometer. Fluorescence (PL) spectra and time-resolved PL was measured in Edinburgh FL-FS 920 TCSPC. The electron paramagnetic resonance (EPR) measurements were carried out on a Bruker Model A300 spectrometer. Thermogravimetric analysis (TG) was performed on the Netzsch STA 449F3 simultaneous thermal analyzer. Temperature programmed desorption (TPD) measurements were carried out on a Micromeritics AutoChem Ⅱ 2920 Chemisorption Analyzer.The Brunauer-Emmett-Teller (BET), and surface area (SBET) of the samples were determined by N_2 adsorption using a Micromeritics ASAP 2046 N_2 adsorption apparatus (USA). All reactions were carried out with dry solvents under an atmosphere of argon using standard Schlenk techniques unless otherwise stated. Column chromatographic purification of products was accomplished using 200-300 mesh silica gel. ^{1}H and ^{13}C NMR spectra were recorded on a JEOL RESONANCE 600M spectrometer in the solvents indicated (note: $CDCl_3$ referenced at 7.26 and 77.00 ppm respectively); Coupling constants are reported in Hz with multiplicities denoted as s (singlet), d (doublet), t (triplet), q (quartet) and m (multiplet). High-resolution mass spectra were recorded on Exactive Plus LC-MS (ESI) mass spectrometers.

Electrochemical Measurements.

Electrochemical measurements were conducted with a BAS Epsilon Electrochemical System in a conventional three-electrode cell, using a Pt plate as the counter electrode and an Ag/AgCl electrode (3 M KCl) as the reference electrode. The working electrode

was prepared by dip-coating sample slurry on ITO glass and then dried at room temperature.

AQE measurement.

To determine the apparent quantum efficiency (AQE) of g-CN-U, the cycloaddition photoreaction was conducted on the same device at room temperature, using a 420 LED lamp as the light source. The light intensity was measured at 31.7 mW cm^{-2} and the irradiated area was controlled at 1 cm^2. After reaction for 1h, the product **1a** was 31% yield determined by crude NMR (benzyl oxide as the internal standard). The calculation processes of AQE are shown below:

$$\text{AQE} = \frac{\text{the number of reacted electrons}}{\text{the number of incident photos}} \times 100\% = \frac{\text{Ne}}{\text{Np}} = \frac{\text{M}\times\text{N}_\text{A}\times\text{h}\times\text{c}}{\text{S}\times\text{P}\times\text{t}\times\lambda} \times 100\% = 7.47\%$$

where N_e and N_p stand for the number of electrons involved in the reduction and incident photons, n is the number of electrons needed to form a product molecule, M is the molar number of products. N_A and h represent Avogadro's constant and Planck's constant, respectively. W, S, t, and υ are the light density, irradiation area, time, and light frequency, respectively.

Synthesis of the g-CN-U-b, g-CN-DCDA-b, K-PHI-b and H-PHI samples.

g-CN-U-b: 100 mg g-CN-U was dispersed equably in 100 mL potassium hydroxide solution (1 M). After stirring for 1 h, the carbon nitride sample was filtrated, rinsed with deionized water, followed by drying at 70 °C under vacuum. This sample is denoted as g-CN-U-b.

g-CN-DCDA-b: 100 mg g-CN-DCDA was dispersed equably in 100 mL potassium hydroxide solution (1 M). After stirring for 1 h, the sample was filtrated, rinsed with deionized water, followed by drying at 70 °C under vacuum. This sample is denoted as g-CN-DCDA-b.

K-PHI-b: 100 mg K-PHI was dispersed equably in 100 mL potassium hydroxide solution (1 M). After stirring for 1 h, the carbon nitride sample was filtrated, rinsed with deionized water, followed by drying at 70 °C under vacuum. This sample is denoted as K-PHI-b.

H-PHI: 100 mg K-PHI was dispersed equably in 100 mL 0.2 M HCl solution. After stirring for 0.5 h, the carbon nitride sample was filtrated, rinsed with deionized water, followed by drying at 70 °C under vacuum. This sample is denoted as H-PHI.

Details of theoretical calculations

All calculations were carried out using density functional theory (DFT) by employing the Vienna Ab-initio Simulation Package (VASP) code.[1] Nuclei and core electrons were described by the projector augmented wave (PAW) potentials.[2] The generalized gradient approximation of Perdew-Burke-Ernzerhof (PBE) exchange-correlation functional was employed combined with the Grimme's DFT-D3 correction.[3-5] A Monkhorst–Pack mesh of 3×3×3 *k*-points was used in the Brillouin zone for geometry optimizations and electronic structure calculations. The cut-off energy of the plane-wave expansion was set to 550 eV, and the convergence thresholds of the energy change and the maximum force were set to 10^{-5} eV and 0.01 eV/Å, respectively. A vacuum space was set to 20 Å to avoid the interactions between neighboring molecules. The Bader Charge Analysis code was used for the population analysis.[6-9]

Supplementary Figures and Tables

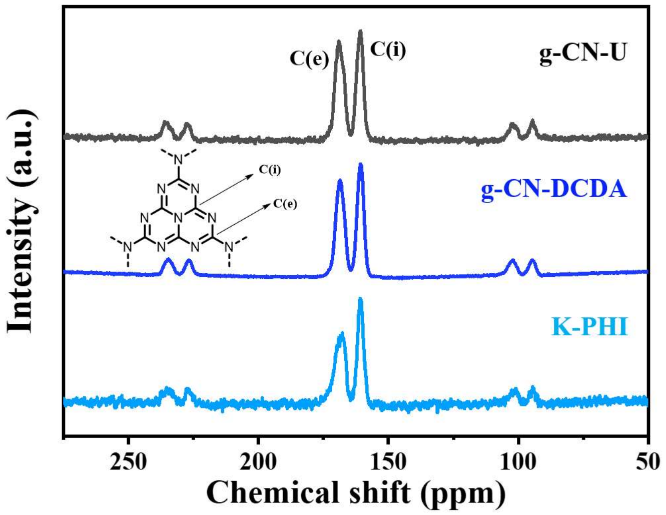

Supplementary Figure 1. Catalysts NMR characterization. The solid-state ^{13}C NMR spectra of g-CN-U, g-CN-DCDA and K-PHI.

Entry	Sample	C, wt.%	N, wt.%	H, wt.%	K[a], wt.%	K, moles	C/N/H/K
1	g-CN-DCDA	35.28	62.26	1.75	0	0	$C_3N_{4.5}H_{1.78}$
2	g-CN-U	34.51	62.67	2.12	0	0	$C_3N_{4.66}H_{2.21}$
3	K-PHI	26.87	44.19	1.40	14.8	0.38	$C_3N_{4.22}H_{1.87}K_{0.51}$

Supplementary Table 1. Elemental analysis of catalysts. Elemental composition of all g-CN-U, g-CN-DCDA and K-PHI ([a]ICP-AES analysis).

Supplementary Table 2. ICP-AES analysis of catalysts. The content of various metals in the samples.

Metal content (wt.%)	g-CN-U	g-CN-DCDA	K-PHI
Li	0	0	0
K	0	0	14.8
Mn	0	0	0
Co	0	0	0
Ni	0	0	0
Cu	0	0	0
Ru	0	0	0
Pd	0	0	0

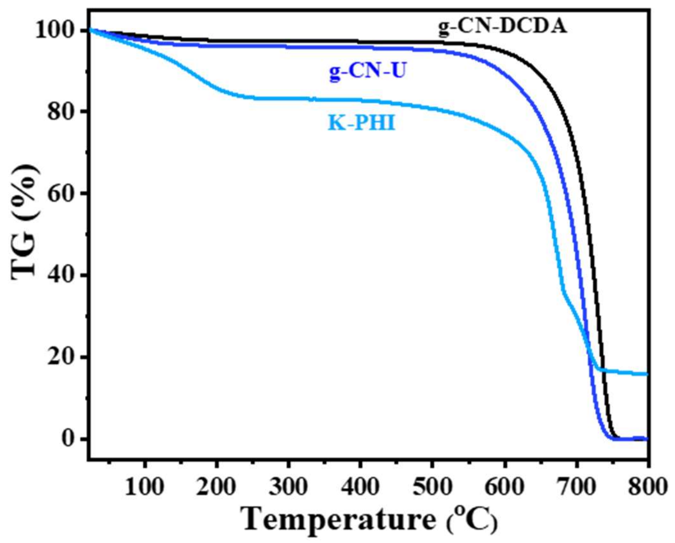

Supplementary Figure 2. Thermostability study of catalysts. Thermogravimetric analysis of g-CN-U, g-CN-DCDA and K-PHI.

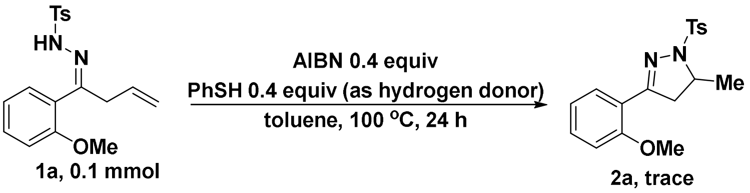

Supplementary Figure 3. Cyclization reaction with the addition of a radical initiator under heating conditions. A solution of **1a** (0.1 mmol, 34.4 mg), AIBN (0.04 mmol, 6.6 mg), PhSH (0.04 mmol, 4.4 mg) and 1 mL toluene was added into a Schlenk tube. The resulting mixture was then degassed via the 'freeze-pump-thaw' procedure (3 times) under argon atmosphere and stirred under the 100 °C for 24 h. After completion, the experimental results was determined by crude ^{1}H-NMR.

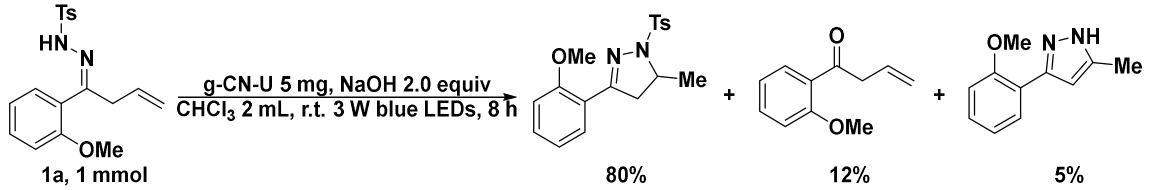

Supplementary Figure 4. Photocatalytic transformation of 1a. 1a (0.1 mmol, 34.4 mg), g-CN-U 5 mg and NaOH (0.2 mmol, 8.0 mg) were dissolved in chloroform (2.0 mL). The resulting mixture was then degassed via the 'freeze-pump-thaw' procedure (3 times) under argon atmosphere. After that, the solution was stirred at 3 W blue LEDs (420 nm) at room temperature for 8 h. When finished, the experimental results was determined by crude ^{1}H-NMR.

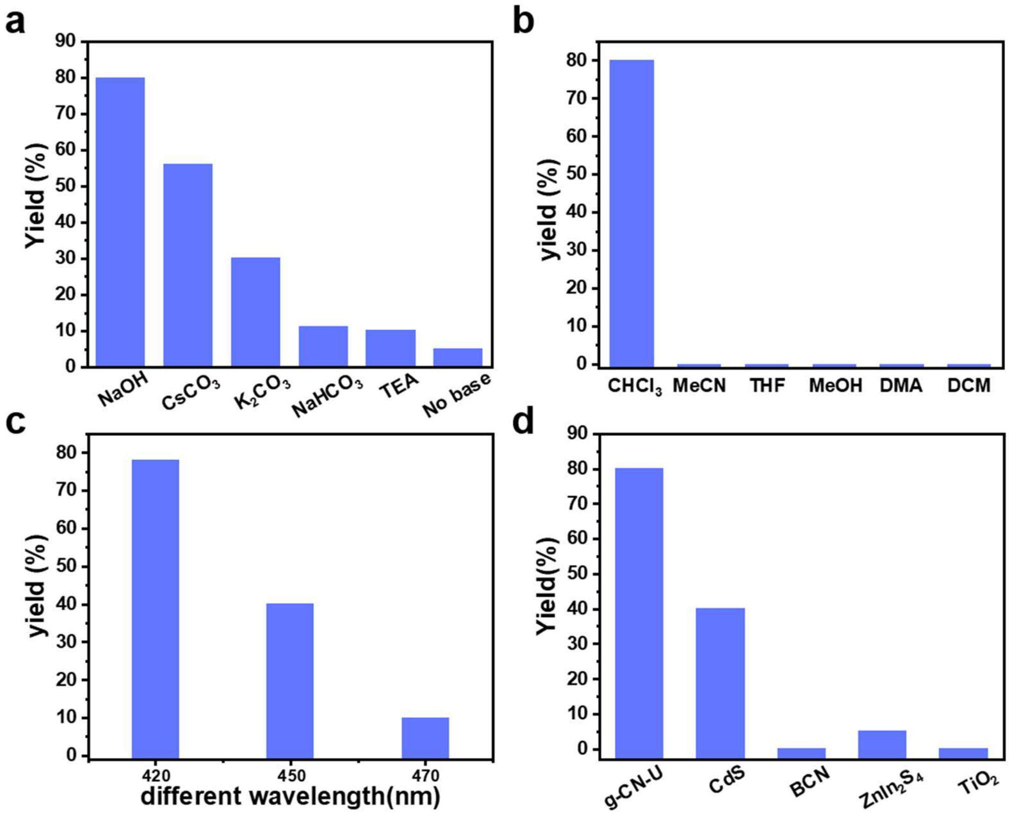

Supplementary Figure 5. Photocatalytic reaction under conditions of various conditions. (a) bases, (b) solvents, (c) light wavelength, (d) photocatalysts. Reaction conditions: **1a** (0.1 mmol), g-CN-U (5 mg), NaOH (1.5 equiv.), $CHCl_3$ (2 mL), 3 W 420 nm LED, under N_2 at room temperature for 8 h. Yields determined by ^1H NMR using benzyl ether as an internal standard.

Supplementary Table 3. Catalytic activity comparison. Selected reports for the catalytic synthesis of dihydropyrazole derivatives.

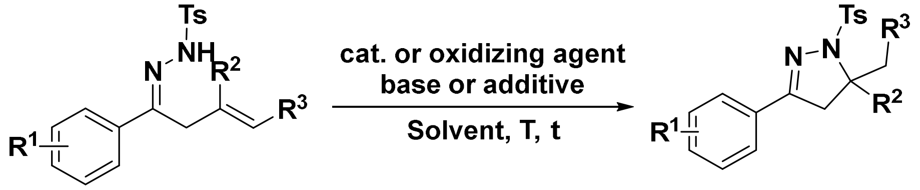

entry	light	cat./[O]	additive	$R^1/R^2/R^3$	solvent	temp.	time	yield	ref.
1	No	$PhI(OAc)_2$	DABCO	H/H/H	THF	RT	2 h	65%	10
2	No	$Mn(dpm)_3$ *t*BuOOH	-	H/H/H	*i*PrOH	55 °C	5 h	55%	11
3	No	H_2SO_4	-	Me/Me/H	MeCN	50 °C	12 h	73%	12
4	No	$PhI(OAc)_2$	DBU	OMe/H/S-Ph	MeCN	RT	6 h	79%	13
5	No	Co(III) complexes	NBS	H/H/-Br	toluene	-30 °C	24 h	77%	14
6	No	CuI	$NaBH_4$	H/H/-OH	CH_2Cl_2	RT	2 h	76%	15
7	No	sulfamide	NBS	H/H/-Br	heptane	RT	2 h	68%	16
8	No	$Cu(acac)_2$ $K_2S_2O_8$	$NaHCO_3$	H/H/CN	DMSO	RT	12 h	76%	17
9	No	$Pd(TFA)_2$	DIPEA	H/H/Ph	toluene	RT	24 h	78%	18
10	No	TBHP/I_2	$NaHCO_3$	F/H/Ts	dioxane	RT	20 h	65%	19
11	No	$BF_3{\cdot}OEt_2$	-	Me/H/S-Ph	DCE	RT	18 h	57%	20
12	No	$Cu(acac)_2$ $K_2S_2O_8$	$NaHCO_3$	Cl/H/SCN	DMSO	RT	17 h	61%	21
13	No	$Pd(OAc)_2$	$NaHCO_3$	OMe/H/COPh	THF	RT	24 h	78%	22
14	No	$PhI(OAc)_2$	$BF_3{\cdot}OEt_2$	*t*Bu/H/NHAc	MeCN	RT	18 h	43%	23
15	No	selectfluor	K_2HPO_4	H/CH_2F/H	MeCN	100 °C	1 h	19%	24
16	No	CuOAc	CsF and TCCA	Me/H/CF_3	MeCN	RT	2.5 h	65%	25
17	Yes	$[Ru(bpy)_3]Cl_2{\cdot}6H_2O$	$NaHCO_3$	Me/Ph/CF_3	MeCN	RT	18h	76%	26
18	Yes	$[Ru(bpy)_3]Cl_2{\cdot}6H_2O$	NaOH	H/H/H	$CHCl_3$	RT	12 h	80%	27, 28
19	Yes	Acridine salt TEMPO	K_2CO_3	Cl/H/OH	MeCN	RT	12 h	78%	29
**20**	**Yes**	**g-CN-U**	**NaOH**	**OMe/H/H**	**$CHCl_3$**	**RT**	**8 h**	**80%**	**This work**

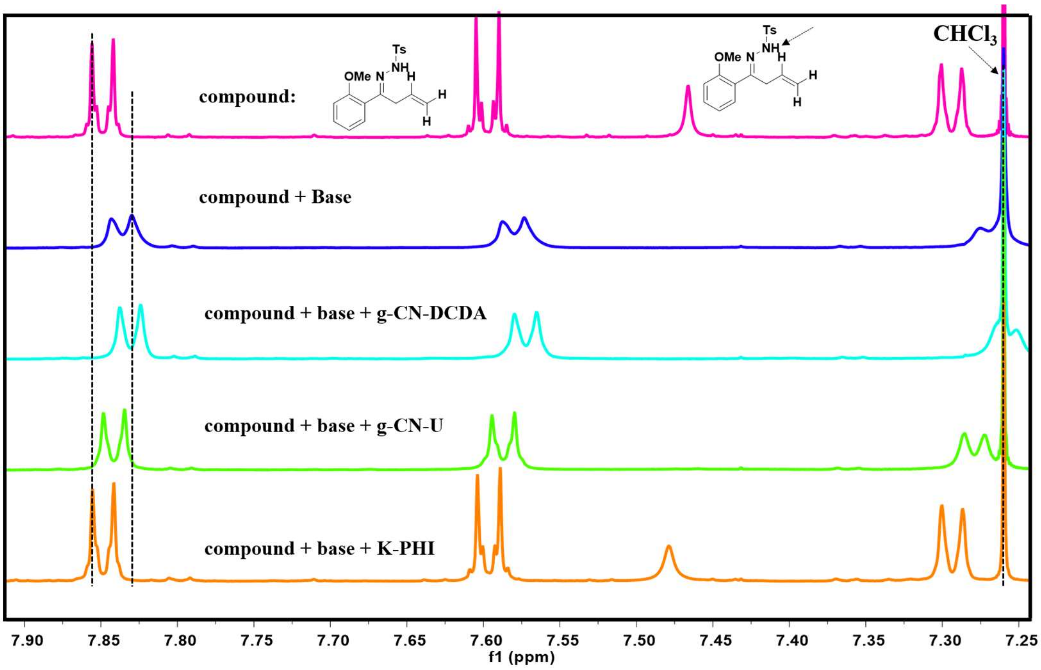

Supplementary Figure 6. ^{1}HNMR spectra of the reaction mixture. Interaction of various carbon nitrides with base and substrate.

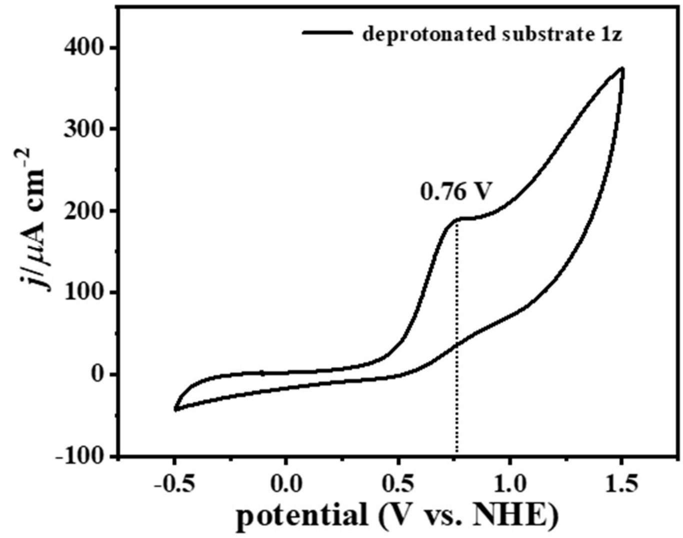

Supplementary Figure 7. CV of deprotonated substrate 1z. conditions: 0.1 M tetrabutylammonium tetrafluoroborate, **1z** (2 mM), NaOH (2 mM) in CH_3CN using a glassy carbon as working electrode, a platinum wire as a counter electrode, a silver wire as pseudo reference, ferrocene as internal standard and reference electrode at 20 mV/s scan rate.

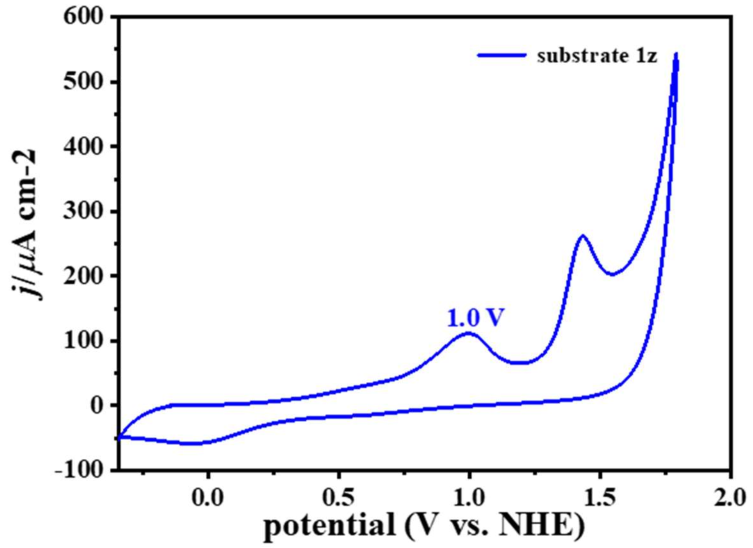

Supplementary Figure 8. CV of substrate 1z. conditions: tetrabutylammonium tetrafluoroborate, substrate **1z** (2 mM) in CH_3CN using a glassy carbon as working electrode, a platinum wire as a counter electrode, a silver wire as pseudo reference, ferrocene as internal standard and reference electrode at 20 mV/s scan rate.

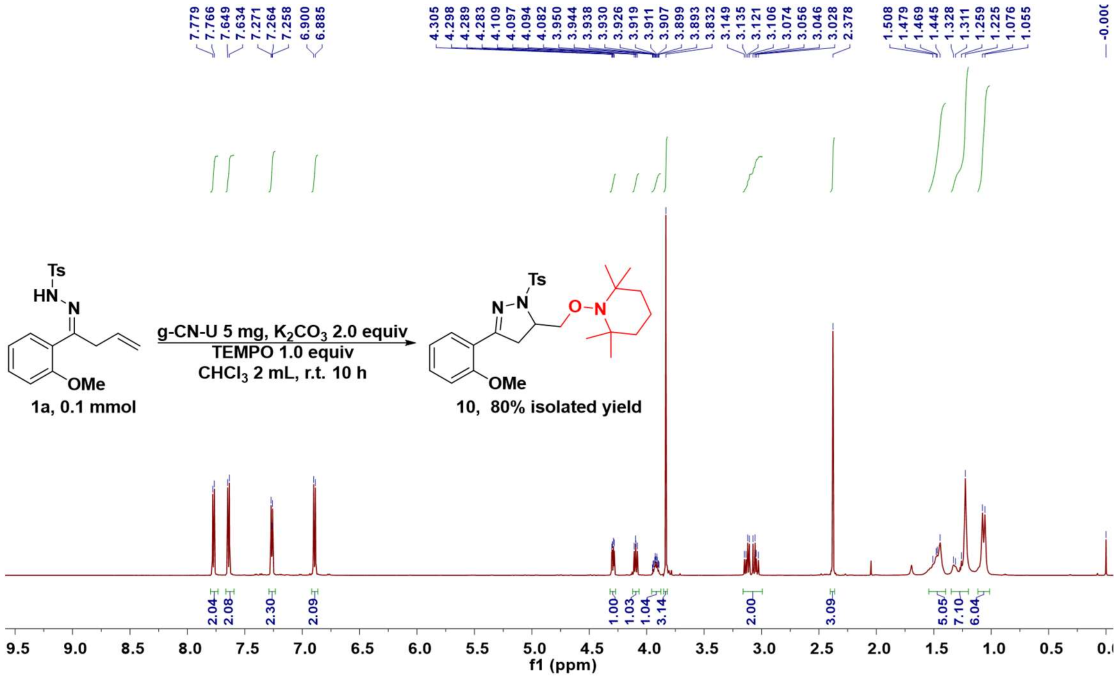

Supplementary Figure 9. Radical trapping experiment. ^{1}HNMR spectra of the isolated TEMPO-trapping product 10 (80% isolated yield).

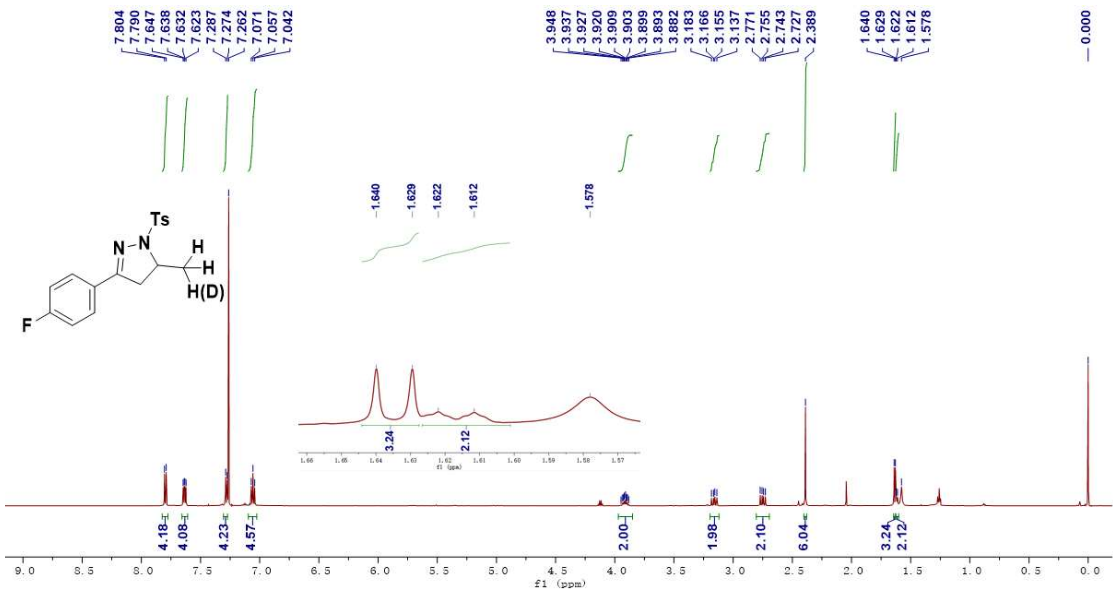

Supplementary Figure 10. Deuterium-labeling experiment. ^{1}HNMR of the experiment conducted in $CDCl_3$ (2h and 2h' in 1:1 ratio).

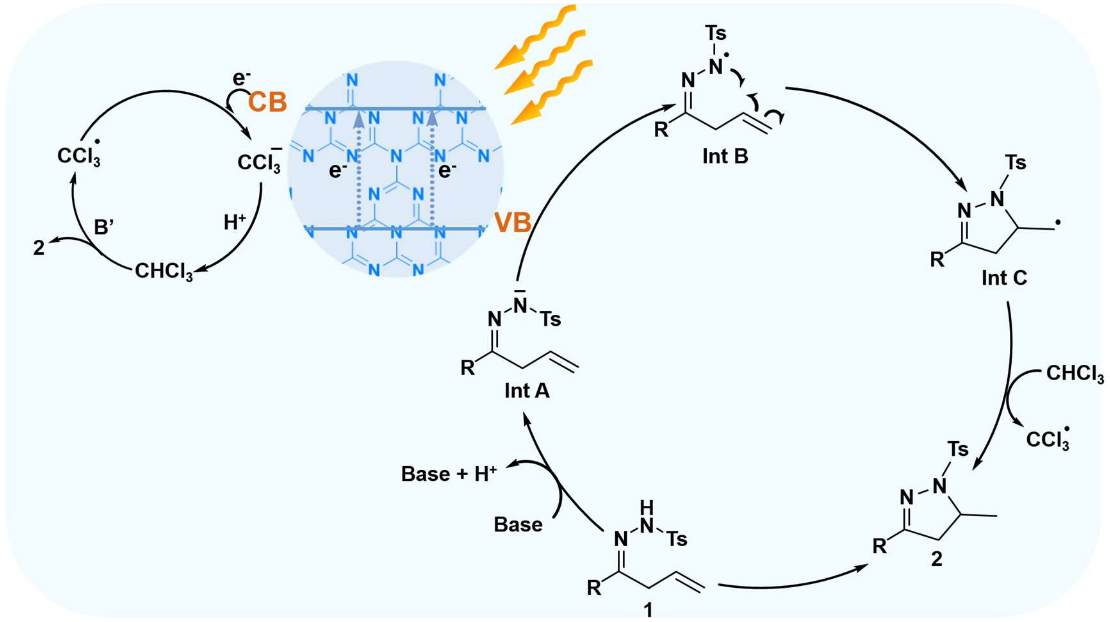

Supplementary Figure 11. Proposed reaction mechanism. Photocatalytic cyclization of nitrogen-centered radicals for dihydropyrazole synthesis over carbon nitride.

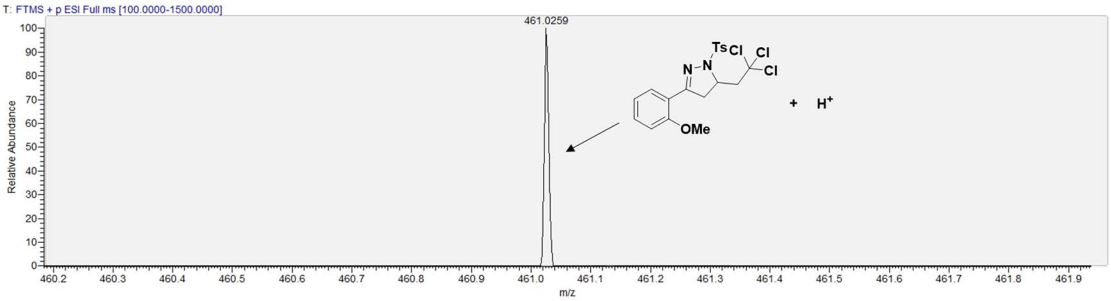

Supplementary Figure 12. Radical trapping experiment. HRMS of trichloromethyl substituted dihydropyrazole derivative.

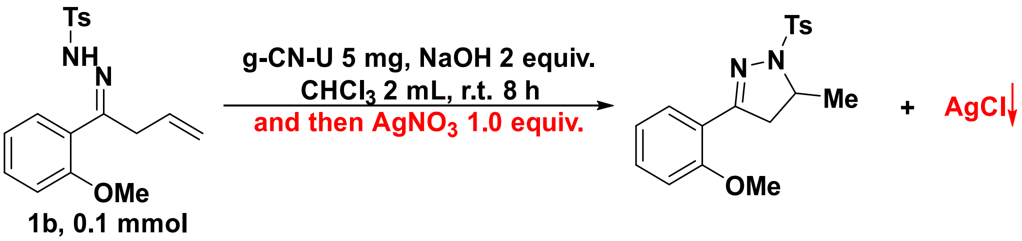

Supplementary Figure 13. Chloride trapping experiment. the unstable trichloromethanide anions could undergo decomposition to afford the dichlorocarbene species and chloride ions. Details: To a dry Schlenk tube equipped with a magnetic stir bar, was added β,γ-unsaturated hydrazone **1a** (0.1 mmol), g-CN-U (5 mg), NaOH (8 mg, 2 equiv.), and $CHCl_3$ (2 mL). Then, the resulting mixture was degassed via the 'freeze-pump-thaw' procedure (3 times) under argon atmosphere. After that, the solution was stirred at 3 W blue LEDs (420 nm) at room temperature for 8 hours. After completion of the reaction, the solid catalyst was removed from the reaction system and 1 mL $AgNO_3$ aqueous solution (0.1 M) was added to the crude solution. A white floccule on the interface between the aqueous phase and organic phase could be observed. The white floccule was not dissolved in the HNO_3 solution and disappeared in ammonia water, which could be identified as AgCl solid.

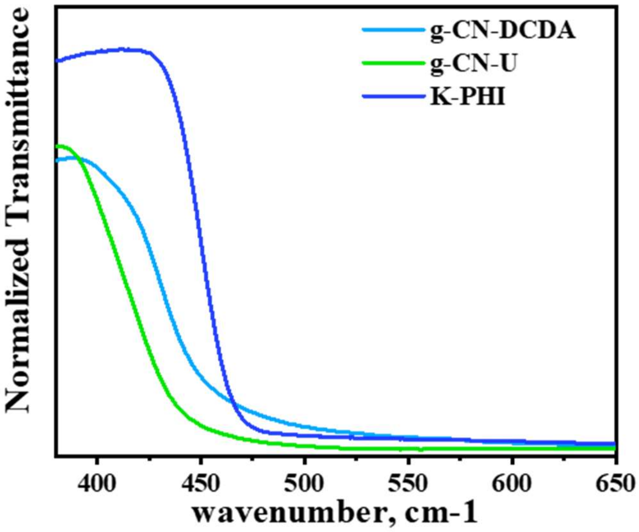

Supplementary Figure 14. Optical absorption of the prepared catalysts. UV-Vis diffuse reflectance spectra of g-CN-DCDA, g-CN-U, K-PHI.

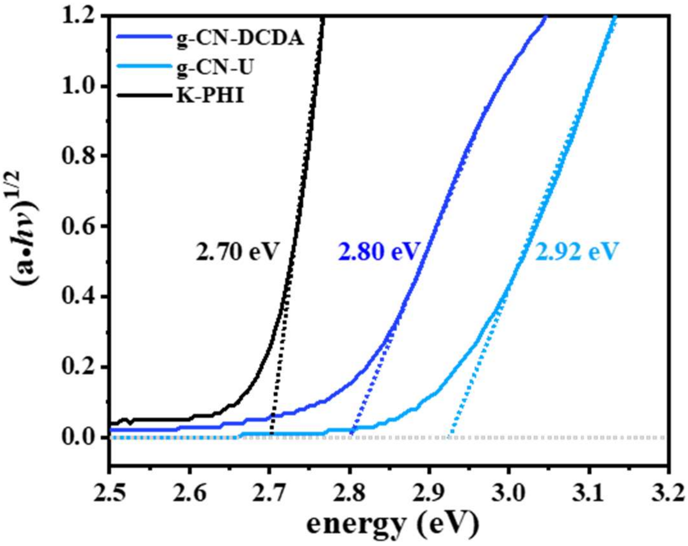

Supplementary Figure 15. Tauc plots for bandgap determination. Plots of $(\alpha h\nu)^{1/2}$ versus energy (*hν*) for the bandgap energies of different carbon nitrides.

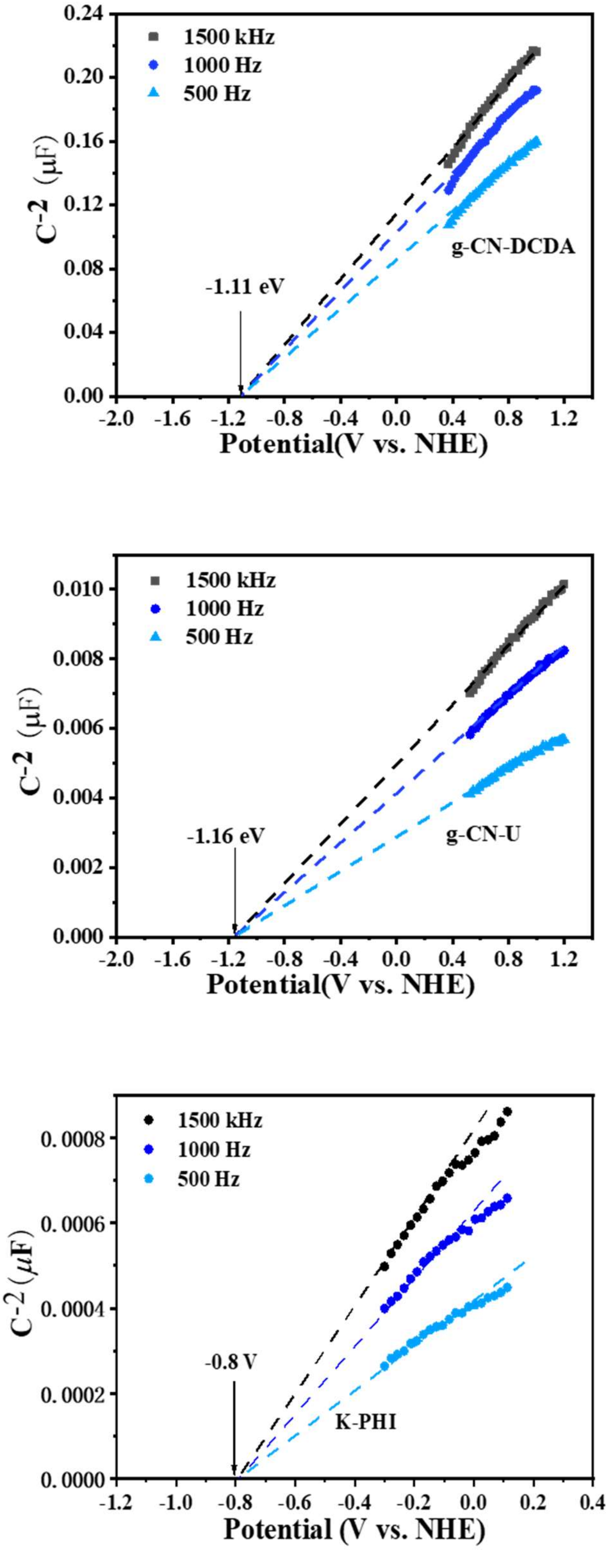

Supplementary Figure 16. Mott-Schottky plots. Different carbon nitrides were tested with the frequency of 1500 Hz, 1000 Hz, and 500 Hz.

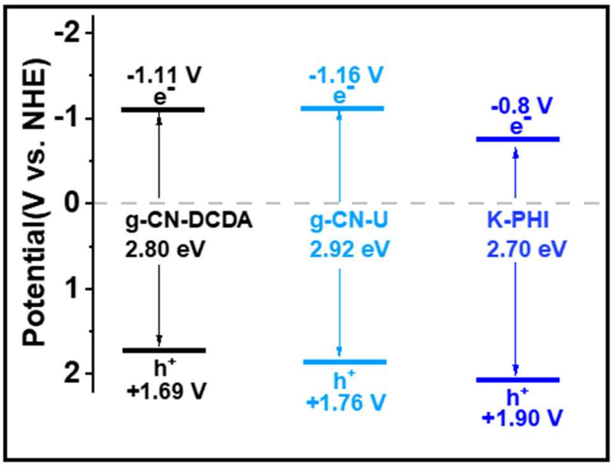

Supplementary Figure 17. Band alignment of the catalysts. Electronic band structure of g-CN-DCDA, g-CN-U, and K-PHI.

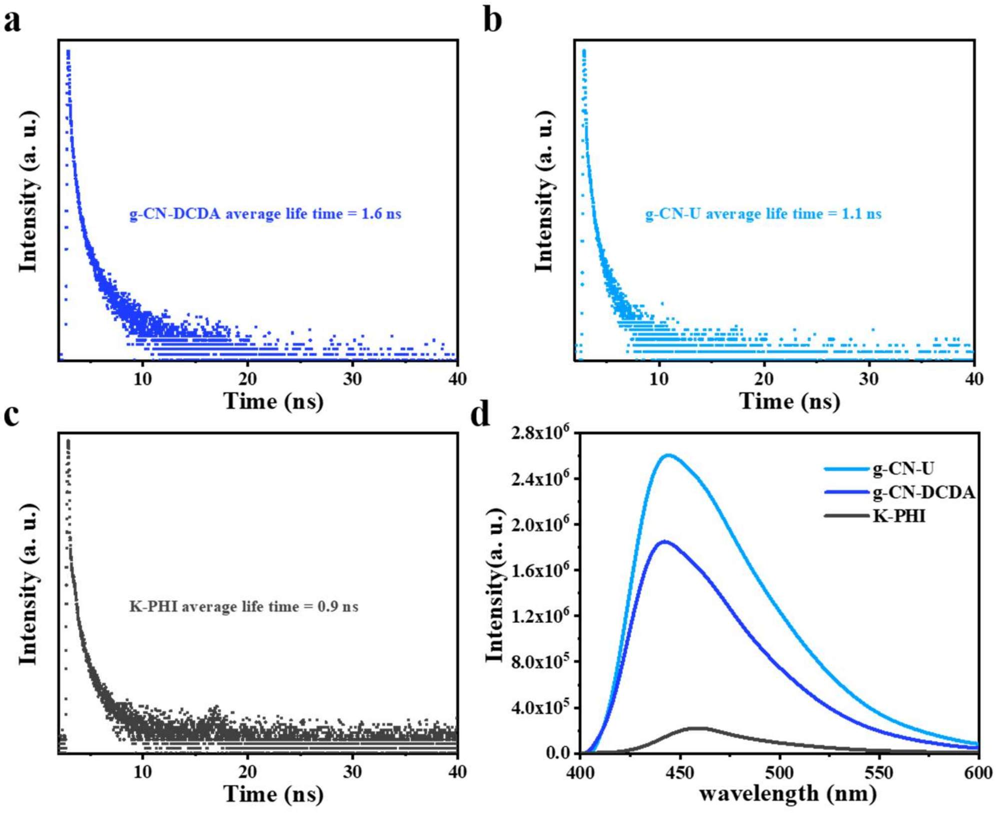

Supplementary Figure 18. The excitonic processes of photo-generated charges. (a) Time-resolved PL spectra of g-CN-DCDA, (b) g-CN-U, (c) K-PHI, (d) photoluminescence spectra of different carbon nitrides.

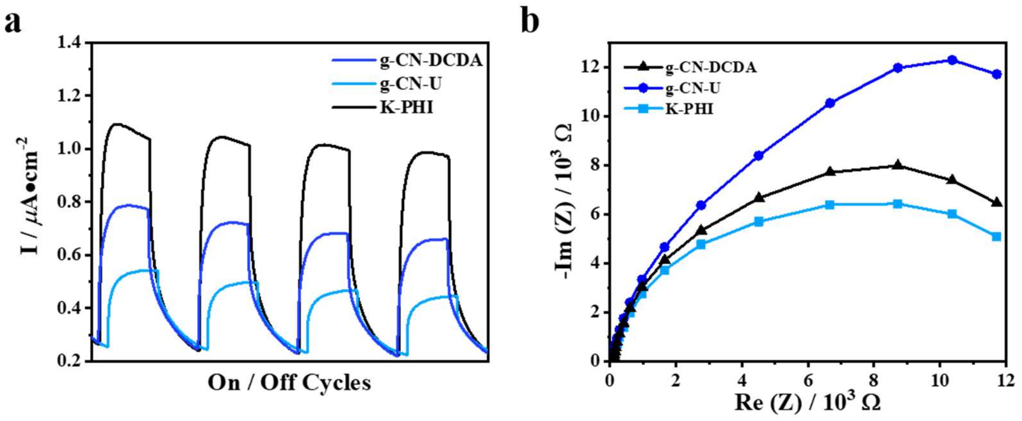

Supplementary Figure 19. The photo-electrochemical experiments. (a) Transient photocurrent density and (b) electrochemical impedance spectra of g-CN-DCDA, g-CN-U, K-PHI.

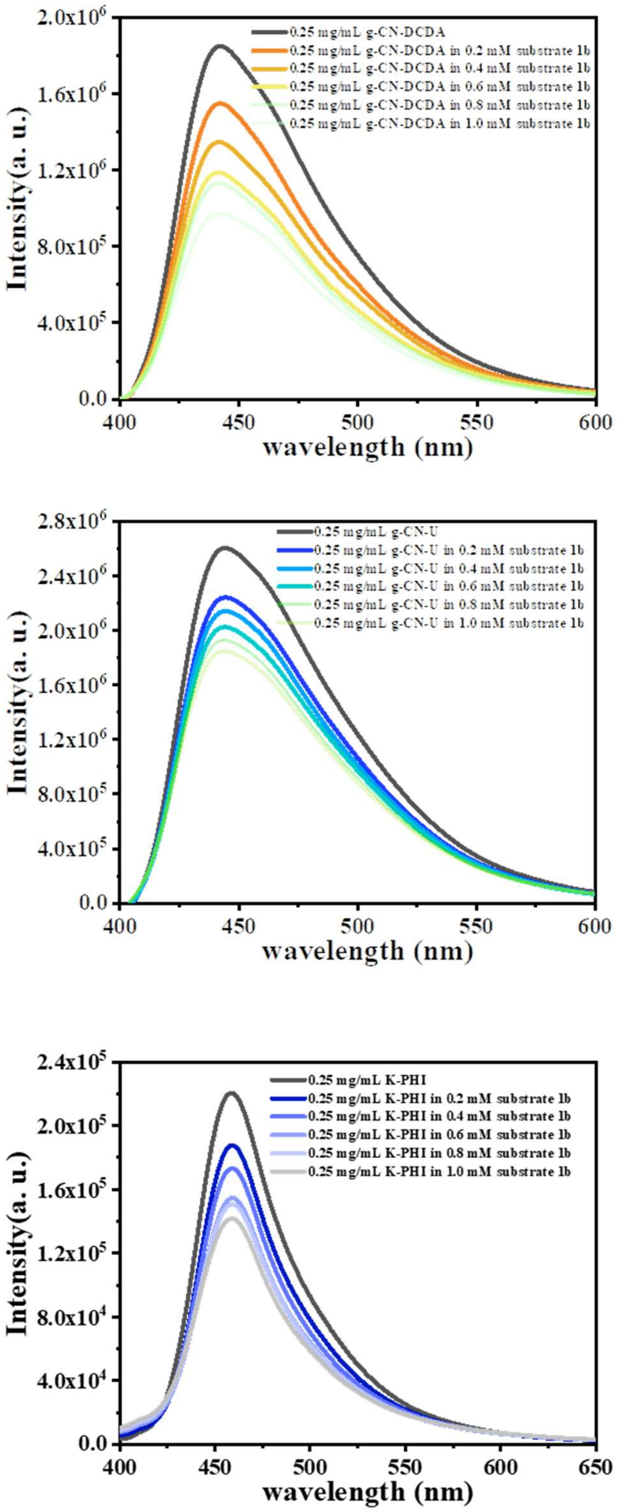

Supplementary Figure 20. Emission quenching experiments. The same concentrations of g-CN-DCDA, g-CN-U, K-PHI were quenched with various concentrations of deprotonated substrate **1a**. Excitation wavelength = 390 nm.

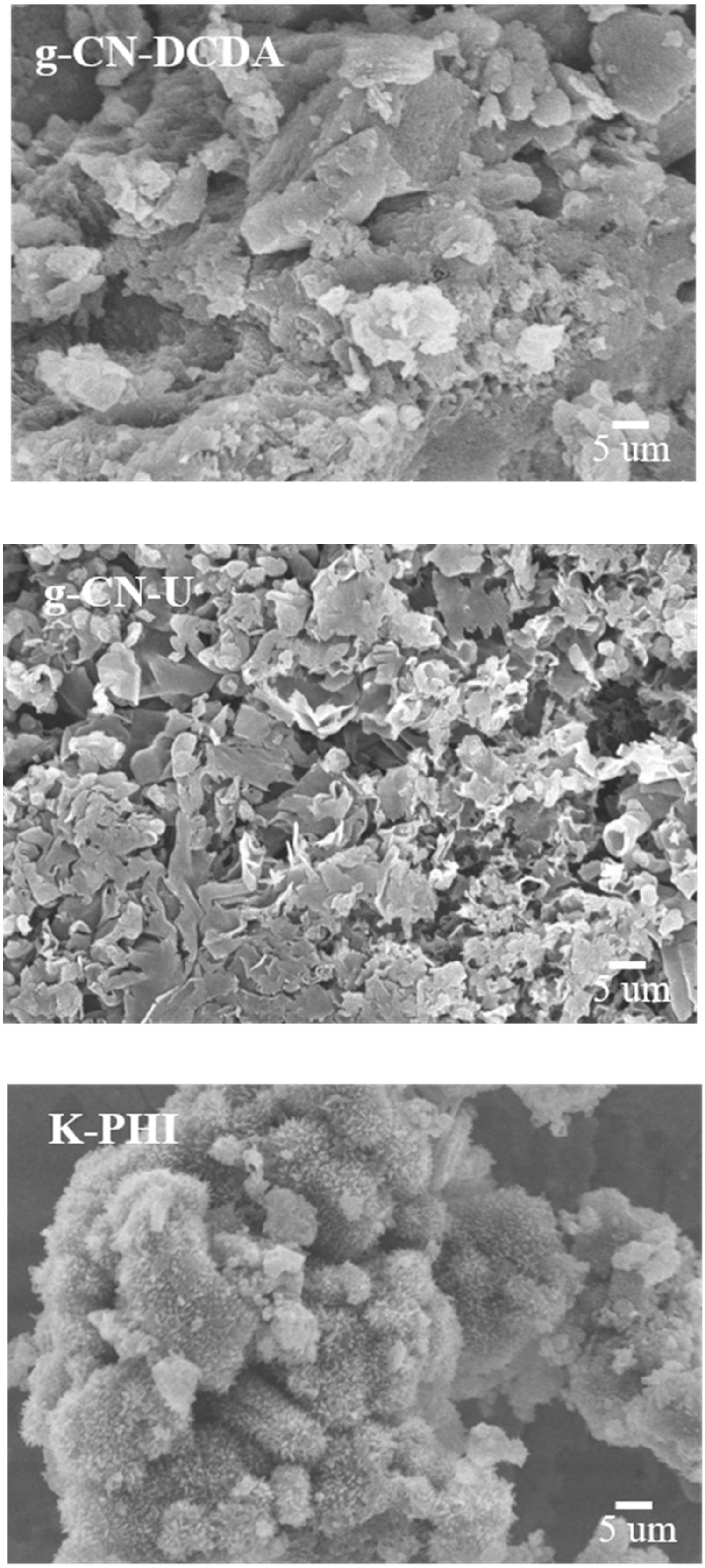

Supplementary Figure 21. Catalysts morphology characterization. Scanning electron microscopy of the carbon nitride catalysts.

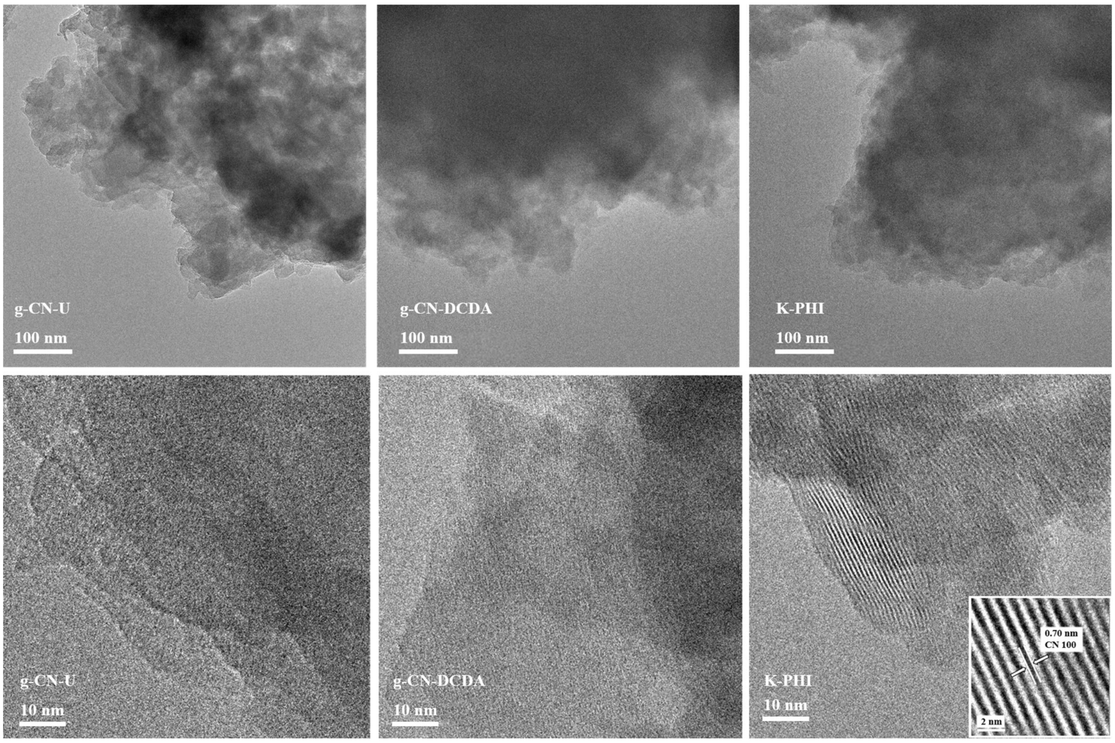

Supplementary Figure 22. TEM images of the catalysts. High resolution TEM images of g-CN-U, g-CN-DCDA and K-PHI.

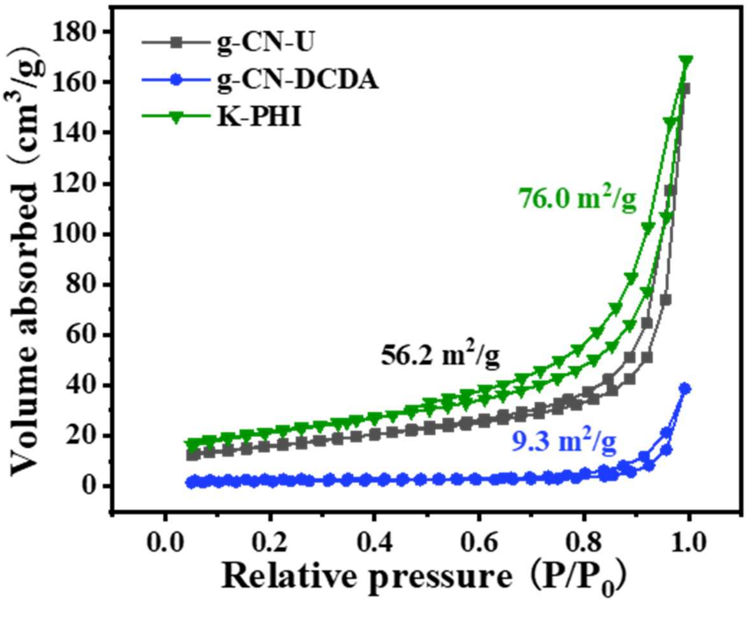

Supplementary Figure 23. Specific surface area measurement of the catalysts. Nitrogen adsorption-desorption isotherms of g-CN-DCDA, g-CN-U, and K-PHI.

Supplementary Table 4. Average pores volume of the catalysts. The results of the prepared samples were determined by the BJH method.

Samples	g-CN-U	g-CN-DCDA	K-PHI
Pores volume (cm^3/g)	0.24	0.06	0.20

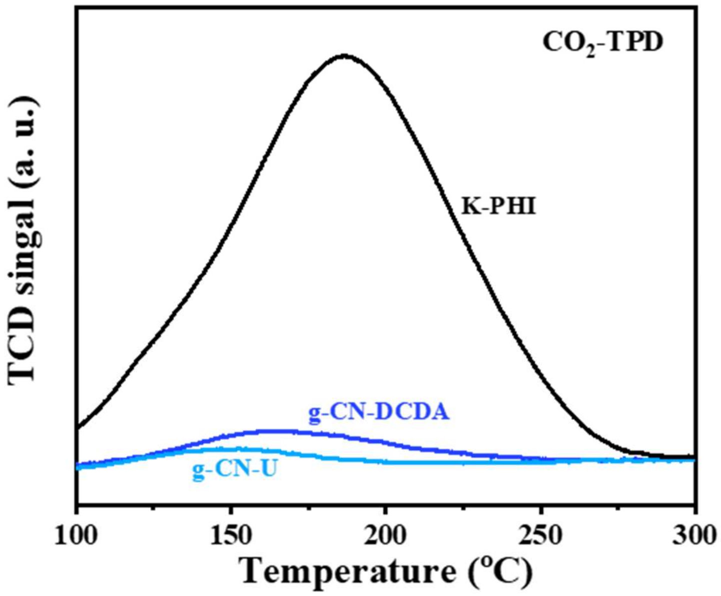

Supplementary Figure 24. Temperature programmed desorption of the catalysts. CO_2-TPD profiles of g-CN-DCDA, g-CN-U, and K-PHI.

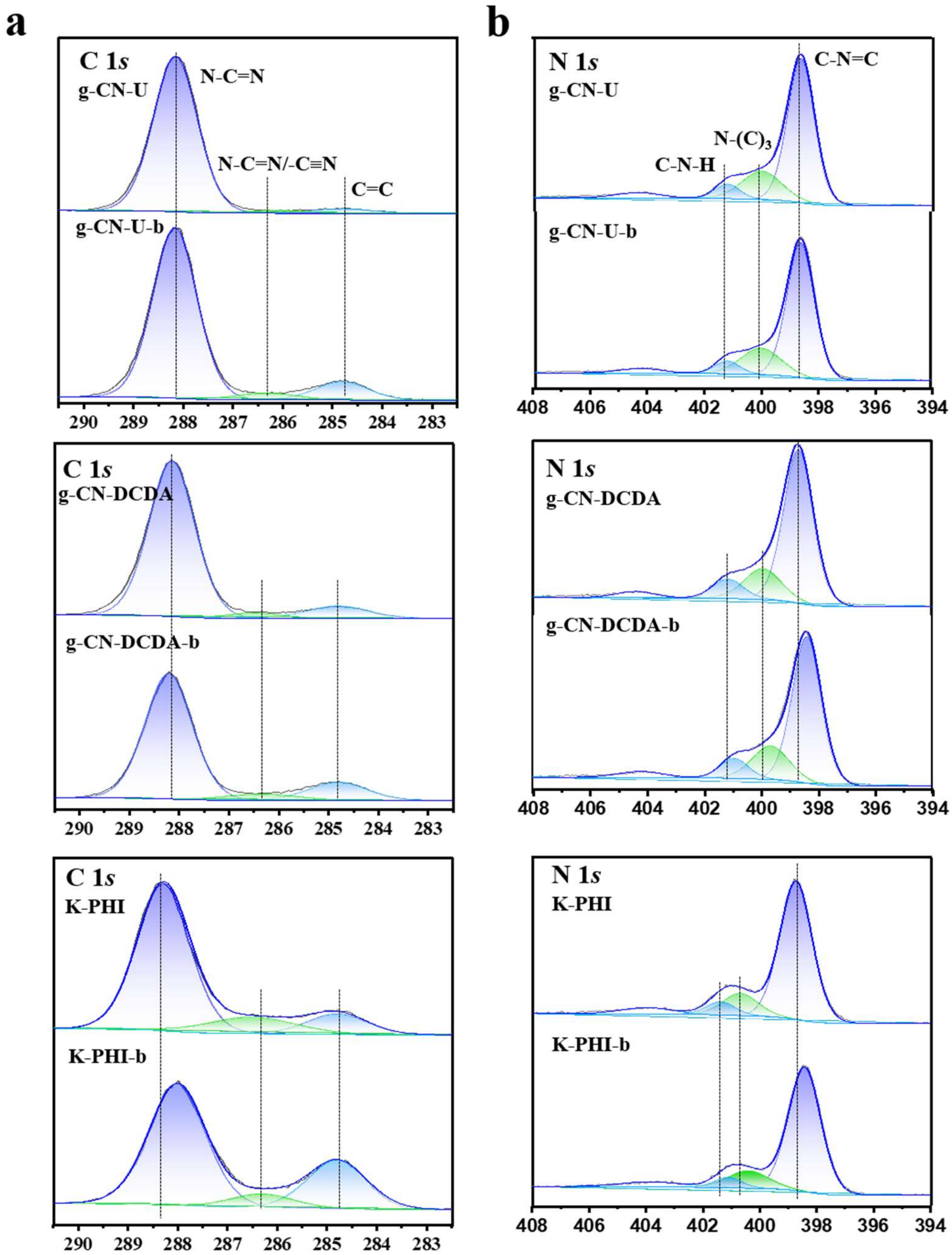

Supplementary Figure 25. High-resolution XPS spectra of the catalysts before and after base treatment. High-resolution XPS spectra of (a) C 1*s* and (b) N 1*s* of g-CN-DCDA, g-CN-U, K-PHI, g-CN-DCDA-b, g-CN-U-b and K-PHI-b.

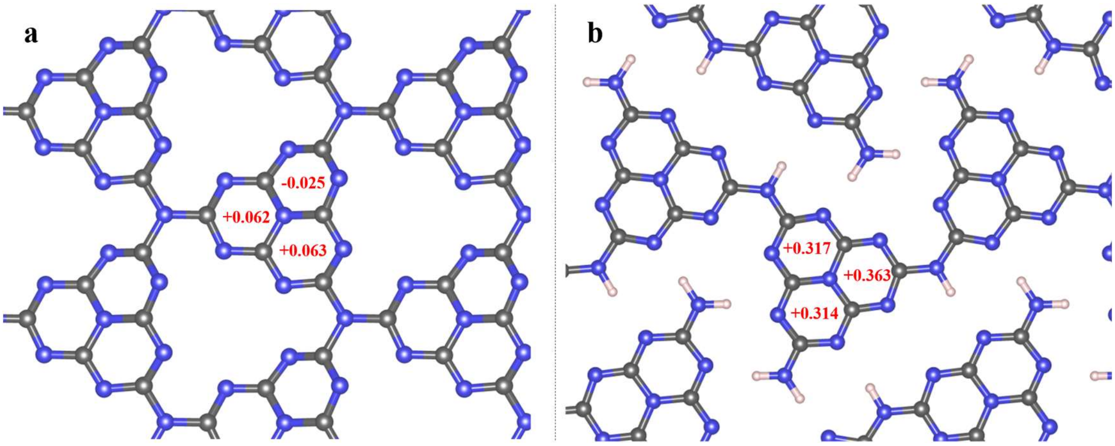

Supplementary Figure 26. The calculated electron distribution of the heptazine units in carbon nitrides. (a) g-C_3N_4-type structure. (b) melon-type structure.

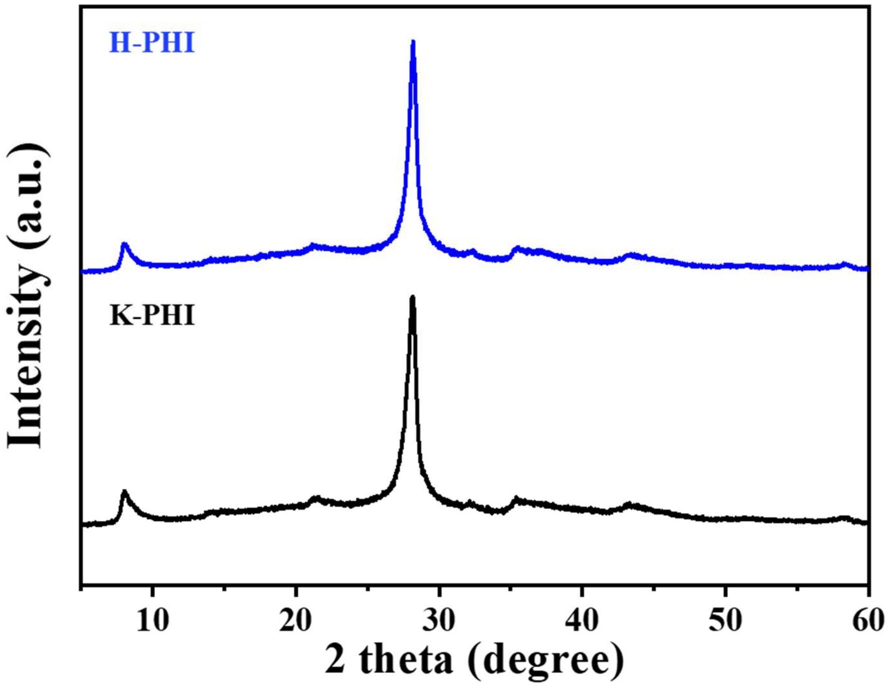

Supplementary Figure 27. Catalysts PXRD characterization. PXRD results of K-PHI and H-PHI.

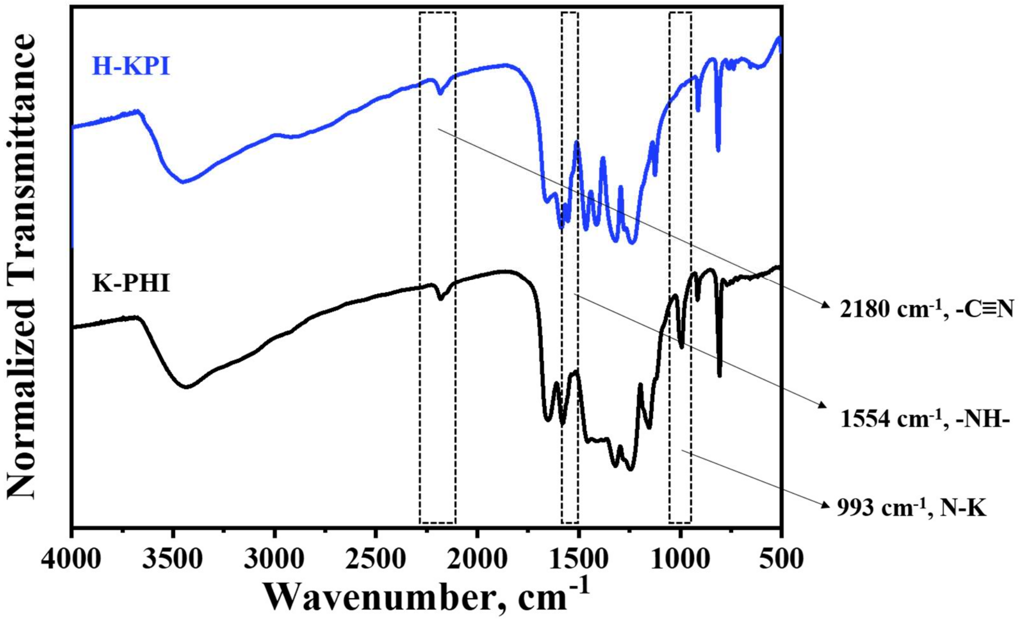

Supplementary Figure 28. FT-IR spectra of catalysts. FT-IR spectra of K-PHI and H-PHI.

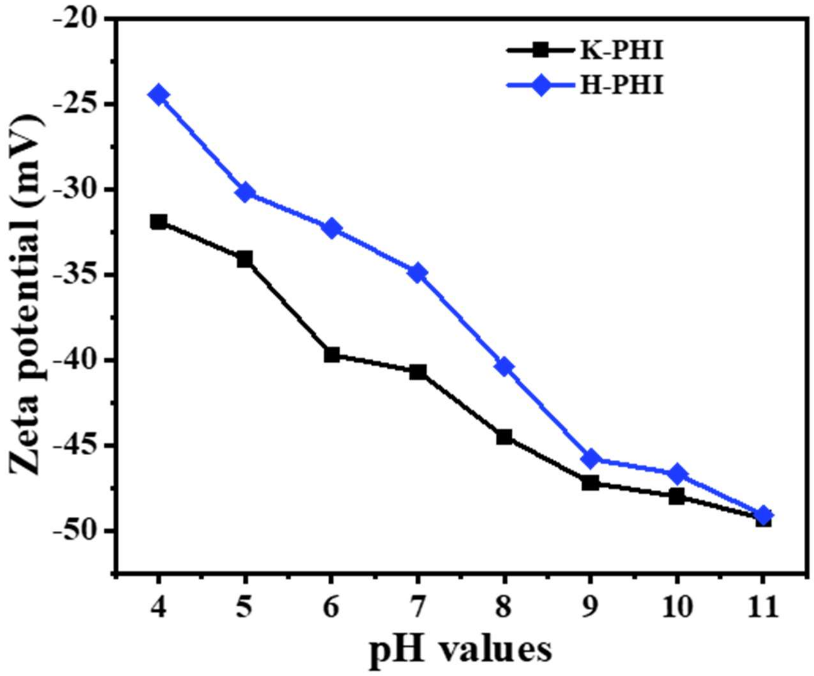

Supplementary Figure 29. Zeta potential of the catalysts. Zeta potential of K-PHI and H-PHI samples were examined under various pH value conditions.

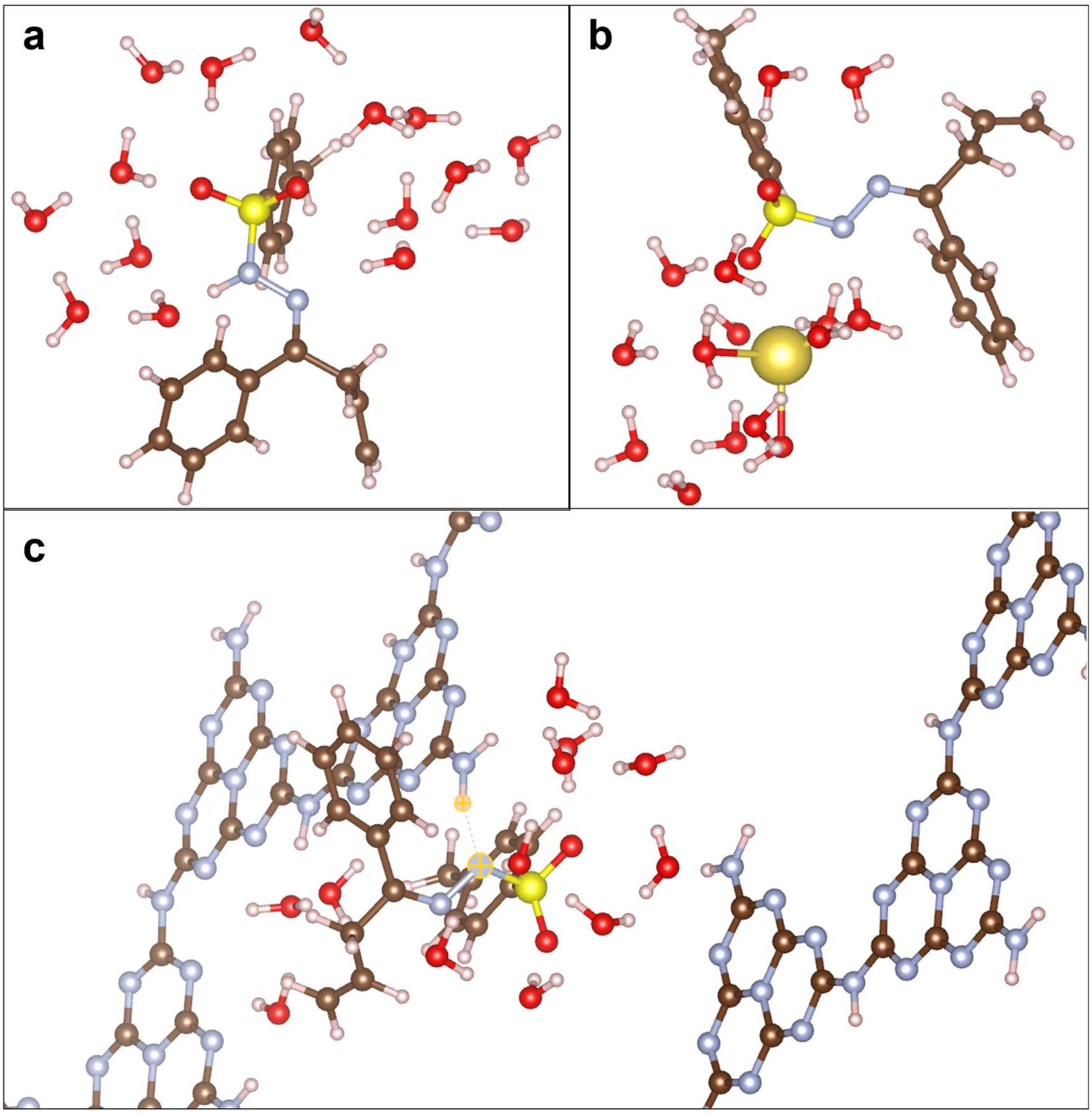

Supplementary Figure 30. Optimized configurations of the three substrate states. (a) free β,γ-unsaturated hydrazone molecule, (b) interacted with sodium hydroxide, (c) adsorption of the deprotonated hydrazone on the g-CN-U surface.

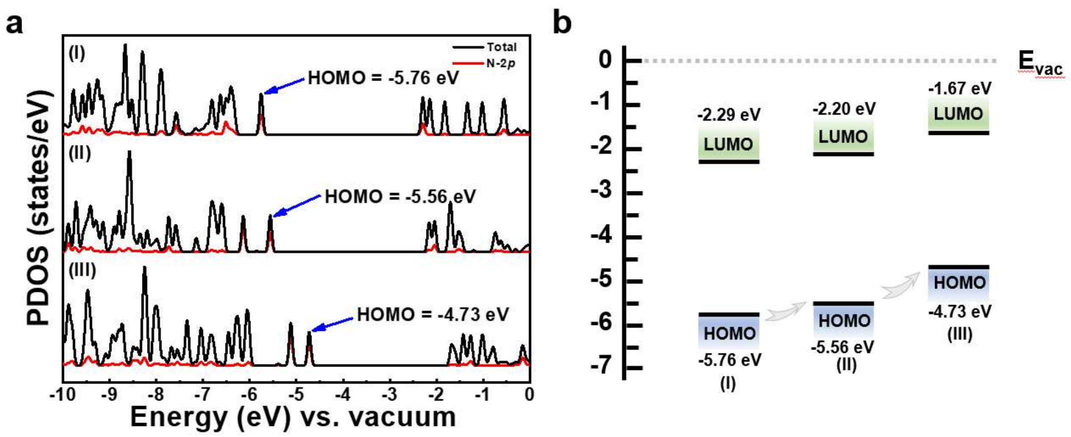

Supplementary Figure 31. The calculated energy levels of three substrate states. (a) DOS of hydrazone various states, (b) Energy levels of hydrazone various states. (I) free hydrazone. (II) deprotonated hydrazone. (III) deprotonated hydrazone adsorbed to the g-CN-U surface.

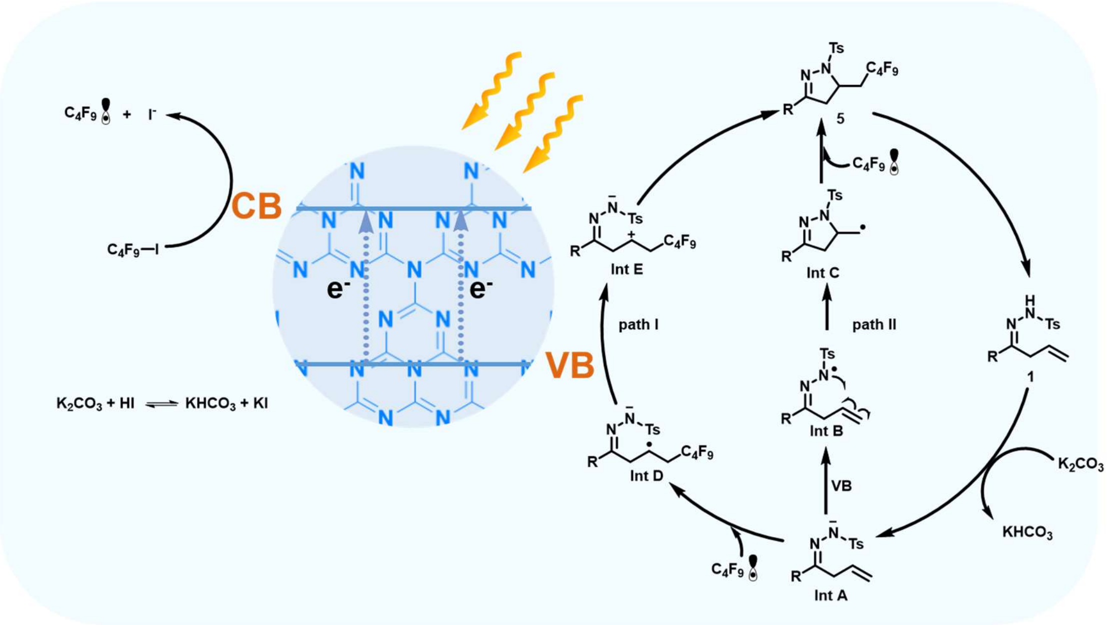

Supplementary Figure 32. Proposed reaction pathway. Plausible mechanism for the formation of **5a-5c**.

Based on the literature[30-32] and our experimental results, this reaction possibly proceeds through the following pathways. Deprotonation of hydrazone first occured to generate the anionic intermediate **Int A**. The subsequent single-electron oxidation of **Int A** by the light-excited valence band (VB) hole of the photocatalyst took place to deliver the nitrogen-centered radical **Int B**. A 5-exo-trig cyclization of **Int B** furnished the carbon-centered radical **Int C**. Cross-coupling of **Int C** with the reactive perfluoroalkyl radical, which were generated through the single-electron reduction of C_4F_9I by the conduction band (CB) electron of the photocatalyst, finally proceeded to afford the perfluoroalkylation product **5**. Alternatively, the reaction may be initiated by the addition of the electrophilic perfluoroalkyl radical on the electron-rich C-C double bond of **Int A** to create the carbon-centered radical intermediate **Int D**. The SET oxidation of **Int D** by the VB holes of the photocatalyst[33] and intramolecular cyclization of **Int E** finally afforded the desired product **5**.

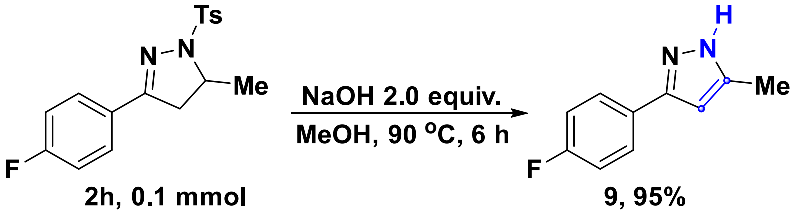

Supplementary Figure 33. Procedure for the synthesis of pyridazine 9. A mixture of **2h** (33.2 mg, 0.1 mmol) and NaOH (8.0 mg, 2.0 equiv.) in methanol (2 mL) was heated to 90 °C and stirred for about 6 hours until completion (monitored by TLC analysis). The crude product was purified by flash chromatography on silica gel (petroleum ether/ethyl acetate 5:1~3:1) directly to give the desired product **9** in 95% (16.7 mg) isolated yield as a white solid.

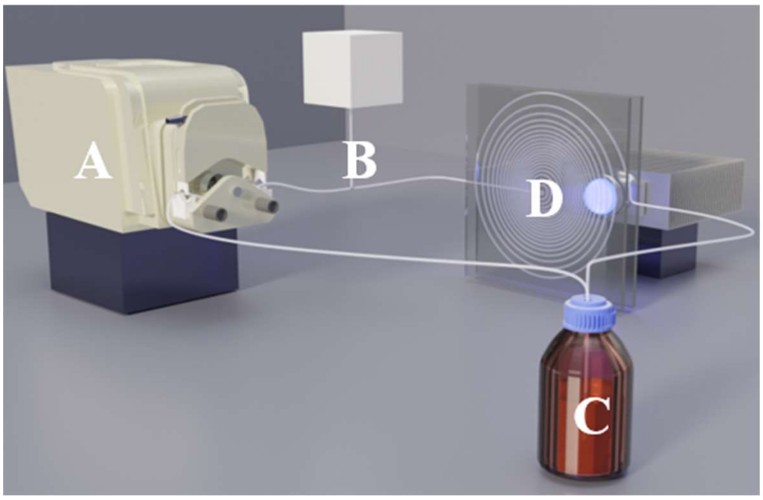

Supplementary Figure 34. Flow photoreactor designed in this work.

A: The power assisting device includes a peristaltic pump (30 rpm, BT100-2J).

B: Tee-junction (one-way join the inert gas for filling shielding gas, the others connect **C** and **D**).

C: collection device includes a gather bottle and a cold trap.

D: Photoreactor includes φ3 PTFE (1.58 mm inner diameter) tube as flow reactor and 50 W blue LED light resource.

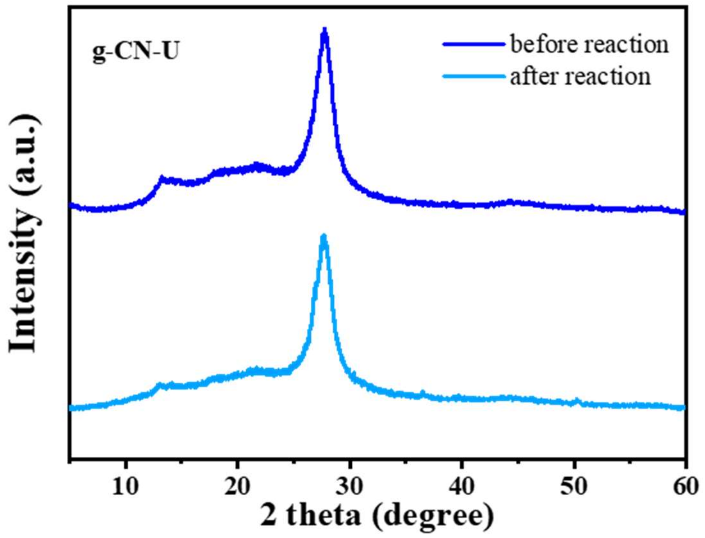

Supplementary Figure 35. Catalysts PXRD characterization. The PXRD spectra of g-CN-U before and after the catalytic reaction.

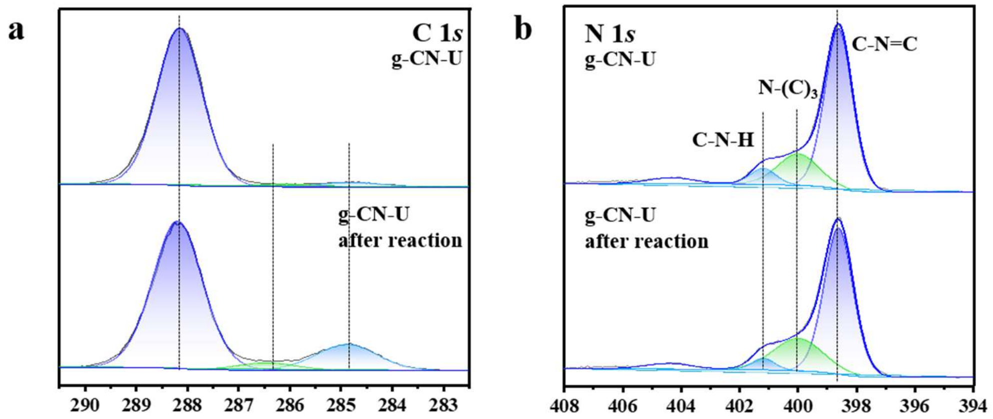

Supplementary Figure 36. XPS spectra of the catalysts. XPS spectra of g-CN before and after the catalytic reaction. High-resolution XPS spectra of (a) C 1*s*, (b) N 1*s* of the catalysts.

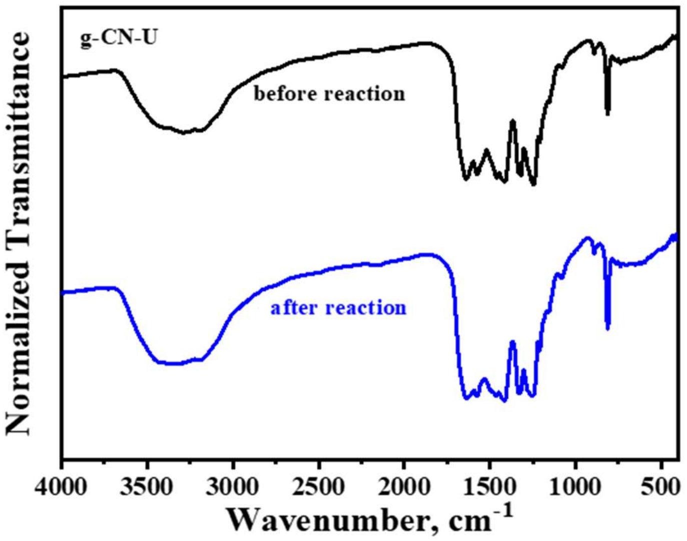

Supplementary Figure 37. FT-IR spectra of the catalysts. FT-IR spectra of g-CN-U before and after the catalytic reaction.

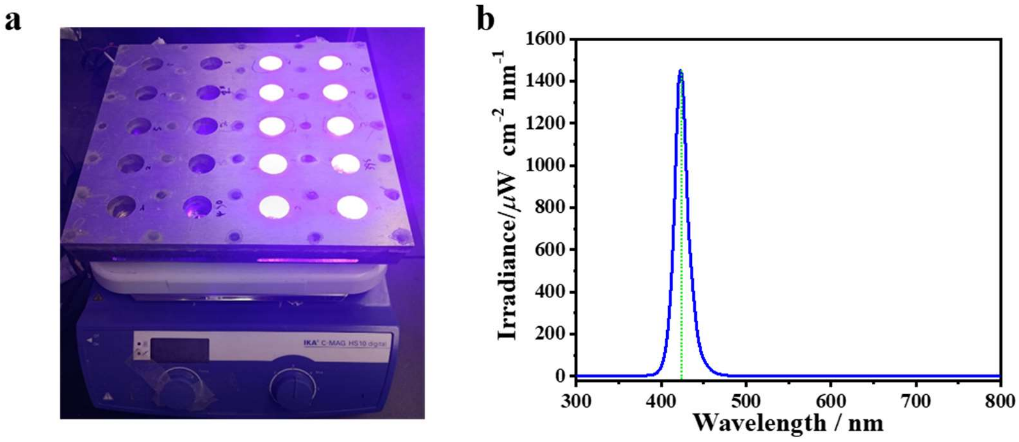

Supplementary Figure 38. Photoreactor configuration. (a) a 20-well plate photoreactor, (b) The optical spectrum of a 420 nm LED (Reactions were irradiated using a simple photoreactor consisting of Eaglerise ELP8X3LS 3W blue LEDs ($\lambda = 420$ nm), which was connected to HAAKE-FK cyclic water cooling system at 25 ºC.)

.

General Procedure for the Photocatalytic Reaction

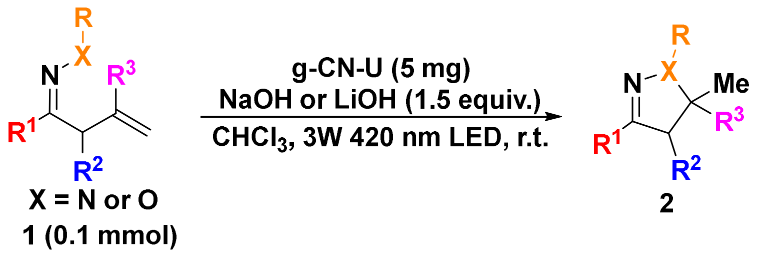

A solution of β,γ-Unsaturated hydrazone **1** (0.1 mmol, 1 equiv.), carbon nitride g-CN-U (5 mg), anhydrous sodium hydroxide or lithium hydroxide (1.5 equiv.) in 2 mL dry chloroform were added into a Schlenk tube equipped with a stirring bar. The Schlenk tube was purged with nitrogen three times. After degassing by the freeze-pump-thaw process for three cycles, the reaction mixture was irradiated with a 3 W 420 nm LED at 25 ºC for 8 ~ 12 hours, after completion of the reaction monitored by TLC, the crude product was purified by short flash chromatography on silica gel (petroleum ether/ethyl acetate 12:1~8:1) to give the desired product. The product was analyzed by ^{1}H NMR and ^{13}C NMR.

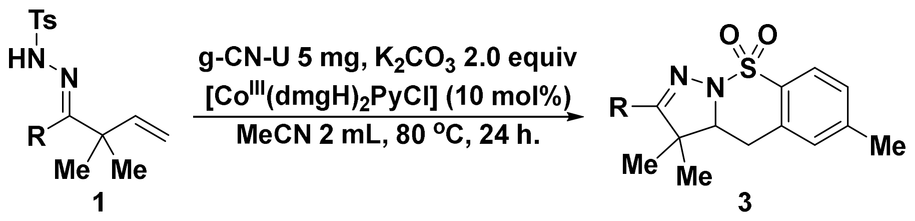

1 (0.1 mmol), g-CN-U (5.0 mg), K_2CO_3 (27.6 mg, 0.2 mmol), [Co^{III}(dmgH)$_2$PyCl] (4.0 mg, 0.01 mmol) were dissolved in MeCN (2.0 mL). Then, the resulting mixture was degassed via the 'freeze-pump-thaw' procedure (3 times) under argon atmosphere. After that, the solution was stirred at 50 W blue LEDs (420 nm) at 80 ºC for about 24 h until completion (monitored by TLC analysis). The crude product was purified by flash chromatography on silica gel (petroleum ether/ethyl acetate 10:1~3:1) to give the desired product **3** as a white solid.

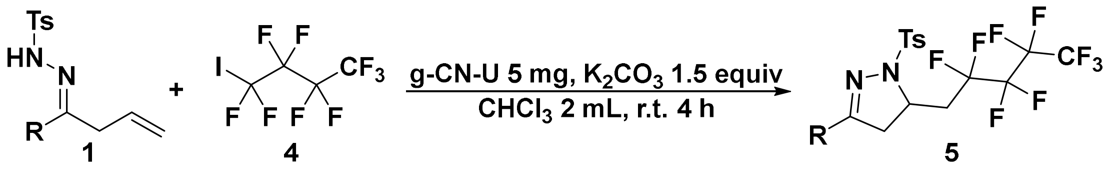

β,γ-Unsaturated hydrazone **1** (0.1 mmol), perfluorobutyl iodide (34 *u*L, 0.2 mmol), g-CN-U (5.0 mg), K_2CO_3 (20.7 mg, 0.15 mmol) were dissolved in chloroform (2.0 mL). The resulting mixture was then degassed via the 'freeze-pump-thaw' procedure (3 times) under argon atmosphere. After that, the solution was stirred at 3 W blue LEDs (420 nm) at room temperature until the reaction was completed (monitored by TLC analysis). The crude product was purified by column chromatography on silica gel (petroleum ether/ethyl acetate 15:1~8:1) to give the desired product **5** as a brown oil.

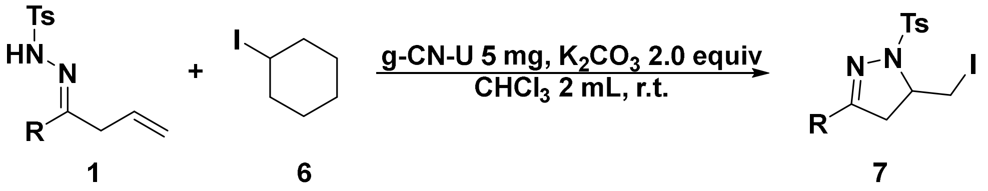

1 (0.1 mmol), iodocyclohexane (26 *u*L, 0.2 mmol), g-CN-U (5.0 mg), K_2CO_3 (27.6 mg, 0.2 mmol) were dissolved in chloroform (2.0 mL). Then, the resulting mixture was degassed via the 'freeze-pump-thaw' procedure (3 times) under argon atmosphere. After that, the solution was stirred at 3 W blue LEDs (420 nm) at room temperature until completion (monitored by TLC analysis). The crude product was purified by column chromatography on silica gel (petroleum ether/ethyl acetate 15:1~8:1) to give the desired product **7** as a white solid.

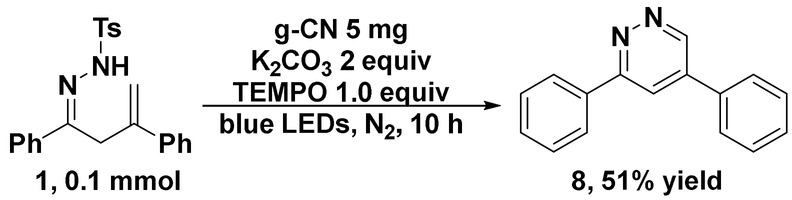

1 (39.0 mg, 0.1 mmol), TEMPO (15.6 mg, 0.1 mmol), g-CN-U (5.0 mg), K_2CO_3 (27.6 mg, 0.2 mmol) were dissolved in chloroform (2.0 mL). Then, the resulting mixture was degassed via the 'freeze-pump-thaw' procedure (3 times) under argon atmosphere. After that, the solution was stirred at 50 W blue LEDs (420 nm) at room temperature for about 10 h until the reaction was completed (monitored by TLC analysis). The crude product was purified by flash chromatography on silica gel (petroleum ether/ethyl acetate 10:1~3:1) to give the desired product **8** in 51% (11.8 mg) isolated yield as a white solid.

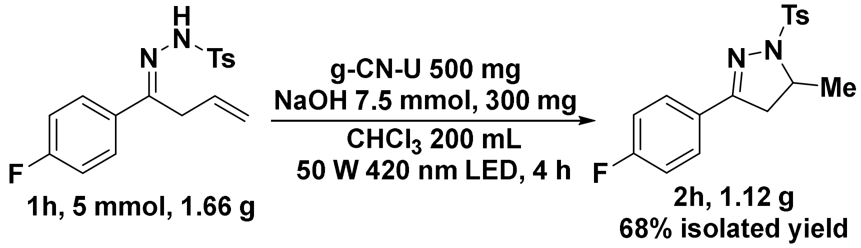

The g-CN-U sample was grounded and filtrated through a 300 ~ 400 mesh sieve to ensure the size uniformity of the photocatalyst. The substrate, catalyst and solvent were all dried and stored in the glove box before the reaction. At first, a solution of β,γ-unsaturated hydrazone **1h** (5 mmol), g-CN-U (500 mg), anhydrous sodium hydroxide (300 mg, 1.5 equiv.) in 200 mL chloroform were added into a 250 mL brown bottle as the collecting device. Meanwhile, the flow reactor (Fig. 6) was purged with nitrogen continuously through the tee-junction with a rate of 100 ~ 200 mL/min for 15 min. The collecting device was then connected with the flow reactor and the nitrogen purging was stopped. The reaction mixture was irradiated with a 50 W 420 nm LED at room temperature for 4 hours, and the crude product was purified by flash chromatography on silica gel (petroleum ether/ethyl acetate 10:1) to give the desired product in 68% isolated yield (**2h**, 1.12 g).

Characterization of Products

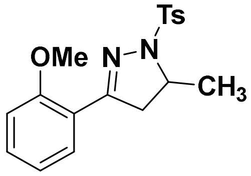

3-(2-methoxyphenyl)-5-methyl-1-tosyl-4,5-dihydro-1*H*-pyrazole (2a). Yield of **2a**: 27.5 mg in 80% isolated yield, as a yellow oil. ^{1}H NMR (600 MHz, $CDCl_3$) δ (ppm) = 7.82 (d, *J* = 8.4 Hz, 2H), 7.79 (dd, *J* = 7.8, 1.8 Hz, 1H), 7.36-7.33 (m, 1H), 7.29 (d, *J* = 7.8 Hz, 2H), 6.97-6.94 (m, 1H), 6.87 (dd, *J* = 8.4, 1.2 Hz, 1H), 3.82-3.78 (m, 1H), 3.78 (s, 3H), 3.25 (dd, *J* = 17.4, 10.2 Hz, 1H), 2.94 (dd, *J* = 17.4, 10.2 Hz, 1H), 2.40 (s, 3H), 1.61 (d, *J* = 6.0 Hz, 3H).

^{13}C NMR (150 MHz, $CDCl_3$) δ (ppm) δ = 157.96, 157.47, 143.90, 132.34, 131.60, 129.46, 129.33, 128.71, 120.70, 120.24, 111.34, 58.34, 55.32, 44.84, 21.55.

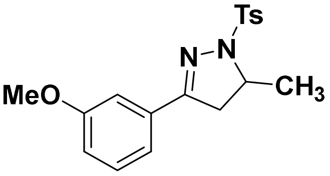

3-(3-methoxyphenyl)-5-methyl-1-tosyl-4,5-dihydro-1*H*-pyrazole (2b). Yield of **2b**: 27.1 mg in 79% isolated yield, as a yellow oil. ^{1}H NMR (600 MHz, $CDCl_3$) δ (ppm) = 7.80 (d, *J* = 8.3 Hz, 2H), 7.28 (dd, *J* = 8.0, 4.8 Hz, 3H), 7.25-7.23 (m, 1H), 7.17-7.14 (m, 1H), 6.96-6.93 (m, 1H), 3.92-3.86 (m, 1H), 3.84 (s, 3H), 3.16 (dd, *J* = 16.9, 10.6 Hz, 1H), 2.75 (dd, *J* = 16.9, 9.8 Hz, 1H), 2.38 (s, 3H), 1.63 (d, *J* = 6.2 Hz, 3H).

^{13}C NMR (150 MHz, $CDCl_3$) δ (ppm) δ = 159.64, 157.12, 144.08, 132.31, 132.27, 129.55, 129.44, 128.62, 119.49, 116.52, 111.61, 58.22, 55.43, 41.61, 21.86, 21.56.

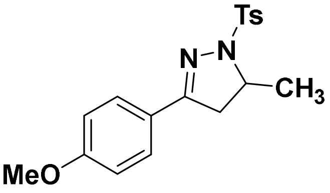

3-(4-methoxyphenyl)-5-methyl-1-tosyl-4,5-dihydro-1*H*-pyrazole (2c). Yield of **2c**: 24.7 mg in 72% isolated yield, as a yellow oil. ^{1}H NMR (600 MHz, $CDCl_3$) δ (ppm) = 7.79 (d, *J* = 8.4 Hz, 2H), 7.59 (d, *J* = 8.4 Hz, 2H), 7.26 (d, *J* = 8.4 Hz, 2H), 6.88 (d, *J* = 9.0 Hz, 2H), 3.89-3.84 (m, 1H), 3.83 (s, 3H), 3.13 (dd, *J* = 16.8, 10.8 Hz, 1H), 2.73 (dd, *J* = 16.8, 9.6 Hz, 1H), 2.38 (s, 3H), 1.62 (d, *J* = 6.0 Hz, 3H).

^{13}C NMR (150 MHz, $CDCl_3$) δ (ppm) δ = 161.38, 157.01, 143.96, 132.18, 129.38, 128.66, 128.43, 123.61, 113.93, 58.00, 55.34, 41.60, 21.83, 21.54.

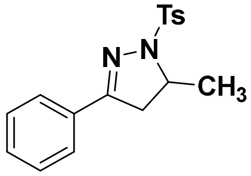

5-methyl-3-phenyl-1-tosyl-4,5-dihydro-1*H*-pyrazole (2d). Yield of **2d**: 23.5 mg in 75% isolated yield, as a colorless oil. ^{1}H NMR (600 MHz, $CDCl_3$) δ (ppm) 7.80 (d, *J* = 8.4 Hz, 2H), 7.65 (dd, *J* = 7.8, 1.2 Hz, 2H), 7.41-7.36 (m, 3H), 7.27 (d, *J* = 7.8 Hz, 2H), 3.93-3.86 (m, 1H), 3.18 (dd, *J* = 16.8, 10.8 Hz, 1H), 2.77 (dd, *J* = 16.8, 9.6 Hz, 1H), 2.38 (s, 3H), 1.64 (d, *J* = 6.0 Hz, 3H).

^{13}C NMR (150 MHz, $CDCl_3$) δ (ppm) δ = 157.18, 144.06, 132.27, 130.97, 130.42, 129.43, 128.63, 128.55, 126.80, 58.20, 41.51, 21.82, 21.54.

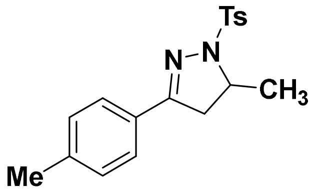

5-methyl-3-(p-tolyl)-1-tosyl-4,5-dihydro-1*H*-pyrazole (2e). Yield of **2e**: 26.5 mg in 81% isolated yield, as a colorless oil. ^{1}H NMR (600 MHz, $CDCl_3$) δ (ppm) = 7.80 (d, *J* = 9.6 Hz, 2H), 7.54 (d, *J* = 8.4 Hz, 2H), 7.28-7.25 (m, 4H), 7.17 (d, *J* = 7.8 Hz, 2H), 3.91-3.81 (m, 1H), 3.15 (dd, *J* = 16.8, 10.2 Hz, 1H), 2.75 (dd, *J* = 16.8, 9.6 Hz, 1H), 2.37 (s, 3H), 2.36 (s, 3H), 1.63 (d, *J* = 6.0 Hz, 3H).

^{13}C NMR (150 MHz, $CDCl_3$) δ (ppm) δ = 157.26, 143.97, 140.79, 132.25, 129.39, 129.25, 128.65, 128.23, 126.78, 58.09, 41.57, 21.85, 21.55, 21.44.

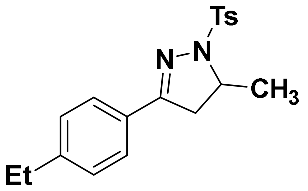

3-(4-ethylphenyl)-5-methyl-1-tosyl-4,5-dihydro-1*H*-pyrazole (2f). Yield of **2f**: 29.4 mg in 86% isolated yield, as a colorless oil. ^{1}H NMR (600 MHz, $CDCl_3$) δ (ppm) = 7.80 (d, *J* = 8.4 Hz, 2H), 7.57 (d, *J* = 8.4 Hz, 2H), 7.26 (d, *J* = 8.4 Hz, 2H), 7.20 (d, *J* = 8.4 Hz, 2H), 3.89-3.83 (m, 1H), 3.16 (dd, *J* = 16.8, 10.8 Hz, 1H), 2.75 (dd, *J* = 16.8, 9.6 Hz, 1H), 2.66 (q, *J* = 7.8 Hz, 2H), 2.38 (s, 3H), 1.63 (d, *J* = 6.0 Hz, 3H), 1.23 (t, *J* = 7.8 Hz, 3H).

^{13}C NMR (150 MHz, $CDCl_3$) δ (ppm) δ = 157.28, 147.09, 143.98, 132.24, 129.41, 128.64, 128.45, 128.08, 126.89, 58.09, 41.58, 28.77, 21.85, 21.54, 15.31.

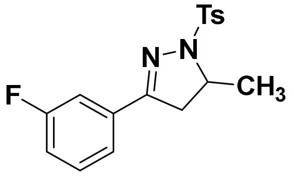

3-(3-fluorophenyl)-5-methyl-1-tosyl-4,5-dihydro-1*H*-pyrazole (2g). Yield of **2g**: 22.2 mg in 67% isolated yield,% as a yellow oil. ^{1}H NMR (600 MHz, $CDCl_3$) δ (ppm) = 7.80 (d, *J* = 7.8 Hz, 2H), 7.41-7.36 (m, 2H), 7.34-7.32 (m, 1H), 7.29 (d, *J* = 8.4 Hz, 2H), 7.11-7.07 (m, 1H), 4.00-3.88 (m, 1H), 3.17 (dd, *J* = 16.8, 10.2 Hz, 1H), 2.75 (dd, *J* = 16.8, 9.6 Hz, 1H), 2.39 (s, 3H), 1.64 (d, *J* = 6.0 Hz, 3H).

^{13}C NMR (150 MHz, $CDCl_3$) δ (ppm) δ = 162.71 (d, *J* = 246.6 Hz), 155.97, 144.22, 133.12, 132.30, 130.17 (d, *J* = 7.8 Hz), 129.50, 128.60, 122.55, 117.36 (d, *J* = 21.6 Hz), 113.51 (d, *J* = 22.8 Hz), 58.42, 41.45, 21.78, 21.58.

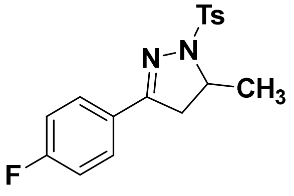

3-(4-fluorophenyl)-5-methyl-1-tosyl-4,5-dihydro-1*H*-pyrazole (2h). Yield of **2h**: 23.2 mg in 70% isolated yield, as a yellow oil. ^{1}H NMR (600 MHz, $CDCl_3$) δ (ppm) = 7.80 (d, *J* = 8.4 Hz, 2H), 7.64 (dd, *J* = 9.0, 5.4 Hz, 2H), 7.28 (d, *J* = 7.8 Hz, 2H), 7.06 (t, *J* = 8.4 Hz, 2H), 3.96-3.86 (m, 1H), 3.16 (dd, *J* = 16.8, 10.2 Hz, 1H), 2.75 (dd, *J* = 16.8, 9.6 Hz, 1H), 2.39 (s, 3H), 1.64 (d, *J* = 6.0 Hz, 3H).
^{13}C NMR (150 MHz, $CDCl_3$) δ (ppm) δ = 164.01 (d, *J* = 251.8 Hz), 156.11, 144.13, 132.28, 129.45, 128.82, 128.76, 128.63, 127.27 (d, *J* = 3.0 Hz), 115.73 (d, *J* = 21.6 Hz), 58.28, 41.57, 21.80, 21.57.

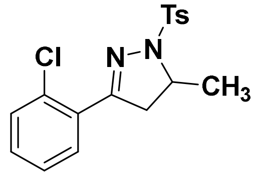

3-(2-chlorophenyl)-5-methyl-1-tosyl-4,5-dihydro-1*H*-pyrazole (2i). Yield of **2i**: 25 mg in 72% isolated yield, as a white solid. ^{1}H NMR (600 MHz, $CDCl_3$) δ (ppm) = 7.83 (d, *J* = 8.4 Hz, 2H), 7.59 (dd, *J* = 7.8, 1.8 Hz, 1H), 7.35 (dd, *J* = 7.8, 1.2 Hz, 1H), 7.33-7.29 (m, 3H), 7.28-7.25 (m, 1H), 3.94-3.87 (m, 1H), 3.30 (dd, *J* = 16.8, 10.2 Hz, 1H), 2.99 (dd, *J* = 16.8, 10.2 Hz, 1H), 2.42 (s, 3H), 1.64 (d, *J* = 6.0 Hz, 3H).
^{13}C NMR (150 MHz, $CDCl_3$) δ (ppm) δ = 157.41, 144.25, 136.95, 132.76, 132.25, 130.95, 130.50, 130.48, 129.46, 128.73, 126.85, 58.90, 44.59, 21.59, 21.34.

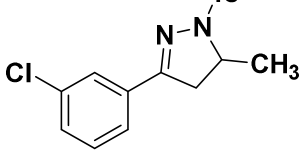

3-(3-chlorophenyl)-5-methyl-1-tosyl-4,5-dihydro-1*H*-pyrazole (2j). Yield of **2j**: 28.8 mg in 83% isolated yield, as a white solid. ^{1}H NMR (600 MHz, $CDCl_3$) δ (ppm) = 7.80 (d, *J* = 8.4 Hz, 2H), 7.63 (t, *J* = 1.8 Hz, 1H), 7.56-7.50 (m, 1H), 7.37-7.35 (m, 1H), 7.32-7.28 (m, 3H), 3.96-3.90 (m, 1H), 3.17 (dd, *J* = 16.8, 10.8 Hz, 1H), 2.75 (dd, *J* = 16.8, 9.6 Hz, 1H), 2.39 (s, 3H), 1.64 (d, *J* = 6.0 Hz, 3H).

^{13}C NMR (150 MHz, $CDCl_3$) δ (ppm) δ = 155.80, 144.24, 134.69, 132.74, 132.27, 130.34, 129.84, 129.52, 128.58, 126.75, 124.84, 58.37, 41.37, 21.78, 21.57.

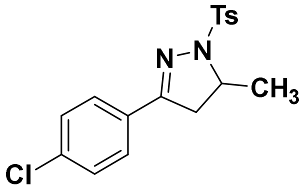

3-(4-chlorophenyl)-5-methyl-1-tosyl-4,5-dihydro-1*H*-pyrazole (2k). Yield of **2k**: 28.4 mg in 82% isolated yield, as a white solid. ^{1}H NMR (600 MHz, $CDCl_3$) δ (ppm) = 7.79 (d, *J* = 8.4 Hz, 2H), 7.57 (d, *J* = 8.4 Hz, 2H), 7.34 (d, *J* = 8.4 Hz, 2H), 7.28 (d, *J* = 8.4 Hz, 2H), 3.95-3.88 (m, 1H), 3.16 (dd, *J* = 16.8, 10.2 Hz, 1H), 2.74 (dd, *J* = 16.8, 10.2 Hz, 1H), 2.39 (s, 3H), 1.64 (d, *J* = 6.0 Hz, 3H).

^{13}C NMR (150 MHz, $CDCl_3$) δ (ppm) δ = 156.04, 144.19, 136.48, 132.28, 129.48, 128.86, 128.61, 128.01, 58.37, 41.40, 21.79, 21.57

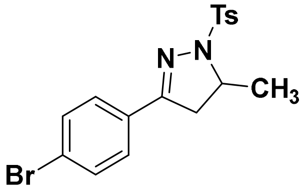

3-(4-bromophenyl)-5-methyl-1-tosyl-4,5-dihydro-1*H*-pyrazole (2l). Yield of **2l**: 28.6 mg in 73% isolated yield, as a white solid. ^{1}H NMR (600 MHz, $CDCl_3$) δ (ppm) = 7.79 (d, *J* = 8.4 Hz, 2H), 7.51-7.50 (m, 4H), 7.30-7.27 (m, 2H), 4.00-3.86 (m, 1H), 3.16 (dd, *J* = 16.8, 10.8 Hz, 1H), 2.74 (dd, *J* = 16.8, 9.6 Hz, 1H), 2.39 (s, 3H), 1.64 (d, *J* = 6.0 Hz, 3H).

^{13}C NMR (150 MHz, $CDCl_3$) δ (ppm) δ = 156.12, 144.20, 132.24, 131.81, 129.89, 129.48, 128.59, 128.21, 124.84, 58.38, 41.33, 21.78, 21.57.

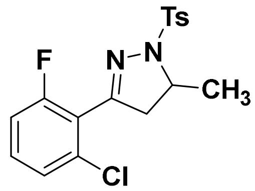

3-(2-chloro-6-fluorophenyl)-5-methyl-1-tosyl-4,5-dihydro-1*H*-pyrazole (2m). Yield of **2m**: 30.0 mg in 82% isolated yield, as a white solid. ^{1}H NMR (600 MHz,

$CDCl_3$) δ (ppm) = 7.82 (d, *J* = 8.4 Hz, 2H), 7.32 (d, *J* = 8.4 Hz, 2H), 7.30-7.27 (m, 1H), 7.19-7.18 (m, 1H), 7.03-7.00 (m, 1H), 3.99-3.93 (m, 1H), 3.04 (dd, *J* = 17.4, 10.2 Hz, 1H), 2.87 (dd, *J* = 17.4, 9.6 Hz, 1H), 2.44 (s, 3H), 1.64 (d, *J* = 6.0 Hz, 3H).

^{13}C NMR (150 MHz, $CDCl_3$) δ (ppm) δ = 160.60 (d, *J* = 252.6 Hz), 159.76, 152.42, 144.21, 134.16, 132.33, 131.15 (d, *J* = 10.2 Hz), 129.45, 128.82, 125.53 (d, *J* = 3.6 Hz), 120.10 (d, *J* = 18.0 Hz), 114.37 (d, *J* = 22.4 Hz), 58.22, 44.96, 21.60, 21.52.

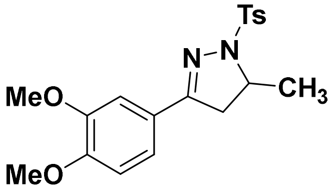

3-(3,4-dimethoxyphenyl)-5-methyl-1-tosyl-4,5-dihydro-1*H*-pyrazole (2n). Yield of **2n**: 28.8 mg in 77% isolated yield, as a yellow solid. ^{1}H NMR (600 MHz, $CDCl_3$) δ (ppm) = 7.80 (d, *J* = 8.4 Hz, 2H), 7.38 (d, *J* = 2.4 Hz, 1H), 7.26 (d, *J* = 8.4 Hz, 2H), 7.01 (dd, *J* = 8.4, 2.4 Hz, 1H), 6.81 (d, *J* = 8.4 Hz, 1H), 3.94 (s, 3H), 3.90 (s, 3H), 3.87-3.84 (m, 1H), 3.15 (dd, *J* = 16.8, 10.4 Hz, 1H), 2.74 (dd, *J* = 16.8, 9.6Hz, 1H), 2.38 (s, 3H), 1.62 (d, *J* = 6.0 Hz, 3H).

^{13}C NMR (150 MHz, $CDCl_3$) δ (ppm) δ = 157.18, 151.20, 149.06, 144.02, 132.13, 129.39, 128.63, 123.89, 120.62, 110.31, 108.85, 58.07, 56.09, 55.91, 41.57, 21.89, 21.54.

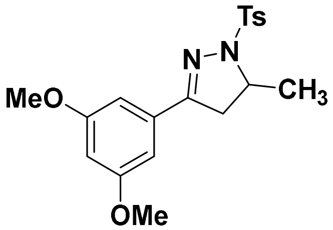

3-(3,5-dimethoxyphenyl)-5-methyl-1-tosyl-4,5-dihydro-1*H*-pyrazole (2o). Yield of **2o**: 30 mg in 80% isolated yield, as a yellow solid. ^{1}H NMR (600 MHz, $CDCl_3$) δ (ppm) = 7.79 (d, *J* = 8.4 Hz, 2H), 7.28 (s, 2H), 6.78 (d, *J* = 2.4 Hz, 2H), 6.49 (t, *J* = 2.4 Hz, 1H), 3.91-3.86 (m, 1H), 3.81 (s, 6H), 3.14 (dd, *J* = 16.8, 10.8 Hz, 1H), 2.72 (dd, *J* = 16.8, 9.6 Hz, 1H), 2.39 (s, 3H), 1.62 (d, *J* = 6.0 Hz, 3H).

^{13}C NMR (150 MHz, $CDCl_3$) δ (ppm) δ = 160.74, 157.20, 144.12, 132.81, 132.18, 129.45, 128.60, 104.88, 102.54, 58.23, 55.54, 41.62, 21.86, 21.56.

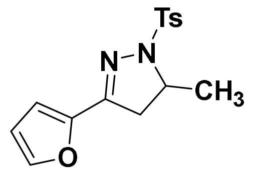

3-(furan-2-yl)-5-methyl-1-tosyl-4,5-dihydro-1*H*-pyrazole (2p). Yield of **2p**: 10.0 mg in 33% isolated yield, as a yellow oil. ^{1}H NMR (600 MHz, $CDCl_3$) δ (ppm) = 7.80 (d, *J* = 8.4 Hz, 2H), 7.47 (dd, *J* = 1.8, 0.6 Hz, 1H), 7.28 (d, *J* = 8.4 Hz, 2H), 6.76 (d, *J* = 3.6 Hz, 1H), 6.46 (dd, *J* = 3.6, 1.8 Hz, 1H), 3.90-3.83 (m, 1H), 3.14 (dd, *J* = 17.4, 10.4 Hz, 1H), 2.73 (dd, *J* = 17.4, 9.6 Hz, 1H), 2.40 (s, 3H), 1.61 (d, *J* = 6.0 Hz, 3H).

^{13}C NMR (150 MHz, $CDCl_3$) δ (ppm) δ = 148.96, 146.70, 144.55, 132.33, 129.48, 128.69, 112.17, 111.90, 57.68, 41.41, 29.68, 21.67.

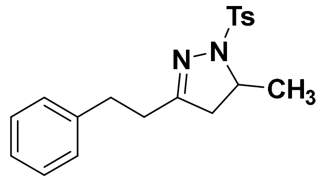

5-methyl-3-phenethyl-1-tosyl-4,5-dihydro-1*H*-pyrazole (2q). Yield of **2q**: 17.1 mg in 50% isolated yield, as a colorless oil. ^{1}H NMR (600 MHz, $CDCl_3$) δ (ppm) = 7.73 (d, *J* = 8.4 Hz, 2H), 7.29 (d, *J* = 7.8 Hz, 2H), 7.23 (t, *J* = 7.2 Hz, 2H), 7.19-7.17 (m, 1H), 7.44 (d, *J* = 7.2 Hz, 2H), 3.70-3.63 (m, 1H), 2.87-2.82 (m, 1H), 2.78-2.73 (m, 1H), 2.60-2.50 (m, 3H), 2.43 (s, 3H), 2.29 (dd, *J* = 17.4, 9.6 Hz, 1H), 1.49 (d, *J* = 6.0 Hz, 3H).

^{13}C NMR (150 MHz, $CDCl_3$) δ (ppm) δ = 161.96, 143.94, 140.40, 132.09, 129.33, 128.74, 128.49, 128.15, 126.27, 57.40, 44.37, 32.61, 31.70, 21.68, 21.58.

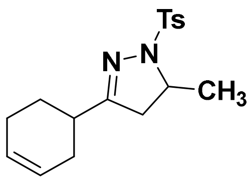

3-(cyclohex-3-en-1-yl)-5-methyl-1-tosyl-4,5-dihydro-1*H*-pyrazole (2r). Yield of **2r**: 17.5 mg in 55% isolated yield,% as a colorless oil. ^{1}H NMR (600 MHz, $CDCl_3$) δ (ppm) = 7.74 (dd, *J* = 8.4, 3.0 Hz, 2H), 7.29 (dd, *J* = 8.4, 3.0 Hz, 2H), 5.68-5.63 (m, 2H), 3.72-3.64 (m, 1H), 2.75-2.66 (m, 1H), 2.55-2.47 (m, 1H), 2.42 (s, 3H), 2.41-2.32 (m, 1H), 2.12-1.97 (m, 4H), 1.80 (dd, *J* = 13.2, 3.0 Hz, 1H), 1.62-1.61 (m, 1H), 1.52 (dd, *J* = 6.0, 3.6 Hz, 3H), 1.51-1.46 (m, 1H).

^{13}C NMR (150 MHz, $CDCl_3$) δ (ppm) δ = 165.79 (165.65), 143.89 (143.87), 131.97 (131.91), 129.19, 128.80 (128.78), 126.87 (126.76), 125.19 (125.15), 57.40 (57.37), 42.18 (42.05), 35.33 (35.24), 28.49 (28.33), 26.25, 25.85, 24.66 (24.44), 21.74 (21.57).

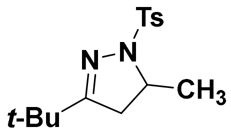

3-(tert-butyl)-5-methyl-1-tosyl-4,5-dihydro-1*H*-pyrazole (2s). Yield of **2s**: 22.0 mg in 75% isolated yield, as a colorless oil. ^{1}H NMR (600 MHz, $CDCl_3$) δ (ppm) = 7.74 (d, *J* = 8.4 Hz, 2H), 7.29 (d, *J* = 8.4 Hz, 2H), 3.69-3.62 (m, 1H), 2.70 (dd, *J* = 17.4, 10.2 Hz, 1H), 2.42 (s, 3H), 2.33 (dd, *J* = 17.4, 9.6 Hz, 1H), 1.51 (d, *J* = 6.0 Hz, 3H), 1.06 (s, 9H).

^{13}C NMR (150 MHz, $CDCl_3$) δ (ppm) δ = 169.71, 143.83, 131.80, 129.02, 128.81, 57.94, 40.56, 34.10, 27.79, 21.68, 21.50.

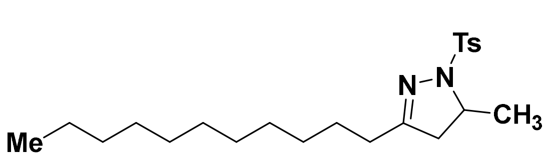

5-methyl-1-tosyl-3-undecyl-4,5-dihydro-1*H*-pyrazole (2t). Yield of **2t**: 32.1 mg in 82% isolated yield, as a yellow oil. ^{1}H NMR (600 MHz, $CDCl_3$) δ (ppm) = 7.75 (d, *J* = 8.4 Hz, 2H), 7.29 (d, *J* = 8.4 Hz, 2H), 3.73-3.66 (m, 1H), 2.68 (dd, *J* = 17.4, 10.2 Hz, 1H),

2.42 (s, 3H), 2.33 (dd, J = 17.4, 9.6 Hz, 1H), 2.29-2.17 (m, 2H), 1.52 (d, J = 6.0 Hz, 3H), 1.46 -1.38 (m, 2H), 1.31-1.21 (m, 12H), 1.17-1.06 (m, 2H), 0.89 (t, J = 7.2 Hz, 3H).

^{13}C NMR (150 MHz, $CDCl_3$) δ (ppm) = 162.88, 143.82, 132.04, 129.25, 128.73, 57.29, 43.72, 31.85, 30.07, 29.57, 29.43, 29.28, 29.24, 28.99, 26.11, 22.62, 21.78, 21.53, 14.05.

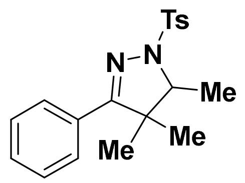

4,4,5-trimethyl-3-phenyl-1-tosyl-4,5-dihydro-1*H*-pyrazole (2u). Yield of **2u**: 19.1 mg in 56% isolated yield, as a white solid. ^{1}H NMR (600 MHz, $CDCl_3$) δ (ppm) = 7.81 (d, J = 8.4 Hz, 2H), 7.63-7.61 (m, 2H), 7.38-7.34 (m, 3H), 7.32 (d, J = 7.8 Hz, 2H), 3.05 (q, J = 6.6 Hz, 1H), 2.41 (s, 3H), 1.51 (d, J = 6.6 Hz, 3H), 1.23 (s, 3H), 1.10 (s, 3H).

^{13}C NMR (150 MHz, $CDCl_3$) δ (ppm) δ = 164.33, 144.18, 131.32, 130.84, 129.83, 129.38, 129.34, 128.87, 128.39, 127.43, 68.50, 50.79, 24.26, 21.59, 19.19, 13.22.

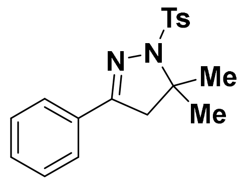

5,5-dimethyl-3-phenyl-1-tosyl-4,5-dihydro-1*H*-pyrazole (2v). Yield of **2v**: 22.0 mg in 67% isolated yield, as a white solid. ^{1}H NMR (600 MHz, $CDCl_3$) δ (ppm) = 7.93 (d, J = 8.4 Hz, 2H), 7.66-7.64 (m, 2H), 7.38-7.37 (m, 3H), 7.28 (d, J = 8.4 Hz, 2H), 3.04 (s, 2H), 2.40 (s, 3H), 1.56 (s, 6H).

^{13}C NMR (150 MHz, $CDCl_3$) δ (ppm) = 153.40, 143.40, 137.31, 131.45, 130.01, 129.22, 128.52, 128.07, 126.44, 69.10, 49.24, 27.09, 21.52.

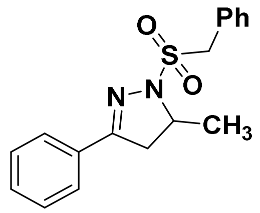

1-(benzylsulfonyl)-5-methyl-3-phenyl-4,5-dihydro-1*H*-pyrazole (2w). Yield of **2w**: 21.0 mg in 67% isolated yield, as a white solid. ^{1}H NMR (600 MHz, $CDCl_3$) δ (ppm) = 7.80-7.81 (m, 2H), 7.44-7.43 (m, 3H), 7.41-7.39 (m, 2H), 7.33-7.32 (m, 3H), 4.67 (d, *J* = 14.4 Hz, 1H), 4.54 (d, *J* = 14.4 Hz, 1H), 3.65-3.60 (m, 1H), 2.94 (dd, *J* = 14.4, 9.0 Hz, 1H), 2.84 (s, 3H), 2.82 (dd, *J* = 9.6, 2.4 Hz, 1H), 1.17 (d, *J* = 6.6 Hz, 3H).
^{13}C NMR (150 MHz, $CDCl_3$) δ (ppm) = 155.35, 136.93, 130.94, 129.78, 129.09, 128.69, 128.58, 128.50, 126.32, 55.86, 55.69, 35.83, 18.71.

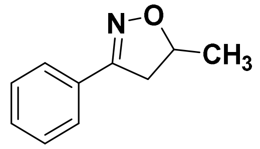

5-methyl-3-phenyl-4,5-dihydroisoxazole (2aa). Yield of **2aa**: 12.0 mg (75% isolated yield) in 365 nm as a white solid. ^{1}H NMR (600 MHz, $CDCl_3$) δ (ppm) = 7.68-7.66 (m, 2H), 7.41-7.39 (m, 3H), 4.92-4.86 (m, 1H), 3.44 (dd, *J* = 16.2, 10.2 Hz, 1H), 2.94 (dd, *J* = 16.2, 7.8 Hz, 1H), 1.44 (d, *J* = 6.0 Hz, 3H).
^{13}C NMR (150 MHz, $CDCl_3$) δ (ppm) δ = 156.45, 129.91, 129.87, 128.65, 126.58, 77.49, 41.55, 20.99.

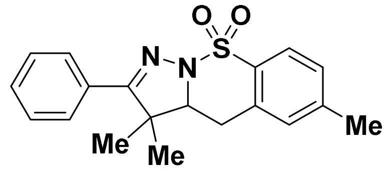

3,3,6-trimethyl-2-phenyl-3a,4-dihydro-3*H*-benzo[e]pyrazolo[1,5-*b*][1,2]thiazine 9,9-dioxide (3a). Yield of **3a**: 24.5 mg in 72% isolated yield, as a faint yellow solid. ^{1}H NMR (600 MHz, $CDCl_3$) δ (ppm) = 7.83 (d, *J* = 7.8 Hz, 1H), 7.41-7.40 (m, 2H), 7.37-7.36 (m, 1H), 7.31-7.28 (m, 2H), 7.26-7.25 (m, 1H), 7.08 (s, 1H), 4.38-4.36 (m, 1H),

3.21 (dd, J = 15.6, 7.2 Hz, 1H), 3.03 (dd, J = 15.6, 4.8 Hz, 1H), 2.37 (s, 3H), 1.55 (s, 3H), 1.36 (s, 3H).

^{13}C NMR (150 MHz, $CDCl_3$) δ (ppm) = 166.12, 143.63, 135.06, 133.08, 130.22, 130.00, 129.35, 128.40, 128.33, 127.73, 125.76, 72.01, 51.28, 27.66, 26.70, 21.55, 20.40.

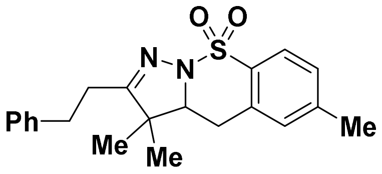

3,3,6-trimethyl-2-phenethyl-3a,4-dihydro-3*H*-benzo[e]pyrazolo[1,5-*b*][1,2]thiazine 9,9-dioxide (3b). Yield of **3b**: 25.6 mg in 70% isolated yield, as a white solid. ^{1}H NMR (600 MHz, $CDCl_3$) δ (ppm) = 7.81 (d, J = 7.8 Hz, 1H), 7.24-7.21 (m, 3H), 7.18-7.15 (m, 1H), 7.09-7.06 (m, 3H), 4.20 (dd, J =7.2, 5.4 Hz, 1H), 3.16 (dd, J = 16.2, 7.2 Hz, 1H), 2.92 (dd, J = 15.6, 7.2 Hz, 1H), 2.84-2.79 (m, 1H), 2.77-2.72 (m, 1H), 2.49-2.44 (m, 1H), 2.39 (s, 3H), 2.36-2.30 (m, 1H).

^{13}C NMR (150 MHz, $CDCl_3$) δ (ppm) δ = 169.07, 143.44, 141.08, 135.04, 133.11, 129.25, 128.39, 128.35, 128.21, 126.08, 125.72, 77.21, 51.61, 32.19, 28.11, 27.88, 25.56, 21.57, 19.50.

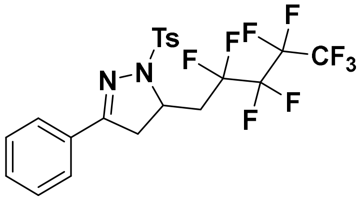

5-(2,2,3,3,4,4,5,5,5-nonafluoropentyl)-3-phenyl-1-tosyl-4,5-dihydro-1H-pyrazole (5a). Yield of **5a**: 27.6 mg in 52% isolated yield, as a brown oil. ^{1}H NMR (600 MHz, $CDCl_3$) δ (ppm) = 7.80 (d, J = 8.4 Hz, 2H), 7.67-7.65 (m, 2H), 7.45-7.42 (m, 1H), 7.40-7.38 (m, 2H), 7.31 (d, J = 7.8 Hz, 2H), 4.01-3.95 (m, 1H), 3.54-3.45 (m, 1H), 3.37-3.33 (m, 1H), 3.00 (dd, J = 16.8, 10.2 Hz, 1H), 2.60-2.50 (m, 1H), 2.40 (s, 3H).

^{13}C NMR (150 MHz, $CDCl_3$) δ (ppm) δ = 157.68, 144.85, 130.92, 130.75, 130.20, 129.72, 128.92, 128.71, 126.91, 56.06, 40.55, 37.26 (t, J = 19.5 Hz, 1C), 21.61.

^{19}F NMR (564 MHz, $CDCl_3$) δ (ppm) δ = -80.84 (t, *J* = 11.3 Hz, 3F), -111.22 - -114.22 (m, 2F), -124.20 - -124.21 (m, 2F), -125.71 - -125.82 (m, 2F).

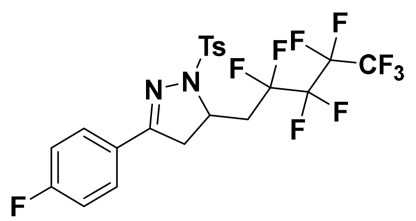

3-(4-fluorophenyl)-5-(2,2,3,3,4,4,5,5,5-nonafluoropentyl)-1-tosyl-4,5-dihydro-1*H*-pyrazole (5b). Yield of **5b**: 30.0 mg in 55% isolated yield, as a brown oil. ^{1}H NMR (600 MHz, $CDCl_3$) δ (ppm) = 7.79 (d, *J* = 7.8 Hz, 2H), 7.67-7.64 (m, 2H), 7.32 (d, *J* = 8.4 Hz, 2H), 7.09-7.06 (m, 2H), 4.02-3.96 (m, 1H), 3.54-3.44 (m, 1H), 3.36-3.31 (m, 1H), 2.98 (dd, *J* = 16.8, 10.2 Hz, 1H), 2.60-2.49 (m, 1H), 2.40 (s, 3H).

^{13}C NMR (150 MHz, $CDCl_3$) δ (ppm) = 164.29 (d, *J* = 19.5 Hz, 1C), 156.64, 144.92, 130.71, 129.74, 128.99, 128.93, 128.91, 126.48 (d, *J* = 3.0 Hz, 1C), 116.01, 115.86, 56.13, 40.58, 37.14 (t, *J* = 19.5 Hz, 1C), 21.62.

^{19}F NMR (564 MHz, $CDCl_3$) δ (ppm) = -80.84 (t, *J* = 5.6 Hz, 3F), -108.15 - -108.20 (m, 1F), -111.20 - -114.21 (m, 2F), -124.20 - -124.21 (m, 2F), -125.71 - -125.82 (m, 2F).

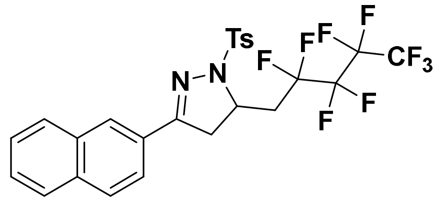

3-(naphthalen-2-yl)-5-(2,2,3,3,4,4,5,5,5-nonafluoropentyl)-1-tosyl-4,5-dihydro-1*H*-pyrazole (5c). Yield of **5c**: 23.3 mg in 40% isolated yield, as a brown oil. ^{1}H NMR (600 MHz, $CDCl_3$) δ (ppm) = 7.98 (dd, *J* = 8.4, 1.8 Hz, 2H), 7.88 (s, 1H), 7.85-7.82 (m, 5H), 7.56-7.50 (m, 2H), 7.31 (d, *J* = 7.8 Hz, 2H), 4.06-4.00 (m, 1H), 3.58-3.47 (m, 2H), 3.12 (dd, *J* = 17.4, 10.8 Hz, 1H), 2.65-2.54 (m, 1H), 2.38 (s, 3H).

^{13}C NMR (150 MHz, $CDCl_3$) δ (ppm) δ = 162.88, 157.74, 144.87, 134.37, 132.75, 130.71, 129.72, 128.94, 128.61, 128.47, 127.85, 127.77, 127.62, 127.58, 126.88, 123.37, 56.19, 40.51, 37.22 (t, *J* = 19.5 Hz, 1C), 21.60.

^{19}F NMR (564 MHz, $CDCl_3$) δ (ppm) δ = -80.81 (t, *J* = 11.3 Hz, 3F), -111.12 - -111.66 (m, 2F), -124.16 - -124.17 (m, 2F), -125.68 - -125.79 (m, 2F).

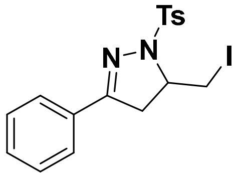

5-(iodomethyl)-3-phenyl-1-tosyl-4,5-dihydro-1*H*-pyrazole (7a). Yield of **7a**: 18.9 mg in 43% isolated yield, as a white solid. ^{1}H NMR (600 MHz, $CDCl_3$) δ (ppm) = 7.80 (d, *J* = 8.4 Hz, 2H), 7.67-7.65 (m, 2H), 7.43-7.37 (m, 3H), 7.30 (d, *J* = 8.4 Hz, 2H), 4.06-4.04 (m, 1H), 3.83 (dd, *J* = 9.6, 3.0 Hz, 1H), 3.44 (t, *J* = 9.6 Hz, 1H), 3.32 (dd, *J* = 17.4, 10.8 Hz, 1H), 3.00 (dd, *J* = 17.4, 8.4 Hz, 1H), 2.39 (s, 3H).

^{13}C NMR (150 MHz, $CDCl_3$) δ (ppm) δ = 156.63, 144.59, 131.92, 130.78, 130.41, 129.68, 128.66, 128.61, 126.93, 62.20, 41.58, 21.60, 9.71.

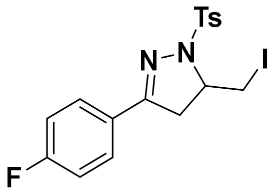

3-(4-fluorophenyl)-5-(iodomethyl)-1-tosyl-4,5-dihydro-1*H*-pyrazole (7b). Yield of **7b**: 18.3 mg in 40% isolated yield,% as a white solid. ^{1}H NMR (600 MHz, $CDCl_3$) δ (ppm) = 7.80 (d, *J* = 8.4 Hz, 2H), 7.65 (dd, *J* = 9.0, 5.4 Hz, 2H), 7.30 (d, *J* = 7.8 Hz, 2H), 7.07 (t, *J* = 9.0 Hz, 2H), 4.08-4.03 (m, 1H), 3.82 (dd, *J* = 10.2, 3.0 Hz, 1H), 3.45 (t, *J* = 9.6 Hz, 1H), 3.30 (dd, *J* = 17.4, 10.8 Hz, 1H), 2.98 (dd, *J* = 17.4, 9.0 Hz, 1H), 2.40 (s, 3H).

^{13}C NMR (150 MHz, $CDCl_3$) δ (ppm) δ = 164.23 (d, *J* = 252.0 Hz), 163.40, 155.55, 144.64, 132.07, 129.68, 129.00, 128.94, 128.62, 126.74 (d, *J* = 3.6 Hz), 115.87 (d, *J* = 21.8 Hz), 62.23, 41.65, 21.60, 9.60.

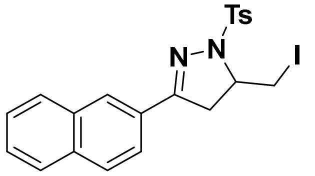

5-(iodomethyl)-3-(naphthalen-2-yl)-1-tosyl-4,5-dihydro-1*H*-pyrazole (7c). Yield of **7c**: 17.1 mg in 35% isolated yield, as a yellow solid. ^{1}H NMR (600 MHz, $CDCl_3$) δ (ppm) = 7.97 (dd, *J* = 8.4, 1.8 Hz, 1H), 7.88 (s, 1H), 7.84-7.83 (m, 5H), 7.55-7.50 (m, 2H), 7.30 (d, *J* = 8.4 Hz, 2H), 4.11-4.06 (m, 1H), 3.87 (dd, *J* = 9.6, 3.0 Hz, 1H), 3.50-3.43 (m, 2H), 3.12 (dd, *J* = 17.4, 9.0 Hz, 1H), 2.37 (s, 3H).

^{13}C NMR (150 MHz, $CDCl_3$) δ (ppm) = 156.71, 144.61, 134.29, 132.75, 131.85, 129.68, 128.62, 128.51, 128.46, 127.97, 127.83, 127.56, 127.53, 126.82, 123.41, 41.54, 21.59, 9.71.

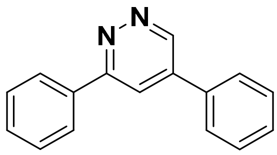

3,5-diphenylpyridazine (8). Yield of **8**: 11.8 mg in 51% isolated yield, as a white solid. ^{1}H NMR (600 MHz, $CDCl_3$) δ (ppm) = 9.42 (d, *J* = 2.4 Hz, 1H), 8.15-8.13 (m, 2H), 8.01 (d, *J* = 2.4 Hz, 1H), 7.75-7.73 (m, 2H), 7.59-7.53 (m, 6H).

^{13}C NMR (150 MHz, $CDCl_3$) δ (ppm) δ = 159.46, 148.48, 139.10, 136.49, 134.94, 130.12, 130.08, 129.55, 129.05, 127.26, 127.18, 121.05.

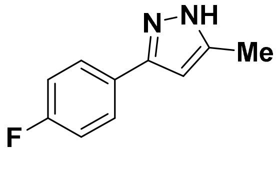

3,5-diphenylpyridazine (9). Yield of **9**: 16.7 mg in 95% isolated yield, as a white solid. ^{1}H NMR (600 MHz, $CDCl_3$) δ (ppm) = 7.68 (dd, *J* = 8.4, 5.4 Hz, 2H), 7.05 (t, *J* = 8.4 Hz, 2H), 6.29 (d, *J* = 0.6 Hz, 1H), 2.30 (d, *J* = 0.6 Hz, 2H).

^{13}C NMR (150 MHz, $CDCl_3$) δ (ppm) = 162.53 (d, *J* = 245.3 Hz, 1C), 129.01, 127.34, 127.29, 115.63, 115.48, 101.90, 11.46.

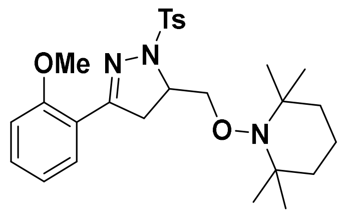

3,5-diphenylpyridazine (10). Yield of **10**: 40 mg in 80% isolated yield, as a white solid. ^{1}H NMR (600 MHz, $CDCl_3$) δ (ppm) = 7.77 (d, *J* = 7.8 Hz, 2H), 7.64 (d, *J* = 9.0 Hz, 2H), 7.26 (d, *J* = 7.8 Hz, 2H), 6.89 (d, *J* = 9.0 Hz, 2H), 4.29 (dd, *J* = 9.6, 4.2 Hz, 1H), 4.11-4.08 (m, 1H), 3.95-3.89 (m, 1H), 3.83 (s, 3H), 3.15-3.03 (m, 2H), 2.38 (s, 3H), 1.51-1.45 (m, 5H), 1.33-1.23 (m, 7H), 1.07 (d, *J* = 12.6 Hz, 6H).

^{13}C NMR (150 MHz, $CDCl_3$) δ (ppm) δ = 161.44, 158.15, 144.00, 132.09, 129.42, 128.62, 128.58, 123.55, 113.92, 78.11, 60.07, 59.87, 59.69, 55.34, 39.58, 37.49, 33.20, 32.97, 21.54, 20.07, 17.02.

Supplementary References

1. Kresse, G. & Hafner, J. Ab initio molecular-dynamics simulation of the liquid-metal-amorphous-semiconductor transition in germanium. *Phys. Rev. B* **49**, 14251-14269 (1994).
2. Blöchl, P. E. Projector Augmented-Wave Method. *Phys. Rev. B* **50**, 17953-17979 (1994).
3. Perdew, J. P., Burke, K. & Ernzerhof, M. Generalized Gradient Approximation Made Simple. *Phys. Rev. Lett.* **77**, 3865-3868 (1996).
4. Perdew, J. P., Ernzerhof, M. & Burke, K. Rationale for Mixing Exact Exchange with Density Functional Approximations. *J. Chem. Phys.* **105**, 9982-9985 (1996).
5. Grimme, S., Antony, J., Ehrlich, S. & Krieg, H. A consistent and accurate ab initio parametrization of density functional dispersion correction (DFT-D) for the 94 elements H-Pu. *J. Chem. Phys.* **132**, 154104 (2010).
6. Tang, W., Sanville, E. & Henkelman, G. A grid-based Bader analysis algorithm without lattice bias. *J. Phys.: Condens. Matter.* **21**, 084204 (2009).
7. Sanville, E., Kenny, S. D., Smith, R. & Henkelman, G. Improved grid-based algorithm for Bader charge allocation. *J. Comp. Chem.* **28**, 899-908 (2007).
8. Henkelman, G., Arnaldsson, A. & Jónsson, H. A fast and robust algorithm for Bader decomposition of charge density. *Comput. Mater. Sci.* **36**, 354-360 (2006).
9. Yu, M. & Trinkle, D. R. Accurate and efficient algorithm for Bader charge integration. *J. Chem. Phys.* **134**, 064111 (2011).
10. Hu, X. Q., Feng, G. Q., Chen, J. R., Yan, D. M., Zhao, Q. Q., Wei, Q. & Xiao, W. J. PhI(OAc)$_2$-mediated functionalisation of unactivated alkenes for the synthesis of pyrazoline and isoxazoline derivatives. *Org. Biomol. Chem.* **13**, 3457-3461 (2015).
11. Balkenhohl, M., Kölbl, S., Georgiev, T. & Carreira, E. M. Mn- and Co-Catalyzed Aminocyclizations of Unsaturated Hydrazones Providing a Broad Range of Functionalized Pyrazolines. *JACS Au* **1**, 919-924 (2021).
12. He, H., Xu, N., Zhang, H., Chen, B., Hu, Z., Guo, K., Chun, J., Cao, S. & Zhu, Y. Brønsted acid-promoted hydroamination of unsaturated hydrazones: access to biologically important 5-arylpyrazolines. *RSC Adv.* **11**, 17340-17345 (2021).
13. Yu, J. M. & Cai, C. Iodine(III)-mediated intramolecular sulfeno- and selenofunctionalization of β,γ-unsaturated tosyl hydrazones and oximes. *Org. Biomol. Chem.* **16**, 490-498 (2018).
14. Wu, X. B., Gao, Q., Fan, J. J., Zhao, Z. Y., Tu, X. Q., Cao, H. Q. & Yu J. Anionic Chiral Co(III) Complexes Mediated Asymmetric Halocyclization - Synthesis of 5-Halomethyl Pyrazolines and Isoxazolines. *Org. Lett.* **23**, 9134-9139 (2021).
15. Chen, S., Chen, W., Chen, X., Chen, G., Ackermann, L. & Tian, X. Copper(I)-Catalyzed Oxyamination of β,γ-Unsaturated Hydrazones: Synthesis of Dihydropyrazoles. *Org. Lett.* **21**, 7787-7790 (2019).
16. Mondal, H., Sk, M. R. & Maji, M. S. Cooperativity within the catalyst: alkoxyamide as a catalyst for bromocyclization and bromination of (hetero)aromatics. *Chem. Commun.* **56**, 11501-11504 (2020).
17. Meng, F., Fang, Q., Yuan, W. D., Xu, N., Cao, S. J., Chun, J. L., Li, J., Zhang, H. L. & Zhu, Y. G. Access to cyano-substituted pyrazolines through copper-catalyzed cascade cyanation/cyclization of unactivated olefins. *Org. Chem. Front.* **7**, 1358-1364 (2020).
18. Yang, M. N., Yan, D. M., Zhao, Q. Q., Chen, J. R. & Xiao, W. J. Synthesis of Dihydropyrazoles via Ligand-Free Pd-Catalyzed Alkene Aminoarylation of Unsaturated Hydrazones with Diaryliodonium Salts. *Org. Lett.* **19**, 5208-5211 (2017).
19. Xu, Z. Q., Wang, W. B., Zheng, L. C., Li, L., Duan, L. L. & Li, Y. M. Iodine-mediated aminosulfonylation

of alkenyl sulfonamides with sulfonyl hydrazides: synthesis of sulfonylmethyl piperidines, pyrrolidines and pyrazolines. *Org. Biomol. Chem.* **17**, 9026-9038 (2019).

20. Yu, W., Yang, S., Wang, P. L., Li, P. & Li, H. $BF_3 \cdot OEt_2$-mediated cyclization of β,γ-unsaturated oximes and hydrazones with N-(arylthio/arylseleno)succinimides: an efficient approach to synthesize isoxazoles or dihydropyrazoles. *Org. Biomol. Chem.* **18**, 7165-7173 (2020).
21. Meng, F., Zhang, H. L., He, H., Xu, N., Fang, Q., Guo, K., Cao, S. J., Shi, Y. & Zhu Y. G. Copper-Catalyzed Domino Cyclization/Thiocyanation of Unactivated Olefins: Access to SCN-Containing Pyrazolines. *Adv. Synth. Catal.* **362**, 248-254 (2020).
22. Chen, J., Yang, M. N., Chen, J. R. & Xiao, W. J. Palladium-Catalyzed Ring-Forming Alkene Aminoaroylation of Unsaturated Hydrazones and Sulfonamides. *Org. Lett.* **20**, 3314-3318 (2018).
23. Park, S. W., Kim, S. H., Song, J., Park, G. Y., Kim, D., Nam, T. G. & Hong, K. B. Hypervalent iodine-mediated Ritter-type amidation of terminal alkenes: The synthesis of isoxazoline and pyrazoline cores. *Beilstein. J. Org. Chem.* **14**, 1028-1033 (2018).
24. Zhao, J., Jiang, M. & Liu, J. T. Transition metal-free aminofluorination of β,γ-unsaturated hydrazones: base-controlled regioselective synthesis of fluorinated dihydropyrazole and tetrahydropyridazine derivatives. *Org. Chem. Front.* **5**, 1155-1159 (2018).
25. Chang, B. B., Su, Y. P., Huang, D. F., Wang, K. H., Zhang, W. G., Shi, Y., Zhang, X. H. & Hu, Y. L. Synthesis of Trifluoroethyl Pyrazolines via Trichloroisocyanuric Acid Promoted Cascade Cyclization/Trifluoromethylation of β,γ-Unsaturated Hydrazones. *J. Org. Chem.* **83**, 4365-4374 (2018).
26. Wei, Q., Chen, J. R., Hu, X. Q., Yang, X. C., Lu, B. & Xiao, W. J. Photocatalytic Radical Trifluoromethylation/Cyclization Cascade: Synthesis of CF_3-Containing Pyrazolines and Isoxazolines. *Org. Lett.* **17**, 4464-4467 (2015).
27. Hu, X. Q., Chen, J. R., Wei, Q., Liu, F. L., Deng, Q. H., Beauchemin, A. M. & Xiao, W. J. Photocatalytic Generation of N-Centered Hydrazonyl Radicals: A Strategy for Hydroamination of β,γ-Unsaturated Hydrazones. *Angew. Chem. Int. Ed.* ***53***, 12163-12167 (2014).
28. Hu, X. Q., Qi, X. T., Chen, J. R., Zhao, Q. Q., Wei, Q., Lan, Y. & Xiao, W. J. Catalytic N-radical cascade reaction of hydrazones by oxidative deprotonation electron transfer and TEMPO mediation. *Nat. Commun.* **7**, 1-12 (2016).
29. Hu, X. Q., Chen, J., Chen, J. R., Yan, D. M. & Xiao, W. J. Organophotocatalytic Generation of N- and O-Centred Radicals Enables Aerobic Oxyamination and Dioxygenation of Alkenes. *Chem. Eur. J.* **22**, 14141-14146 (2016).
30. Filippini, G., Longobardo, F., Forster, L., Criado, A., Di Carmine, G., Nasi, L., D'Agostino, C., Melchionna, M., Fornasiero, P. & Prato, M. Light-driven, heterogeneous organocatalysts for C-C bond formation toward valuable perfluoroalkylated intermediates, *Sci. Adv.* **6**, eabc9923 (2020).
31. Beatty, J. W., Douglas, J. J., Miller, R., McAtee, R. C., Cole, K. P. & Stephenson, C. R. J. Photochemical Perfluoroalkylation with Pyridine N-Oxides: Mechanistic Insights and Performance on a Kilogram Scale, *Chem* **1**, 456-472 (2016).
32. Sladojevich, F., McNeill, E., Bçrgel, J., Zheng, S.-L. & Ritter, T. Condensed-Phase, Halogen-Bonded CF_3I and C_2F_5I Adducts for Perfluoroalkylation Reactions, *Angew. Chem. Int. Ed.* **54**, 3712-3716 (2015).
33. Tiwari, D. P., Dabral, S., Wen, J., Wiesenthal, J., Terhorst, S. & Bolm, C. Organic Dye-Catalyzed Atom Transfer Radical Addition-Elimination (ATRE) Reaction for the Synthesis of Perfluoroalkylated Alkenes, *Org. Lett.* **19**, 4295-4298 (2017).

NMR Spectra

^{1}H NMR (600 MHz, $CDCl_3$) spectrum of product 2a

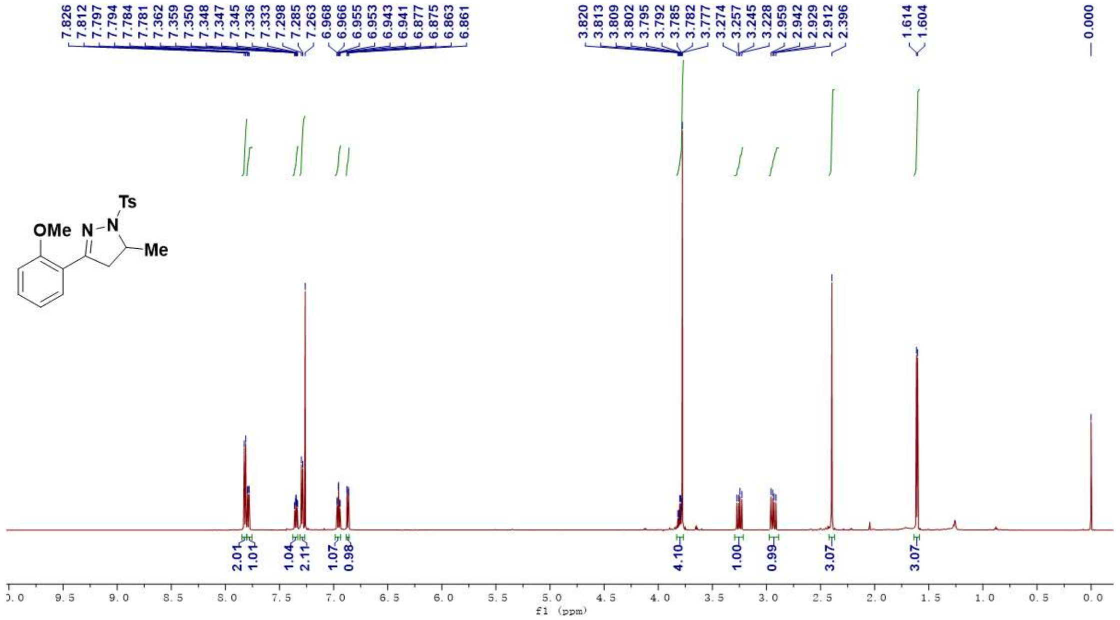

^{13}C NMR (150 MHz, $CDCl_3$) spectrum of product 2a

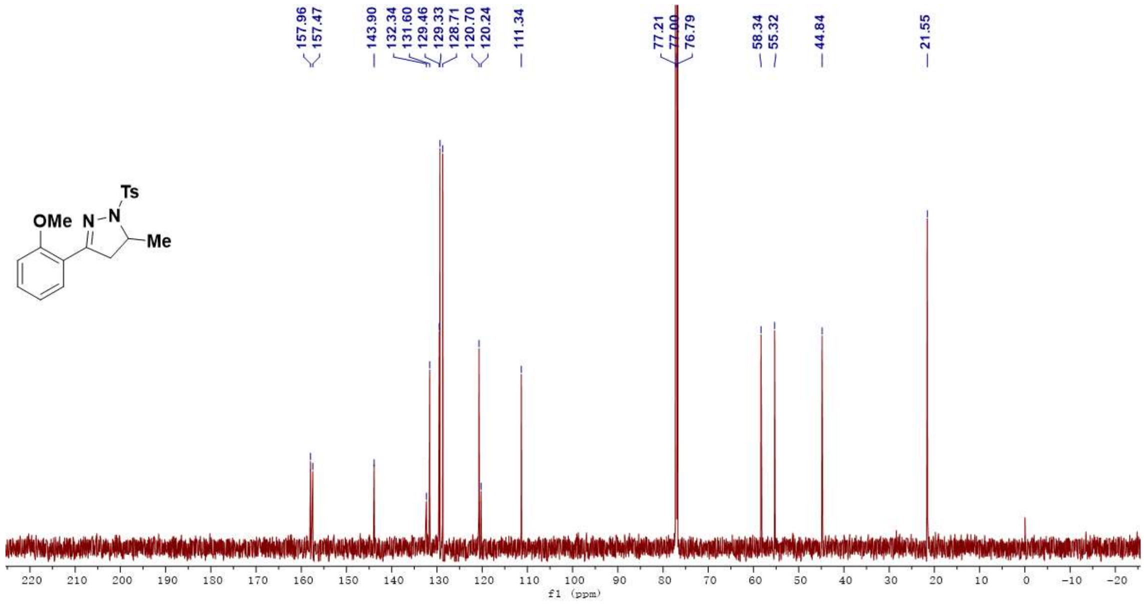

^{1}H NMR (600 MHz, CDCl3) spectrum of product 2b

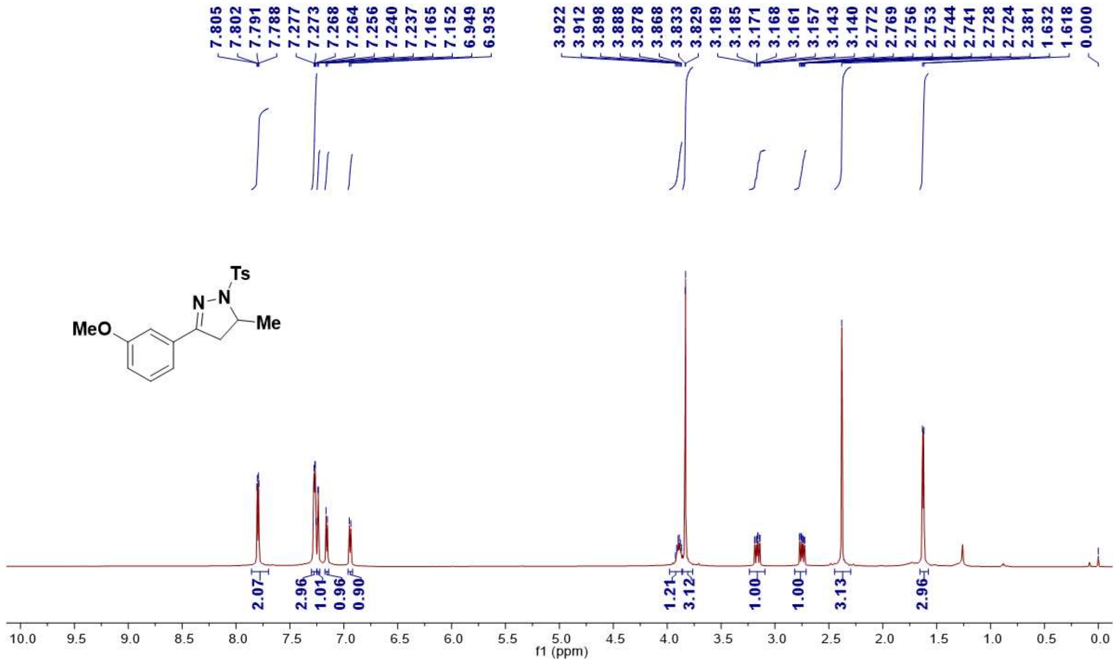

^{13}C NMR (150 MHz, CDCl3) spectrum of product 2b

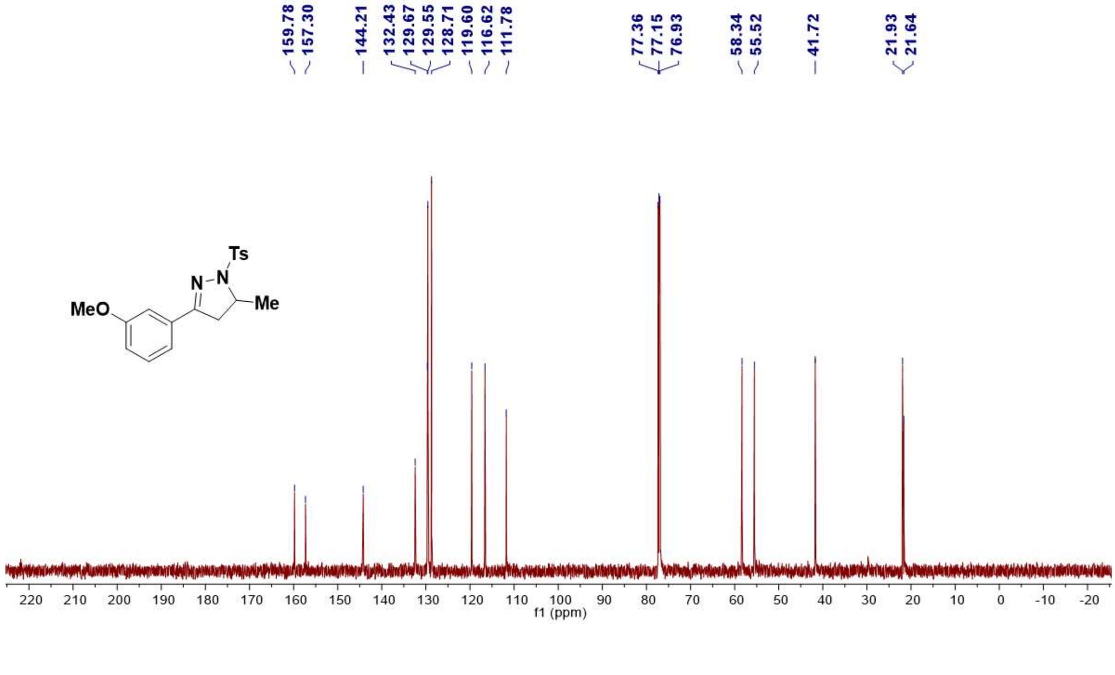

^{1}H NMR (600 MHz, CDCl₃) spectrum of product 2c

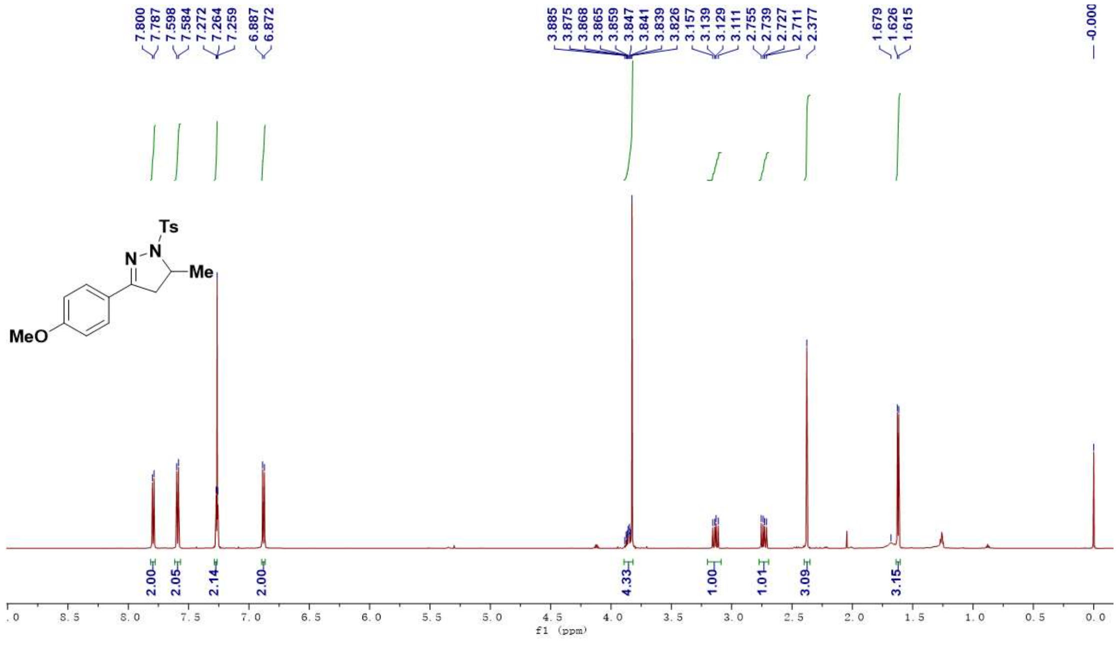

^{13}C NMR (150 MHz, CDCl₃) spectrum of product 2c

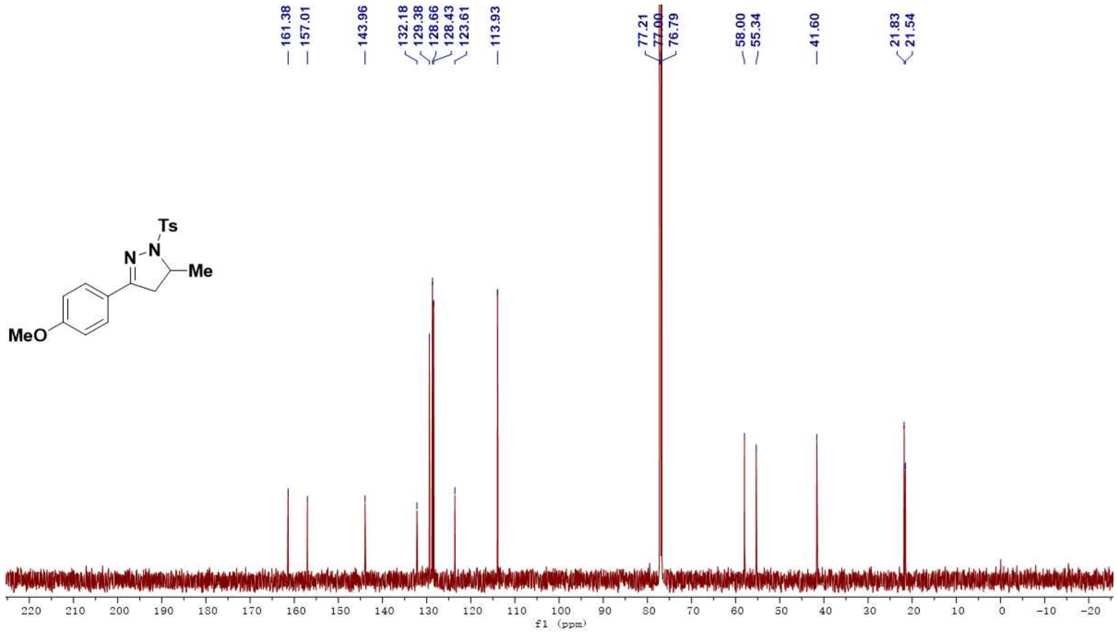

^{1}H NMR (600 MHz, CDCl3) spectrum of product 2d

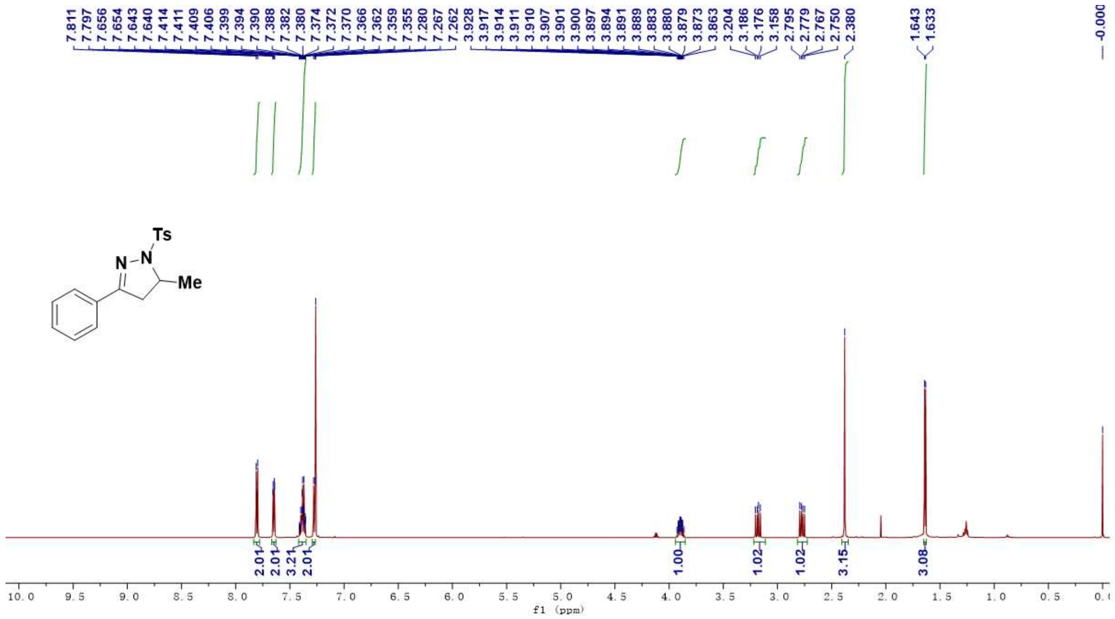

^{13}C NMR (150 MHz, CDCl3) spectrum of product 2d

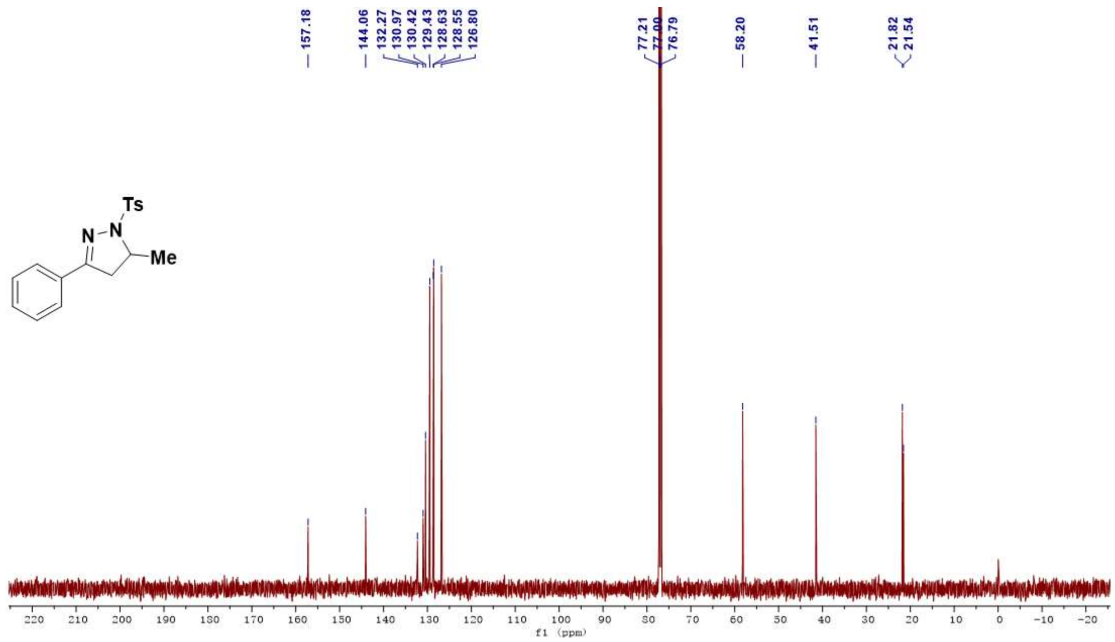

^{1}H NMR (600 MHz, $CDCl_3$) spectrum of product 2e

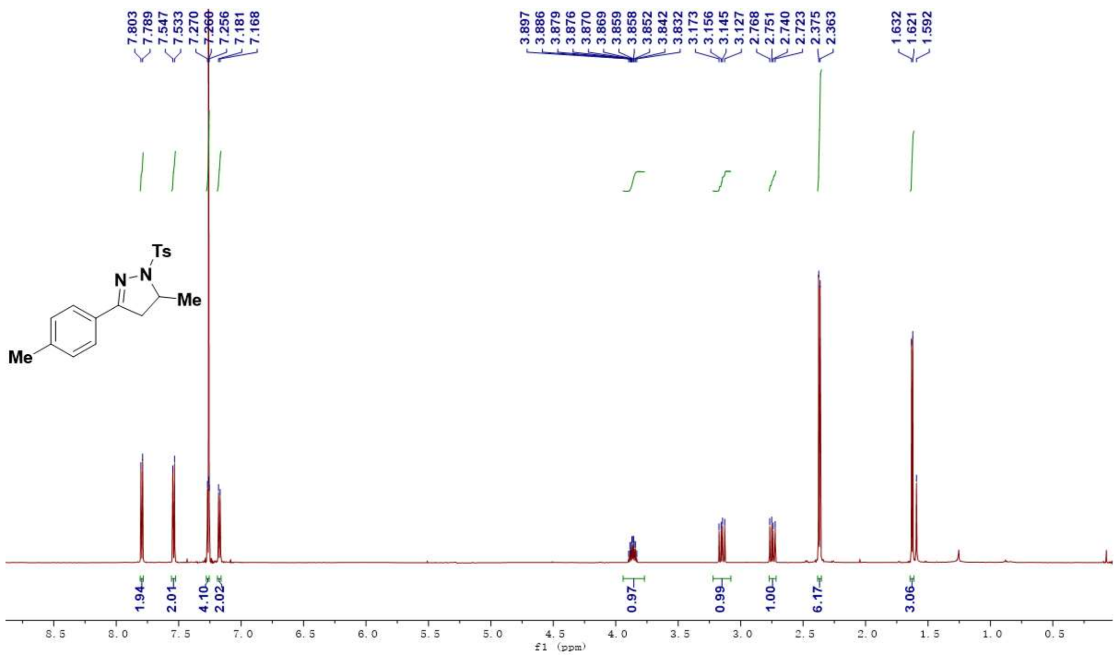

^{13}C NMR (150 MHz, $CDCl_3$) spectrum of product 2e

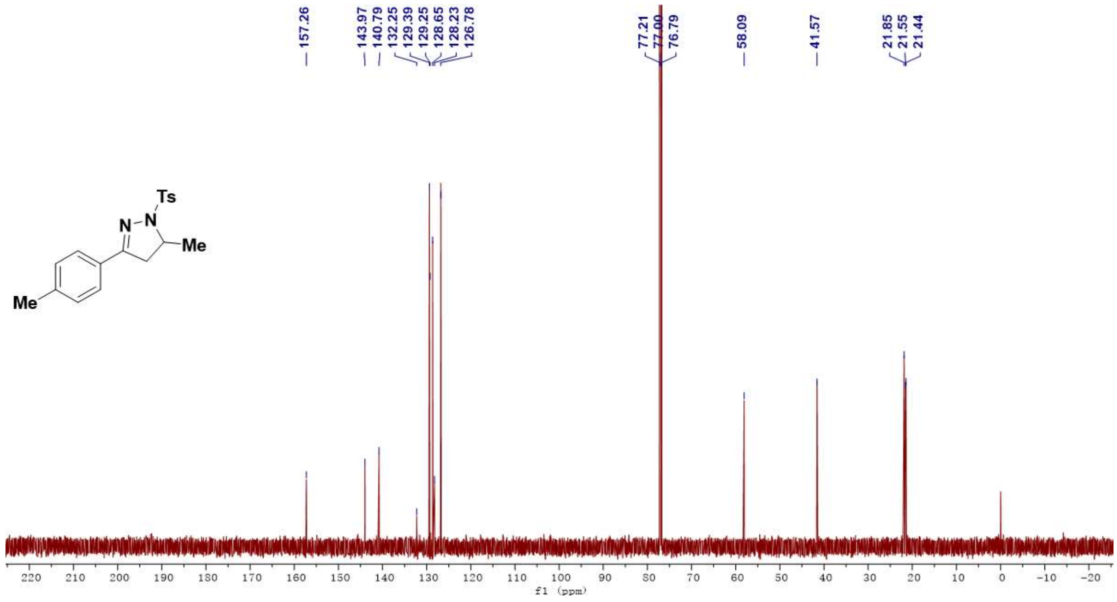

^{1}H NMR (600 MHz, $CDCl_3$) spectrum of product 2f

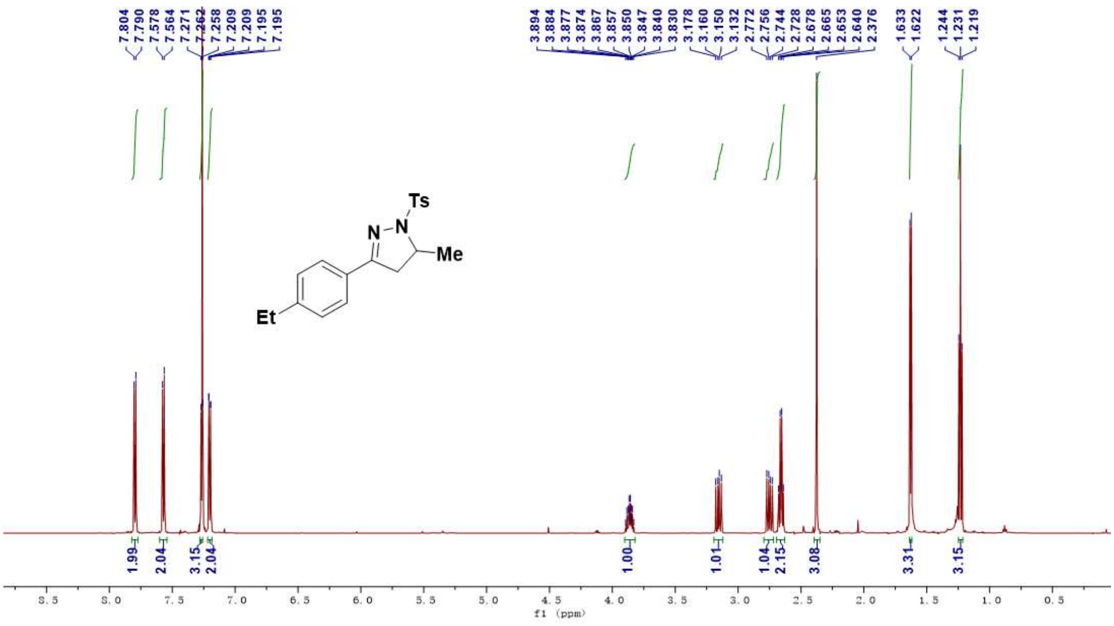

^{13}C NMR (150 MHz, $CDCl_3$) spectrum of product 2f

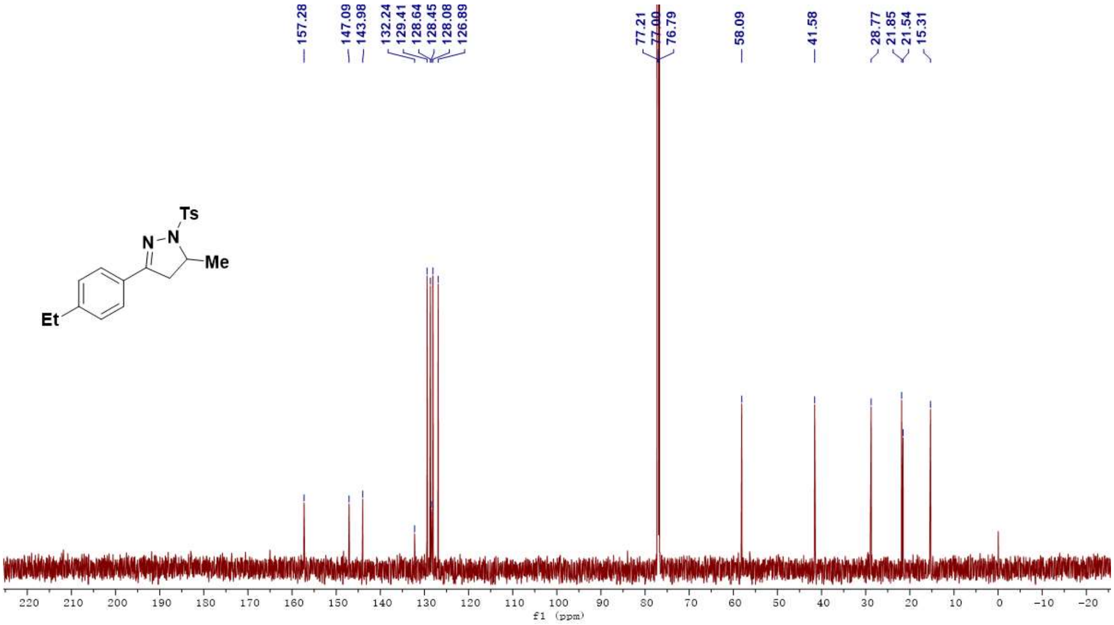

^{1}H NMR (600 MHz, CDCl3) spectrum of product 2g

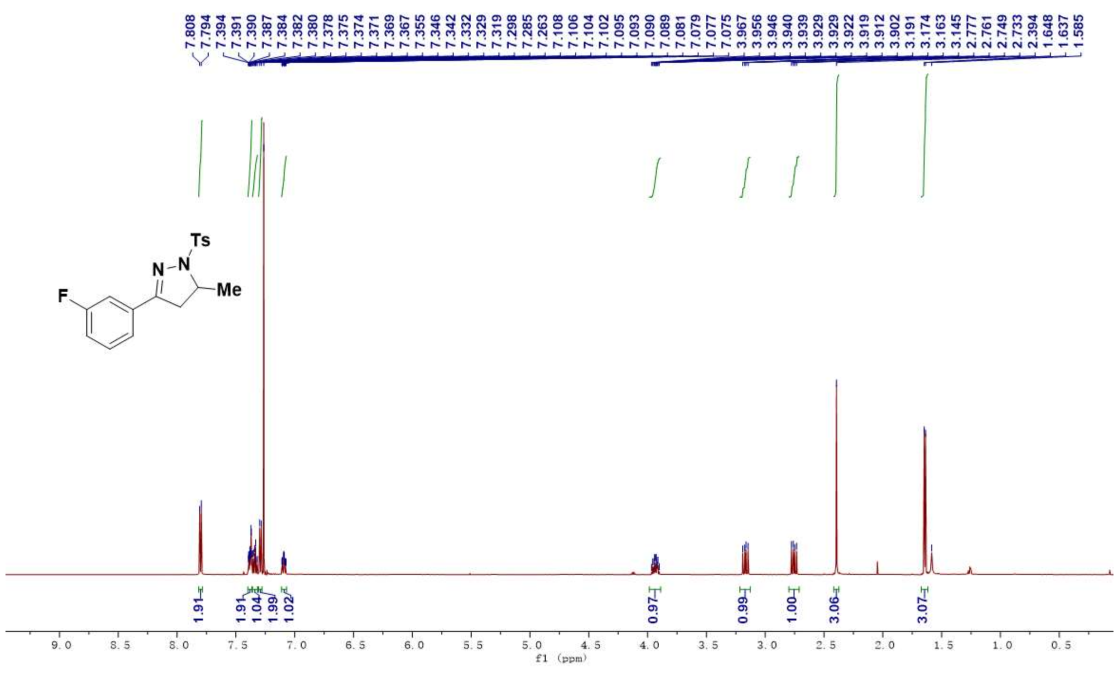

^{13}C NMR (150 MHz, CDCl3) spectrum of product 2g

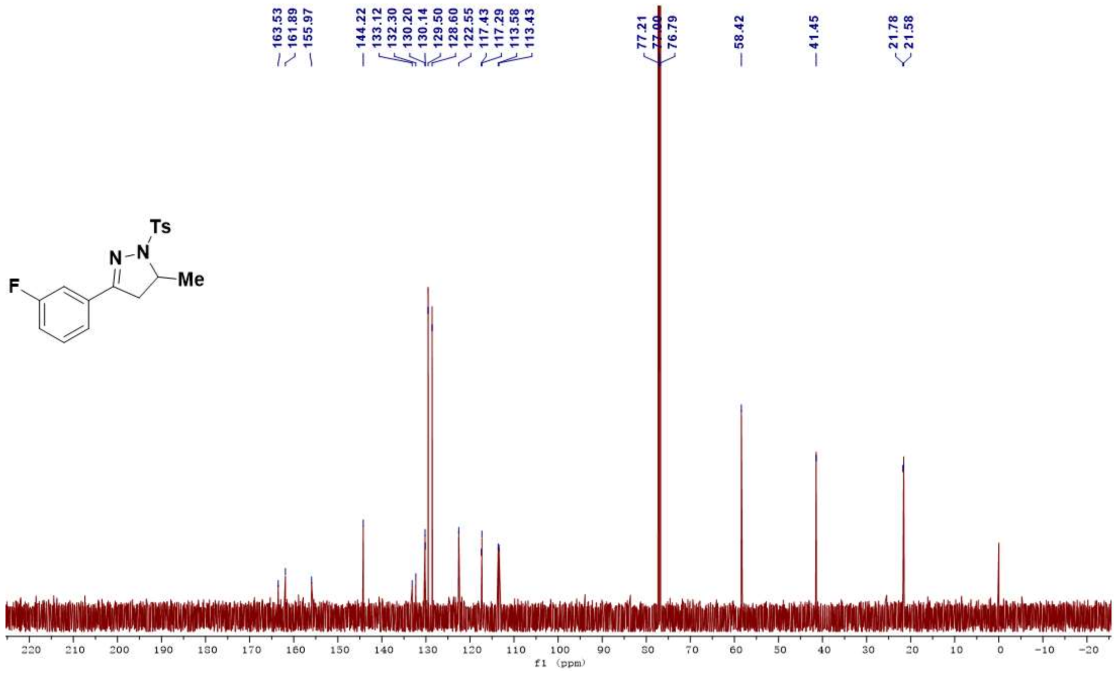

^{1}H NMR (600 MHz, $CDCl_3$) spectrum of product 2h

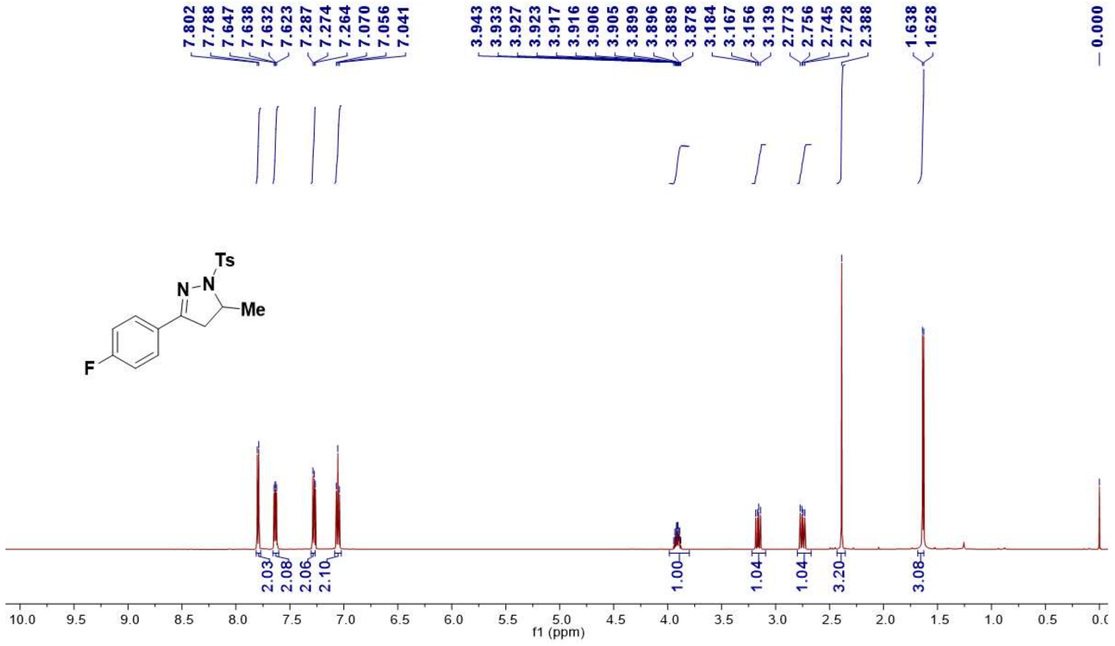

^{13}C NMR (150 MHz, $CDCl_3$) spectrum of product 2h

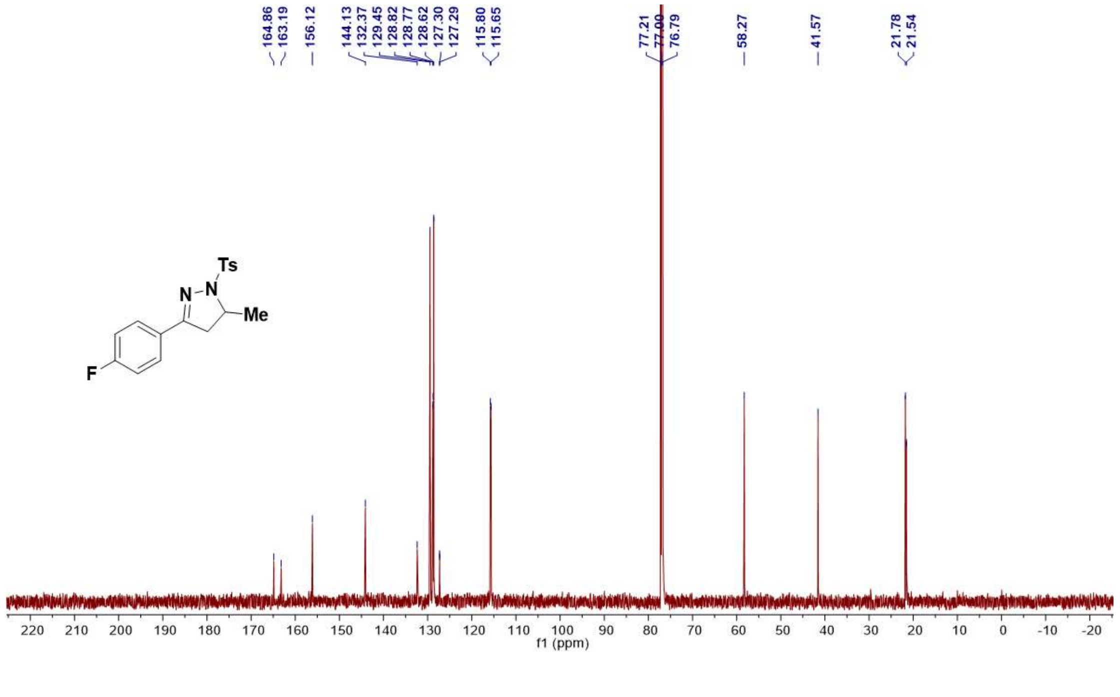

^{1}H NMR (600 MHz, CDCl$_3$) spectrum of product 2h+2h’

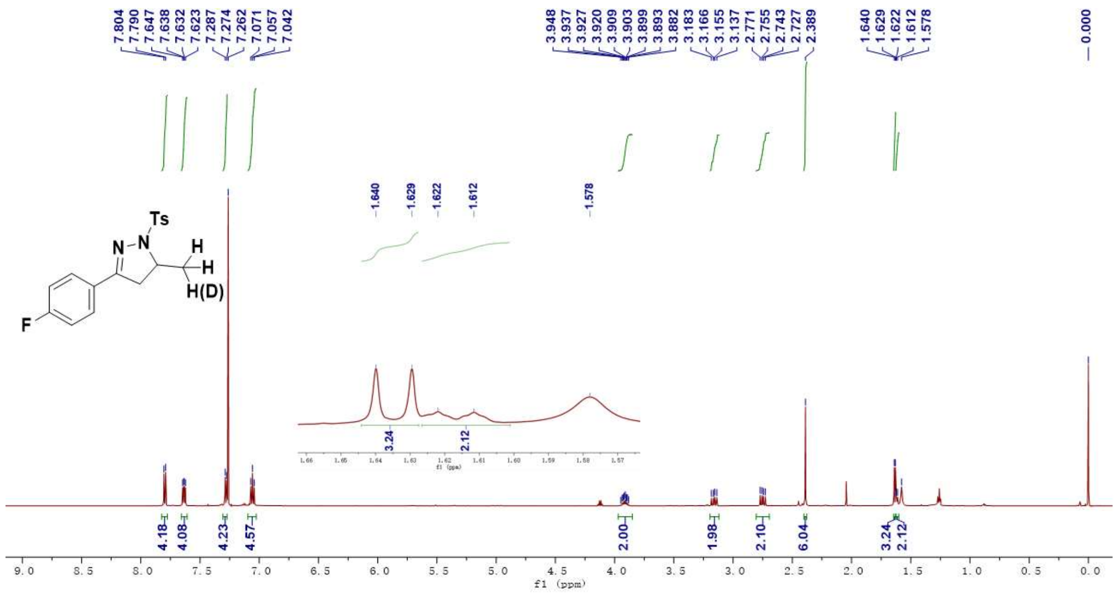

^{13}C NMR (150 MHz, CDCl$_3$) spectrum of product 2h+2h’

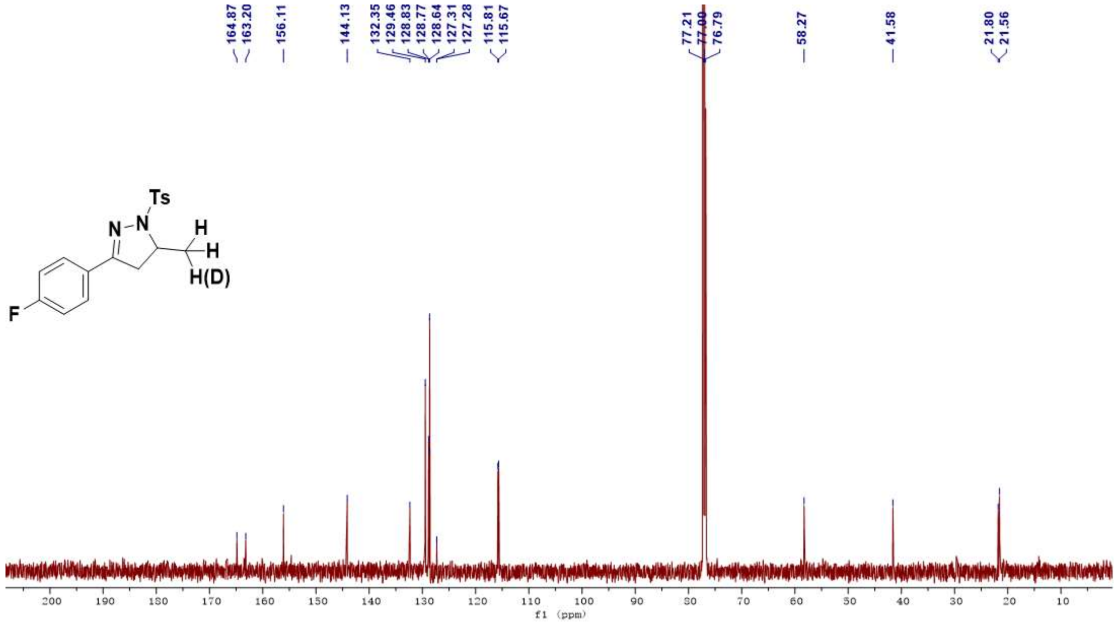

^{1}H NMR (600 MHz, CDCl3) spectrum of product 2i

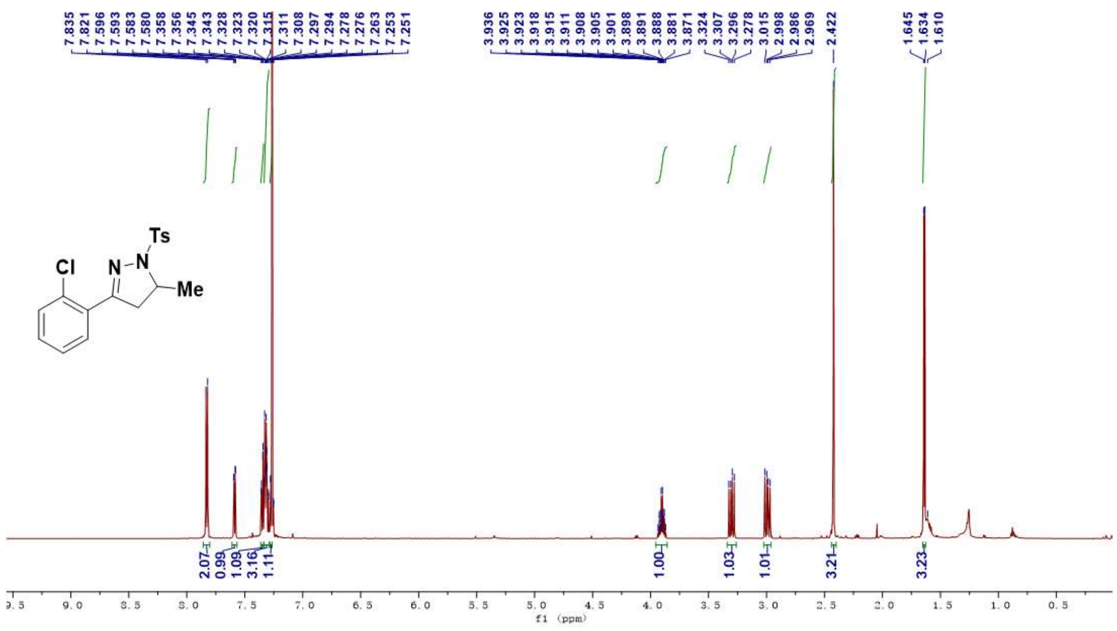

^{13}C NMR (150 MHz, CDCl3) spectrum of product 2i

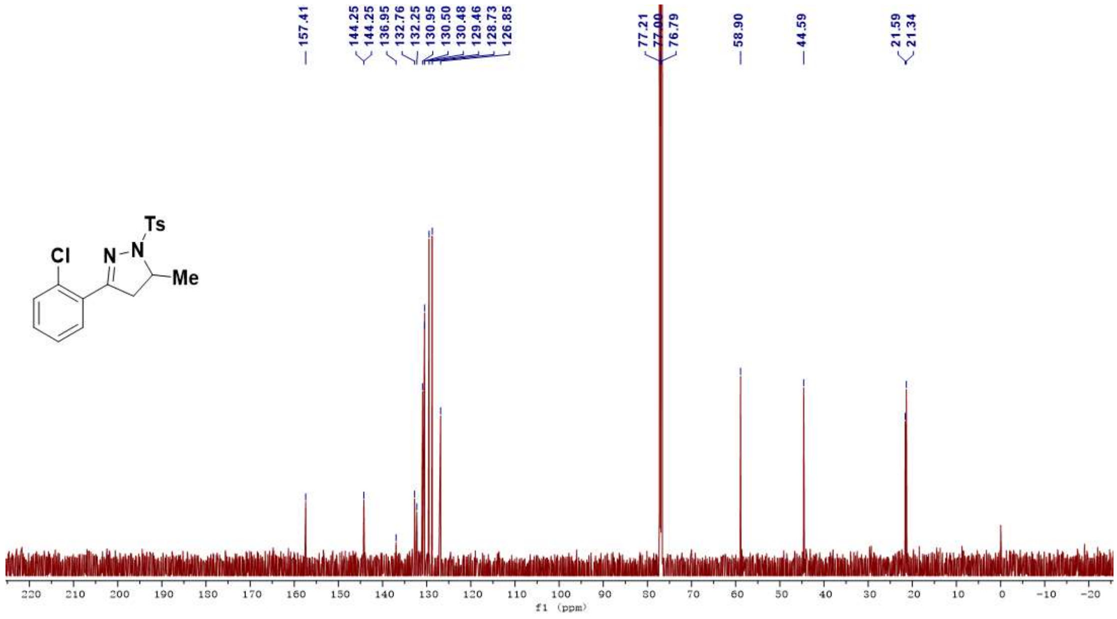

^{1}H NMR (600 MHz, CDCl3) spectrum of product 2j

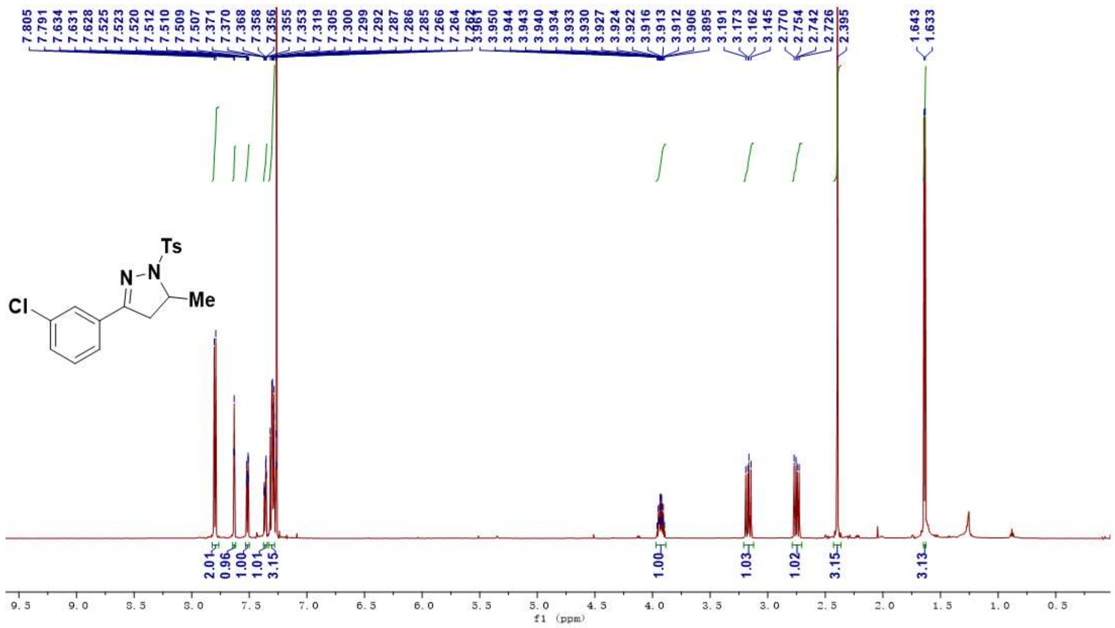

^{13}C NMR (150 MHz, CDCl3) spectrum of product 2j

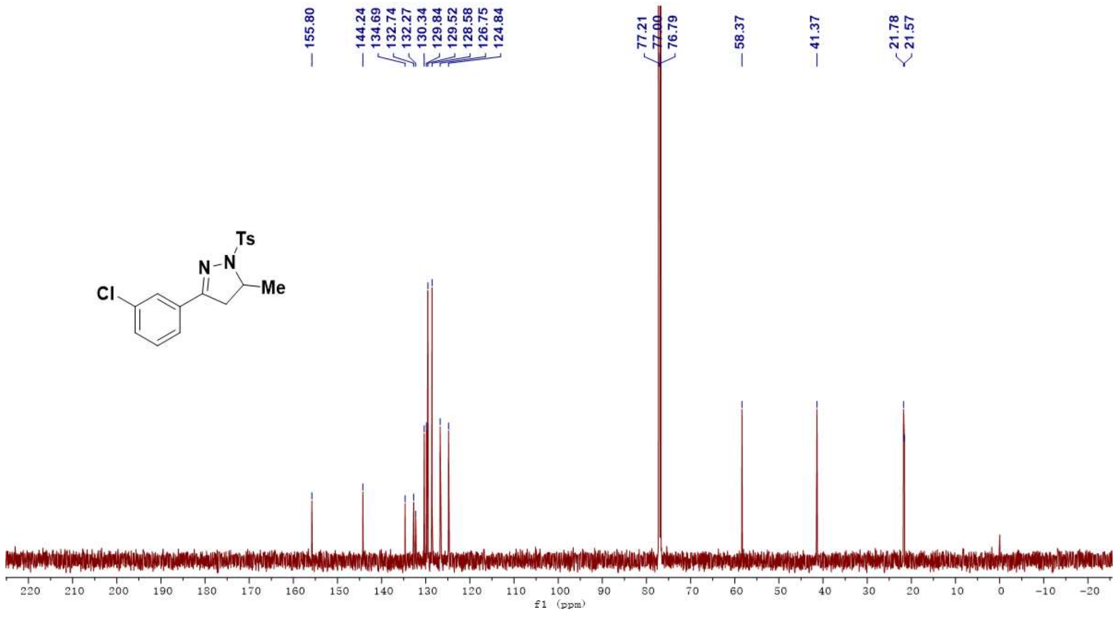

^{1}H NMR (600 MHz, $CDCl_3$) spectrum of product 2k

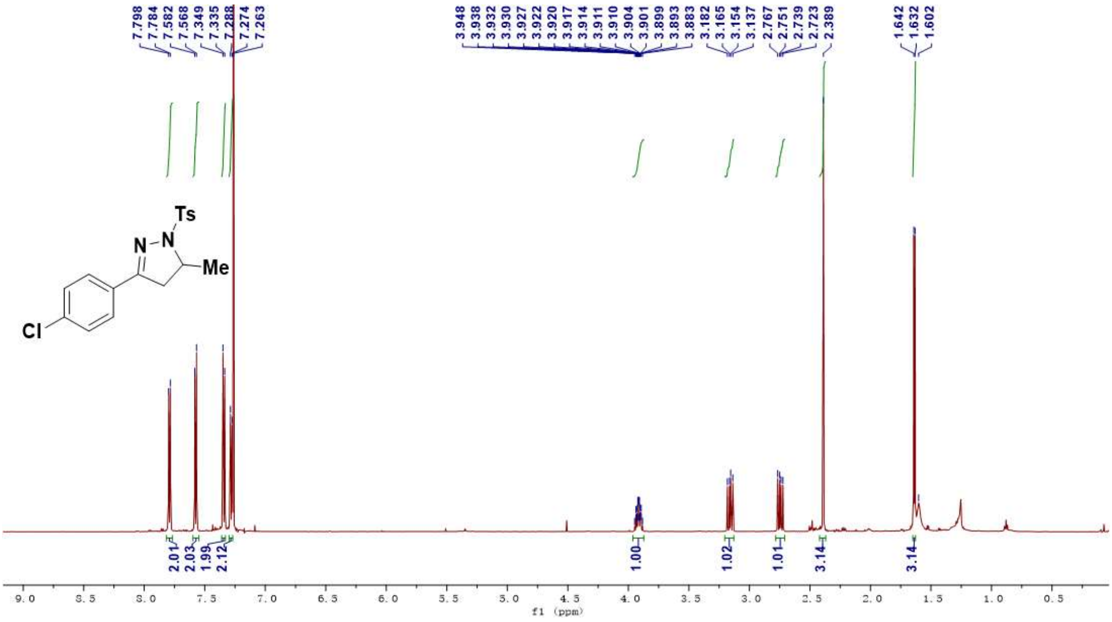

^{13}C NMR (150 MHz, $CDCl_3$) spectrum of product 2k

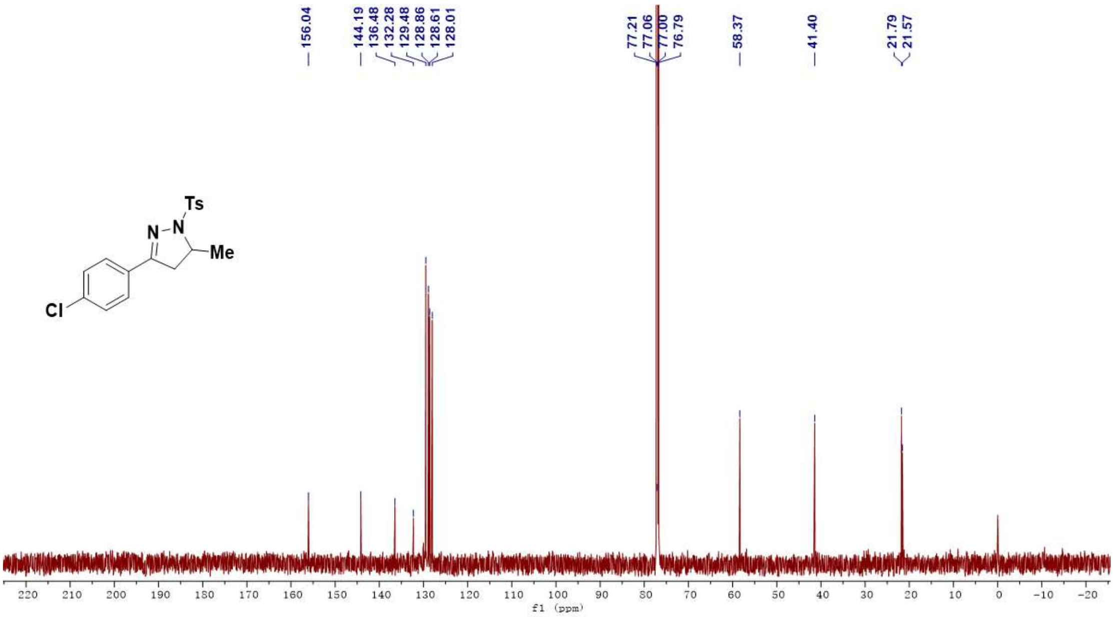

^{1}H NMR (600 MHz, $CDCl_3$) spectrum of product 2l

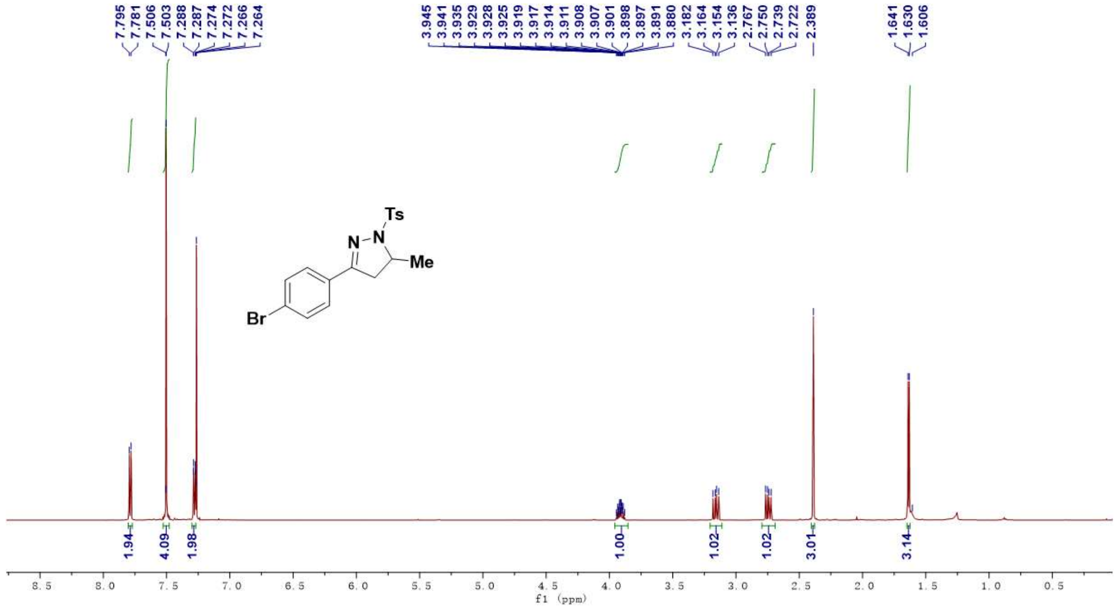

^{13}C NMR (150 MHz, $CDCl_3$) spectrum of product 2l

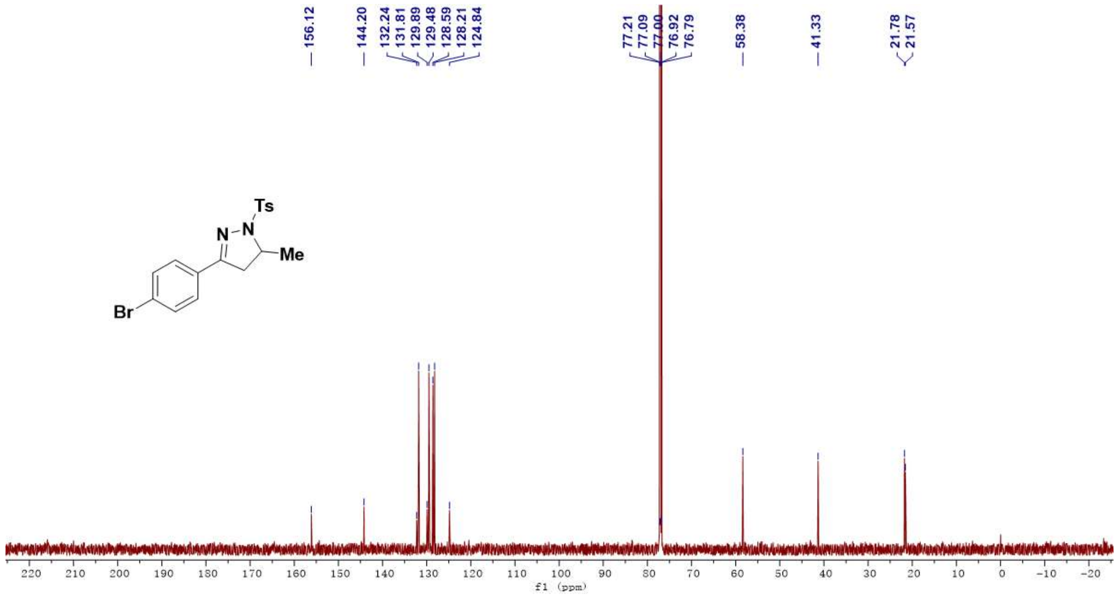

^{1}H NMR (600 MHz, $CDCl_3$) spectrum of product 2m

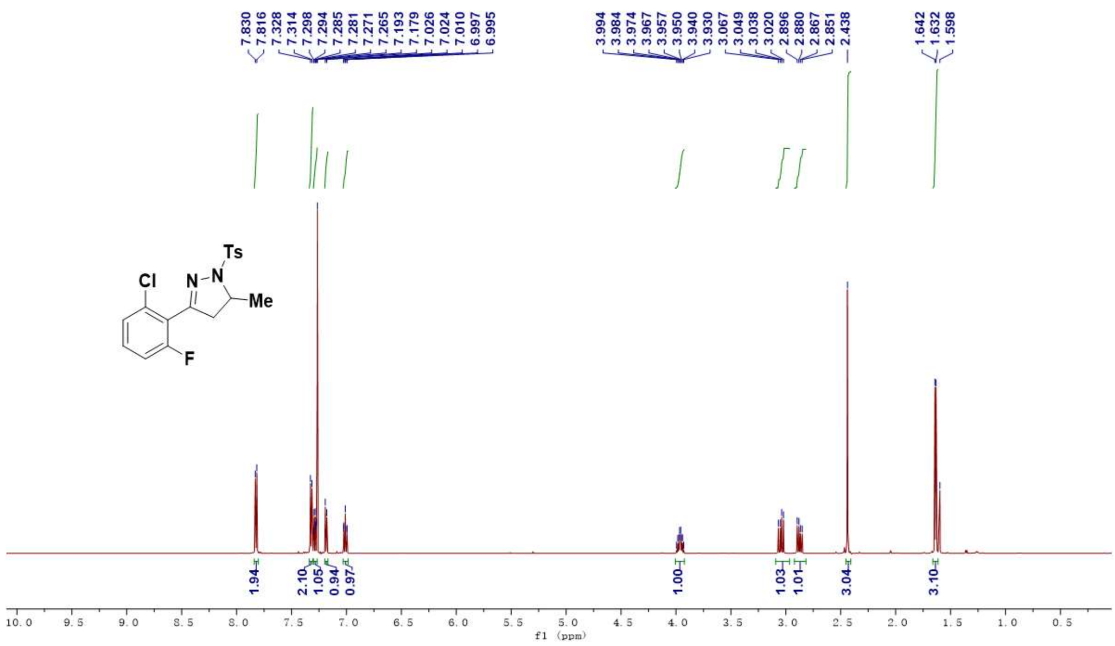

^{13}C NMR (150 MHz, $CDCl_3$) spectrum of product 2m

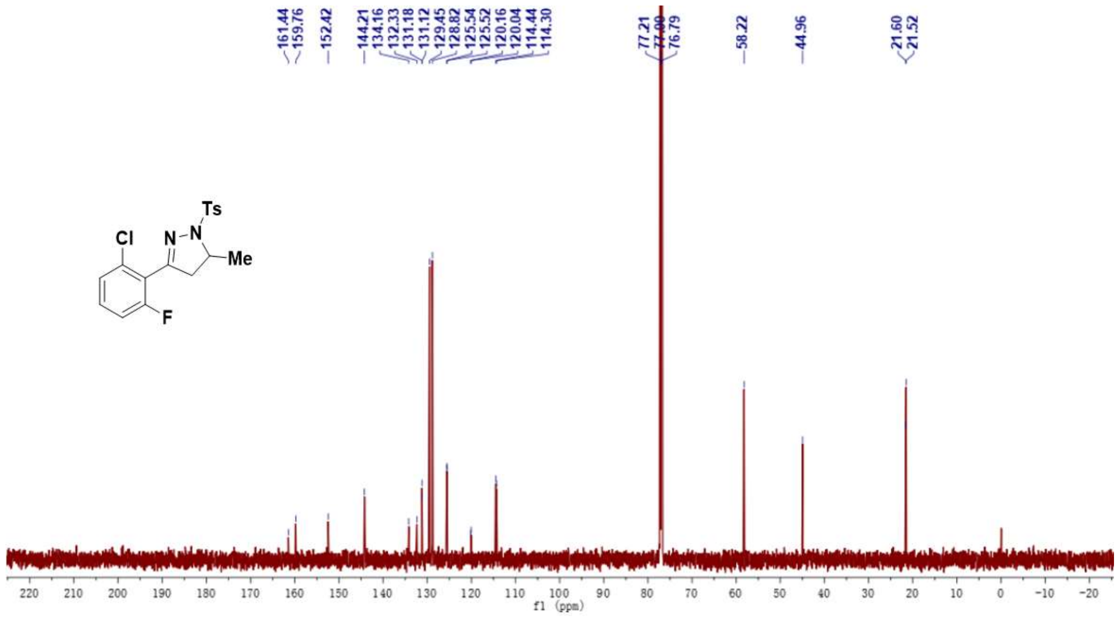

^{1}H NMR (600 MHz, $CDCl_3$) spectrum of product 2n

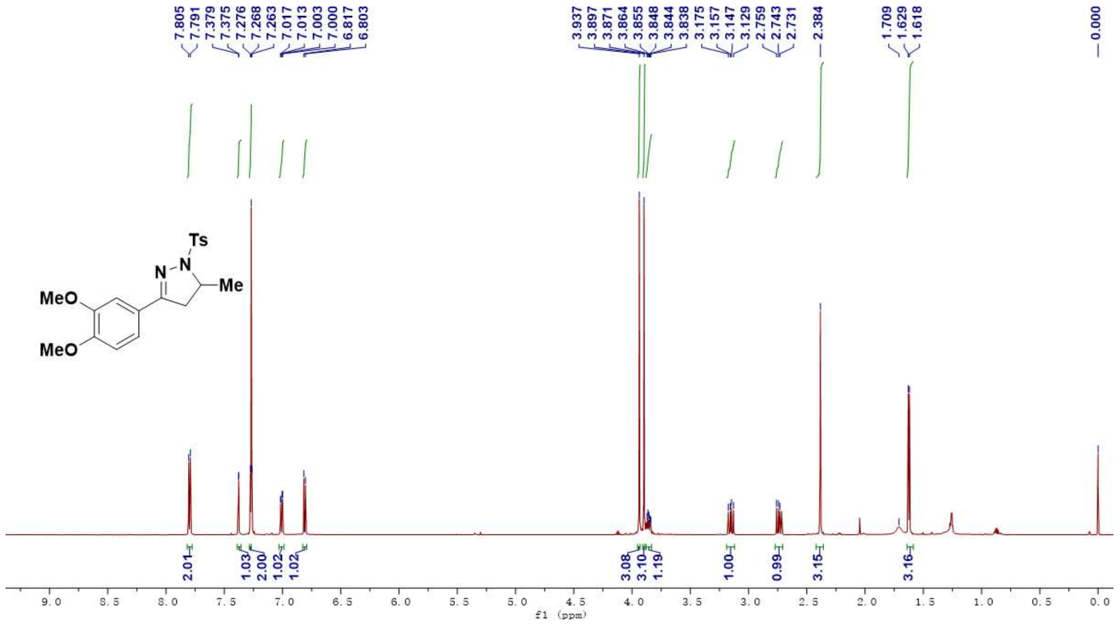

^{13}C NMR (150 MHz, $CDCl_3$) spectrum of product 2n

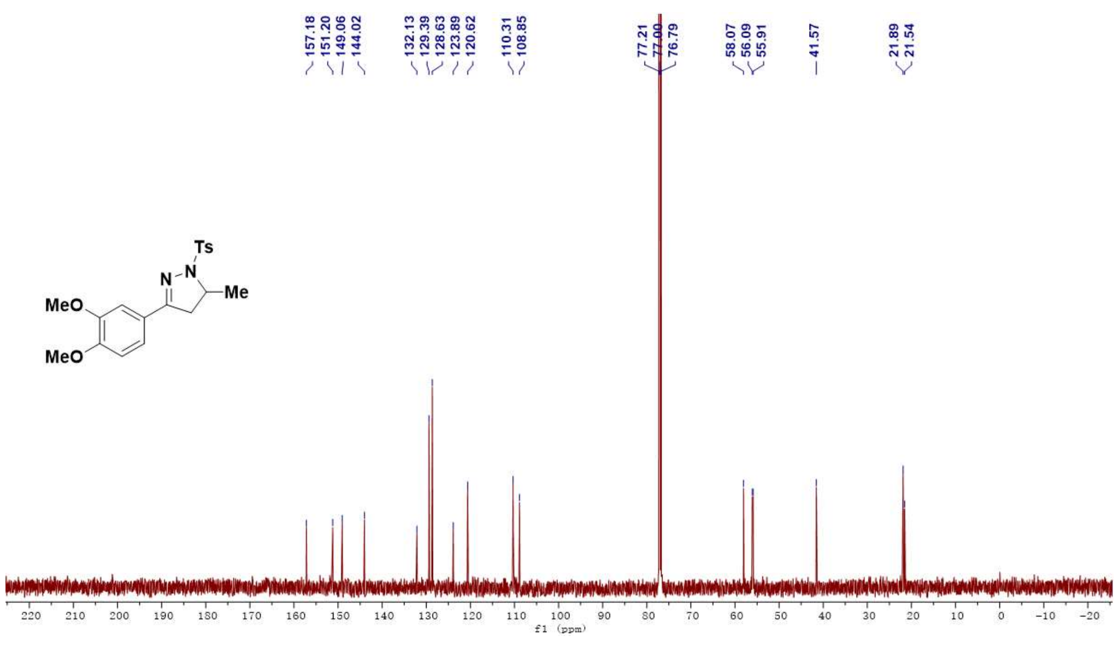

^{1}H NMR (600 MHz, CDCl3) spectrum of product 2o

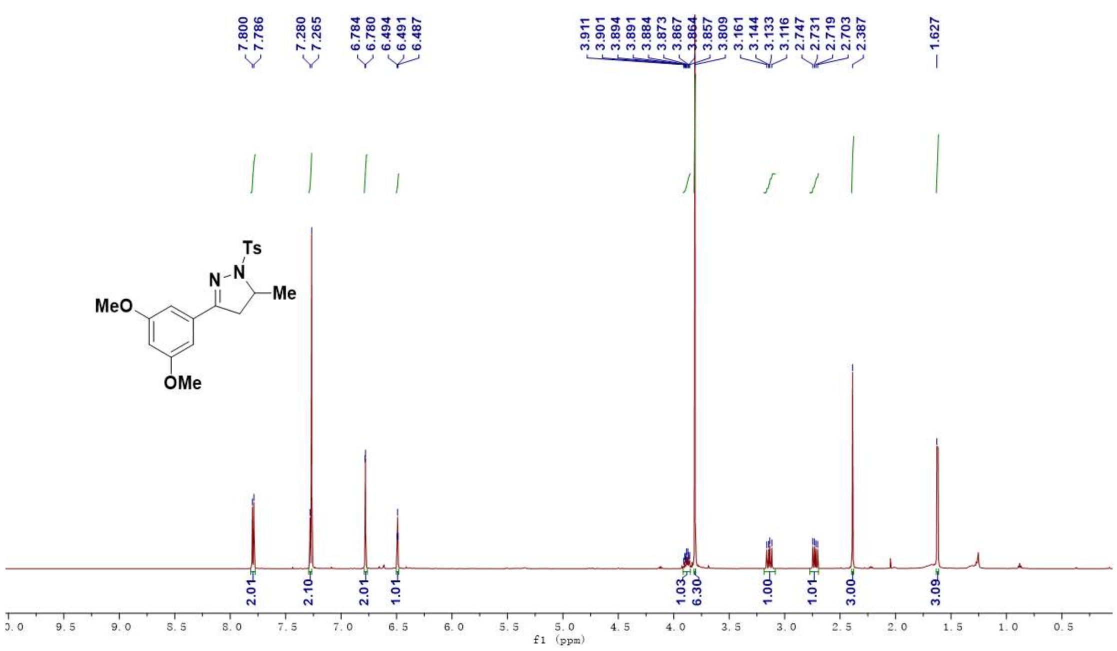

^{13}C NMR (150 MHz, CDCl3) spectrum of product 2o

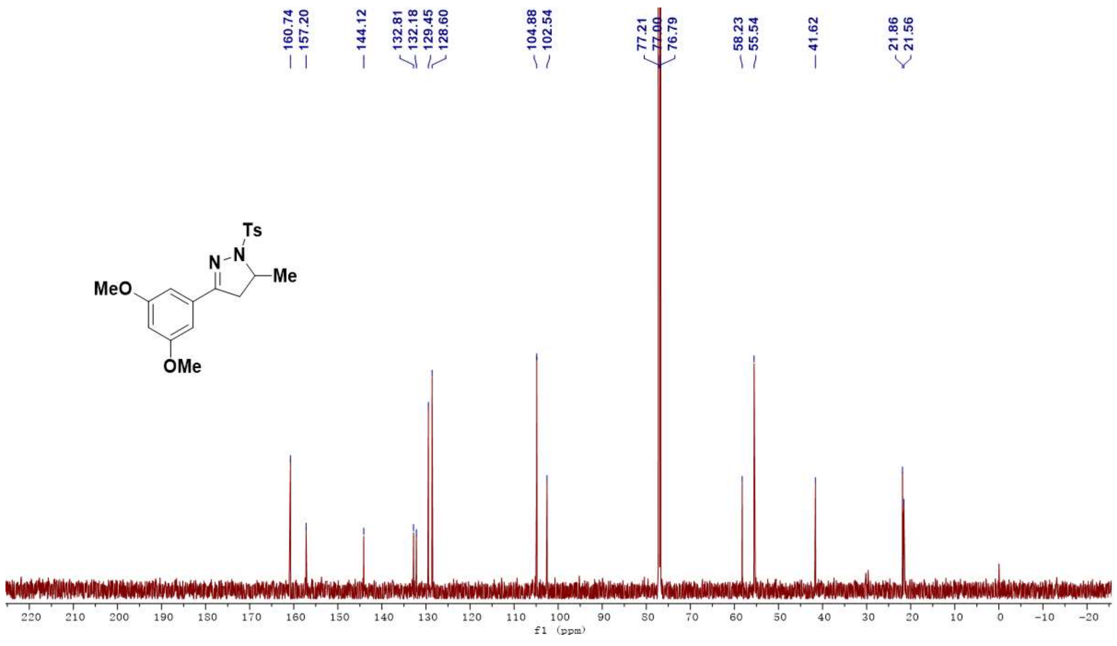

^{1}H NMR (600 MHz, $CDCl_3$) spectrum of product 2p

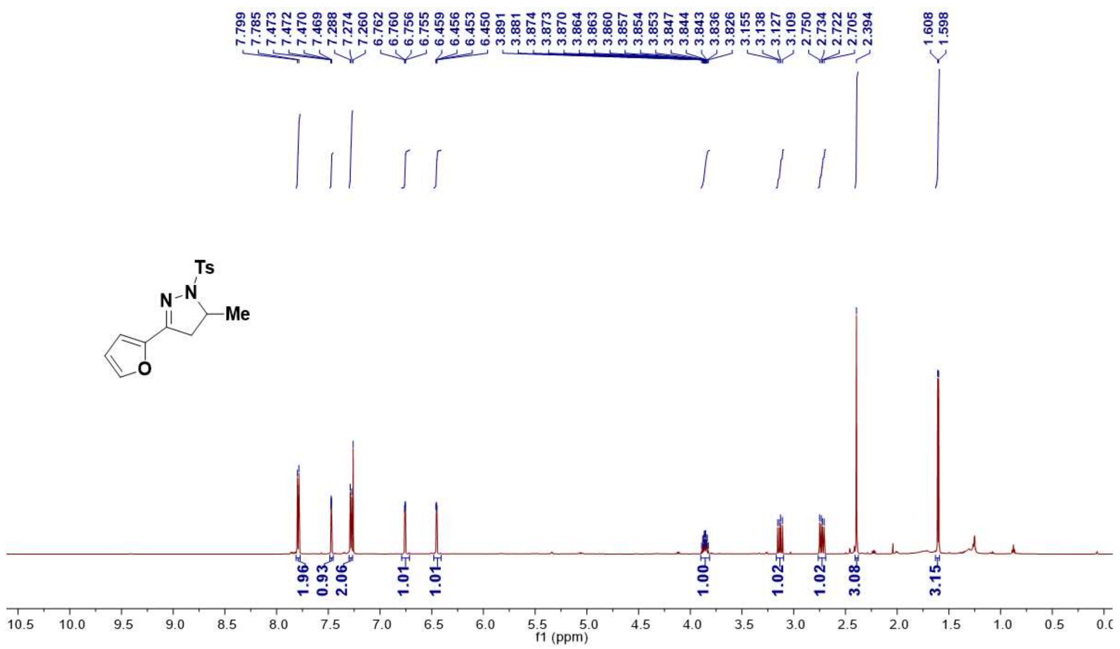

^{13}C NMR (150 MHz, $CDCl_3$) spectrum of product 2p

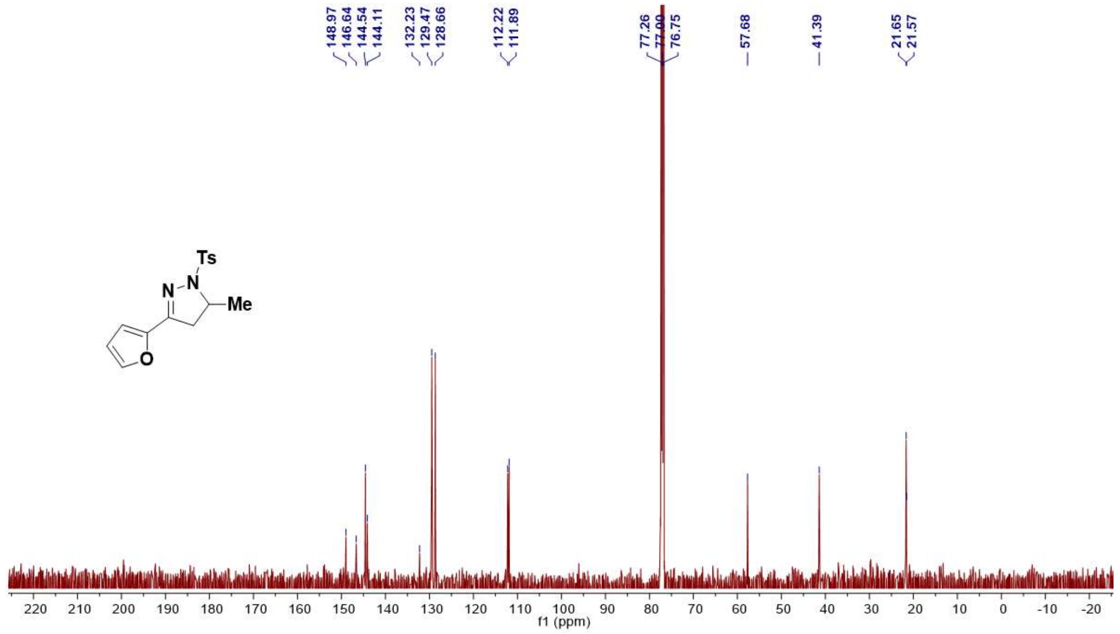

^{1}H NMR (600 MHz, $CDCl_3$) spectrum of product 2q

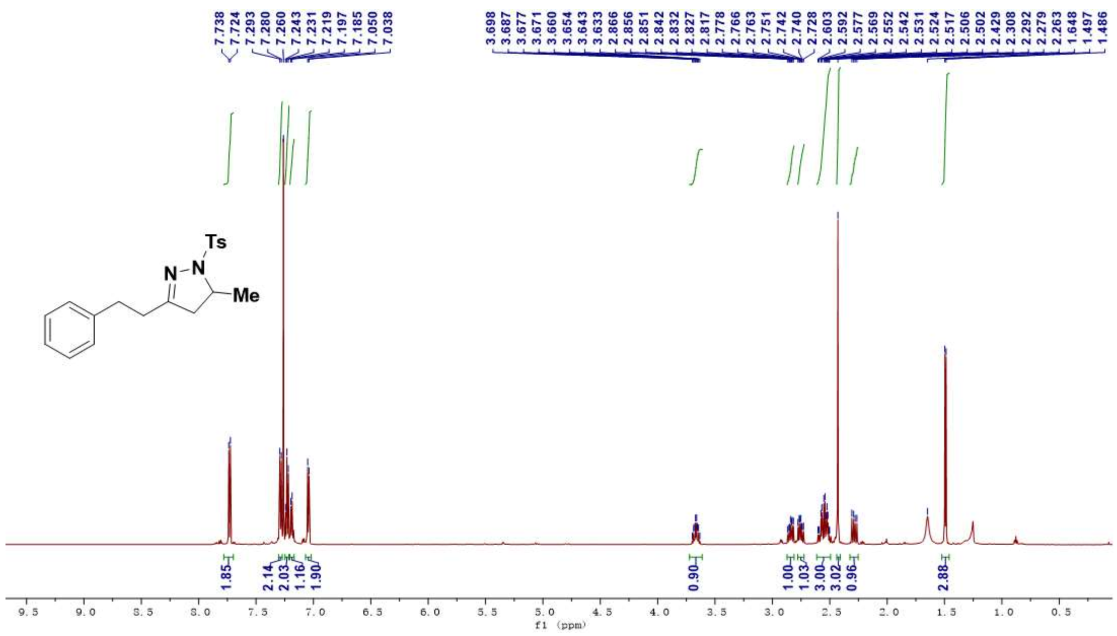

^{13}C NMR (150 MHz, $CDCl_3$) spectrum of product 2q

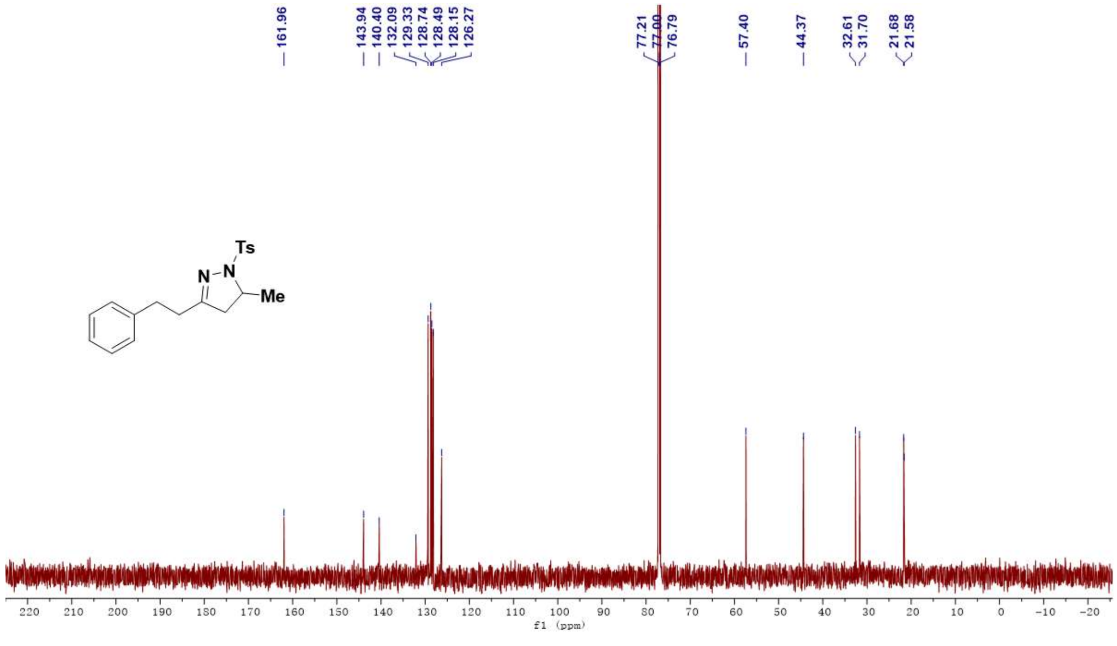

^{1}H NMR (600 MHz, $CDCl_3$) spectrum of product 2r

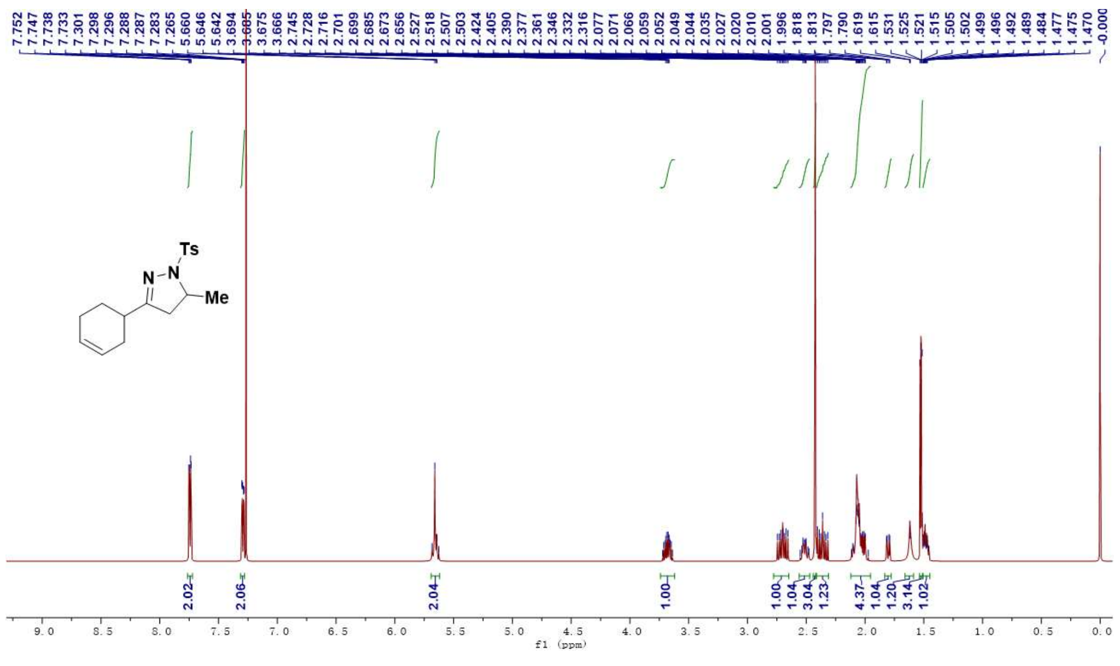

^{13}C NMR (150 MHz, $CDCl_3$) spectrum of product 2r

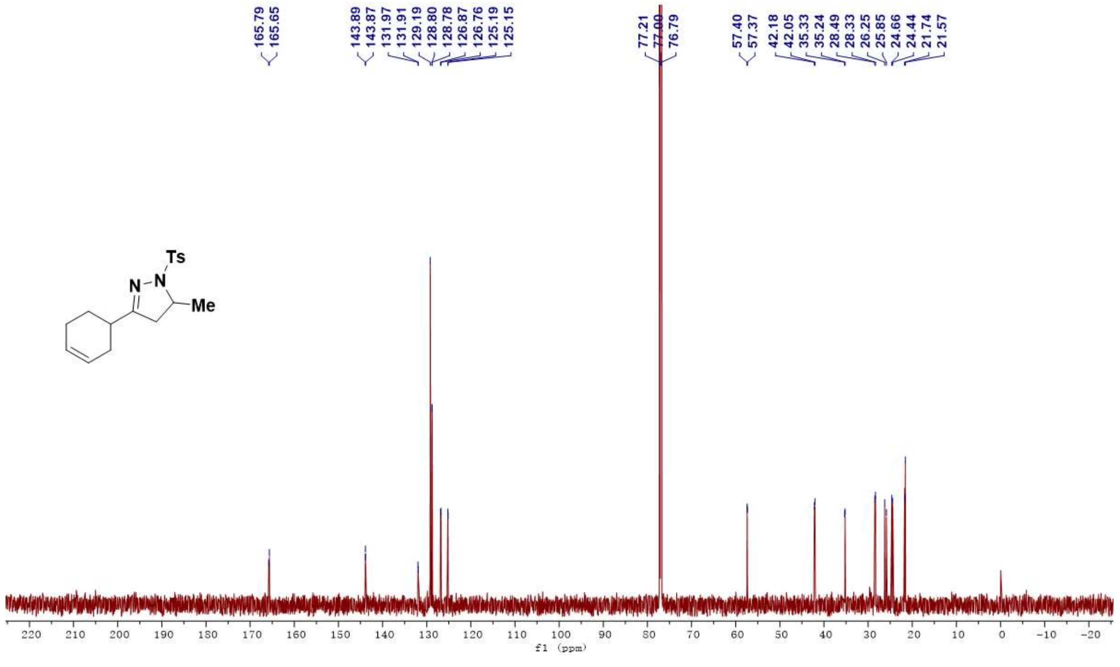

^{1}H NMR (600 MHz, $CDCl_3$) spectrum of product 2s

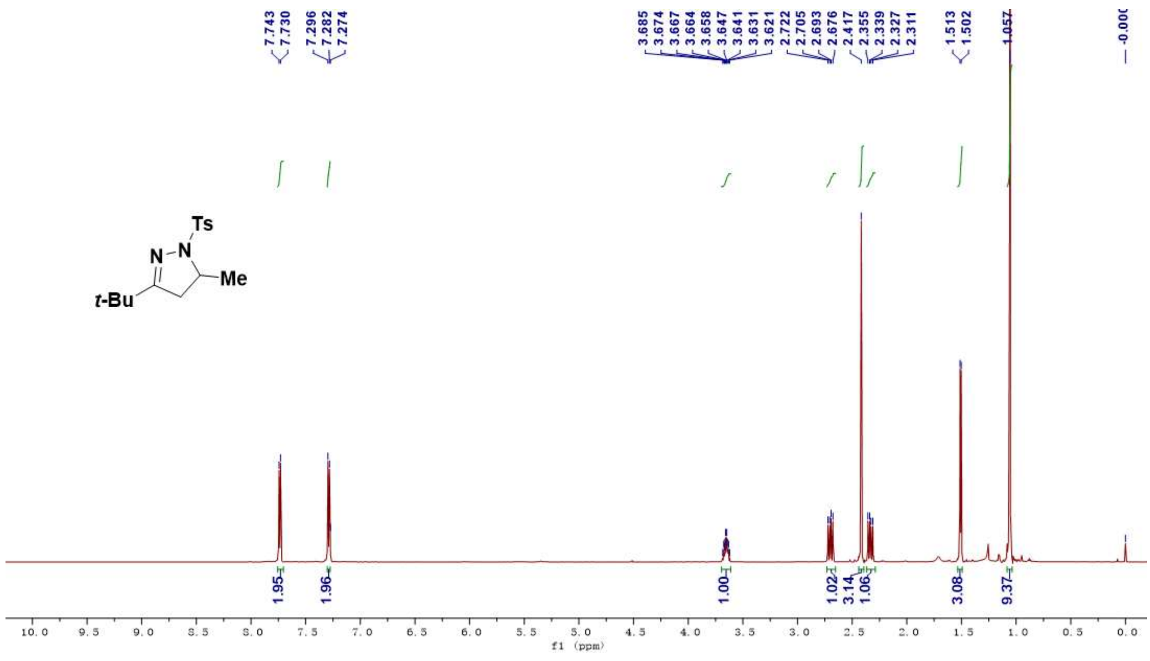

^{13}C NMR (150 MHz, $CDCl_3$) spectrum of product 2s

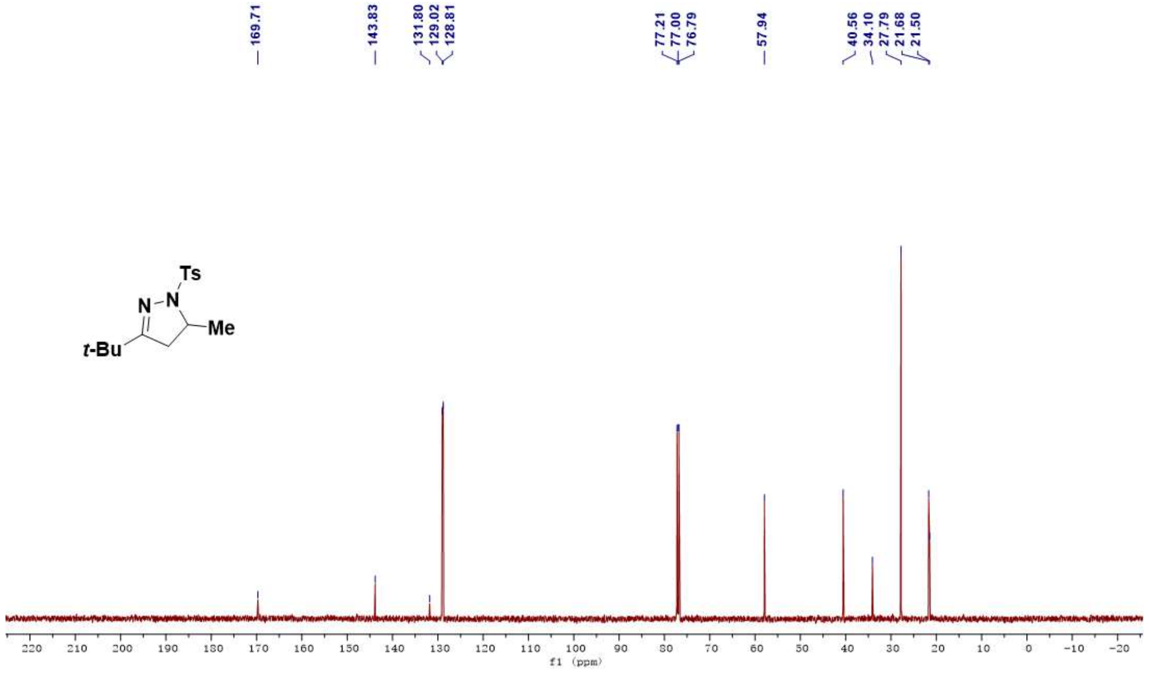

^{1}H NMR (600 MHz, CDCl3) spectrum of product 2t

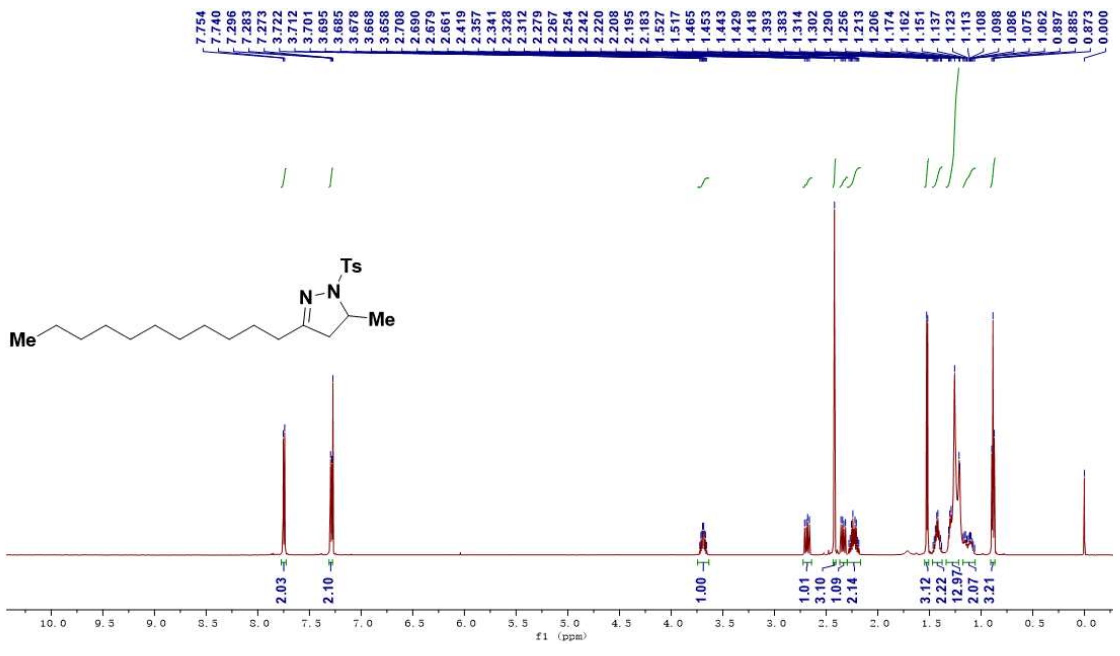

^{13}C NMR (150 MHz, CDCl3) spectrum of product 2t

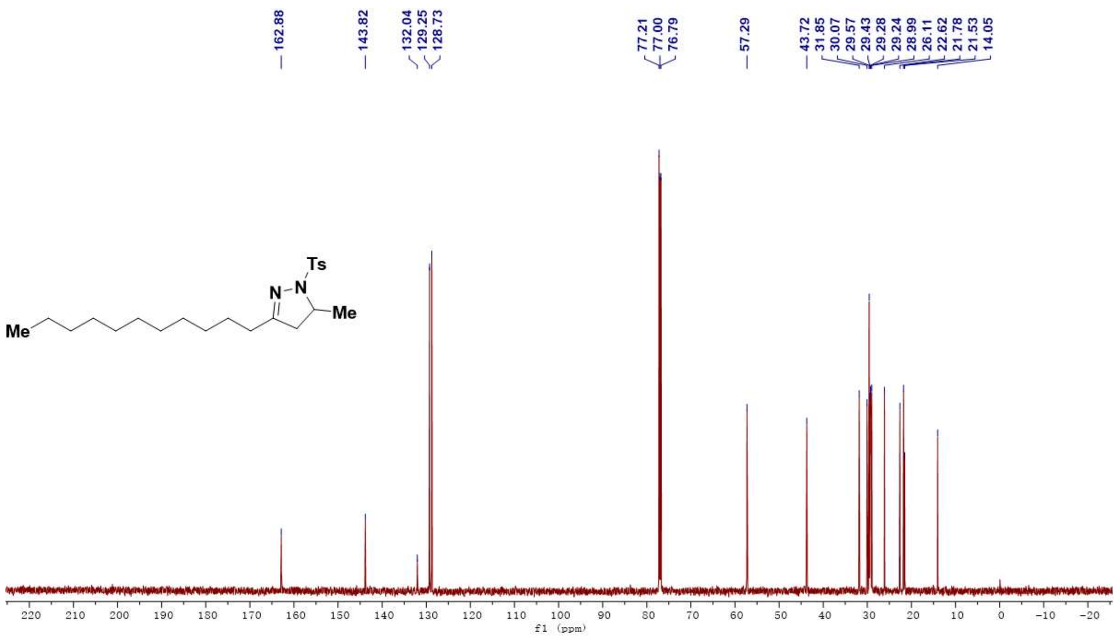

^{1}H NMR (600 MHz, CDCl3) spectrum of product 2u

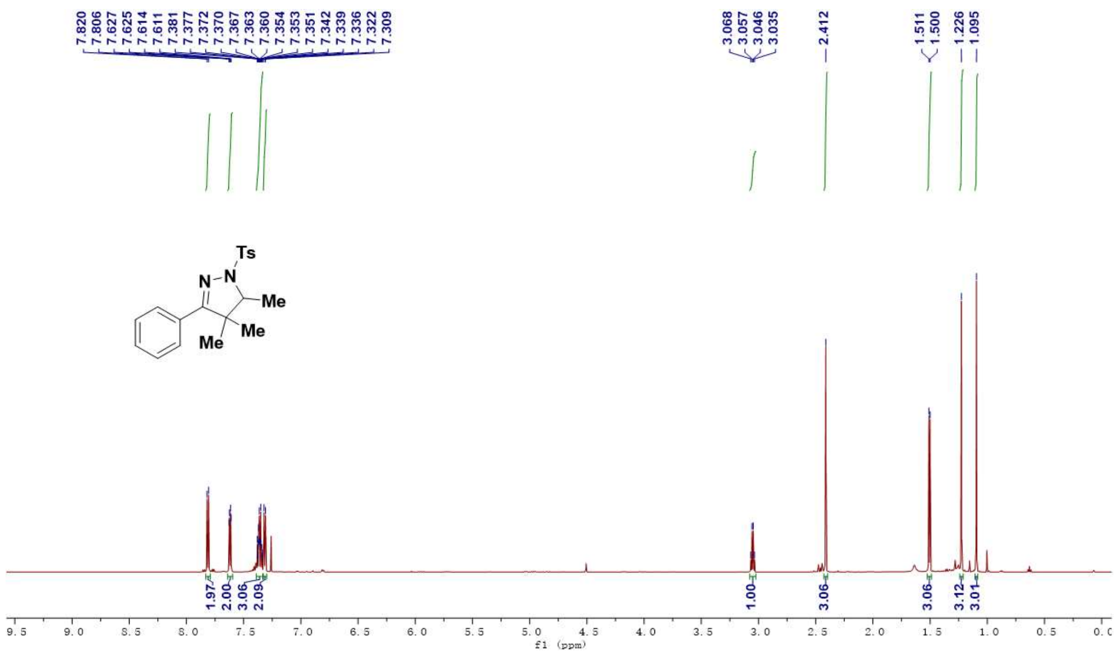

^{13}C NMR (150 MHz, CDCl3) spectrum of product 2u

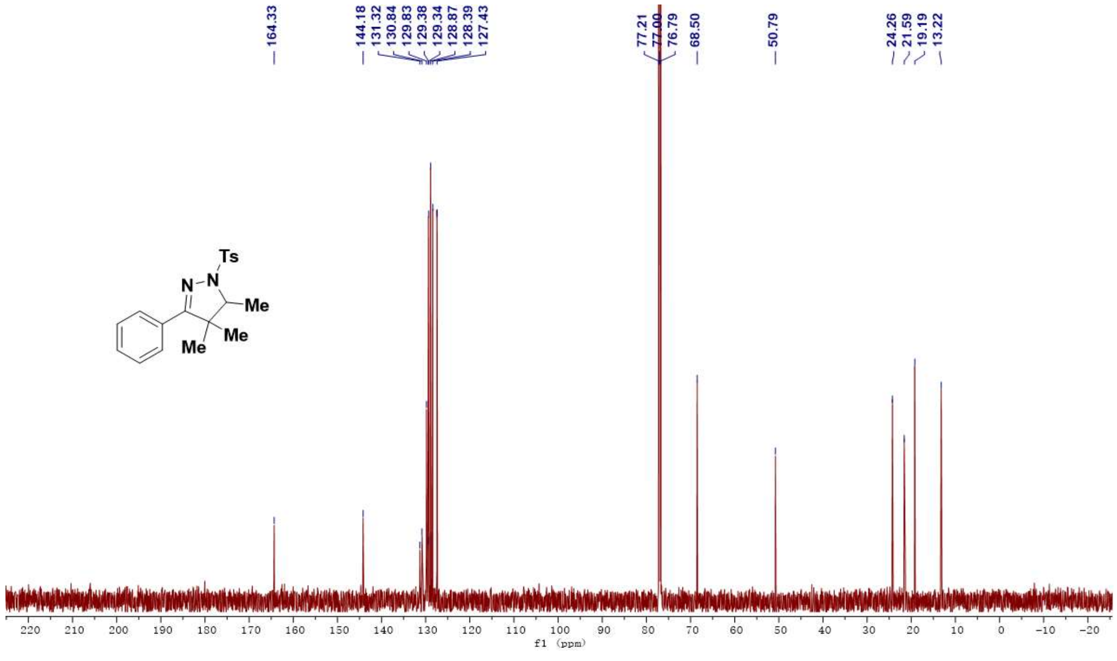

^{1}H NMR (600 MHz, $CDCl_3$) spectrum of product 2v

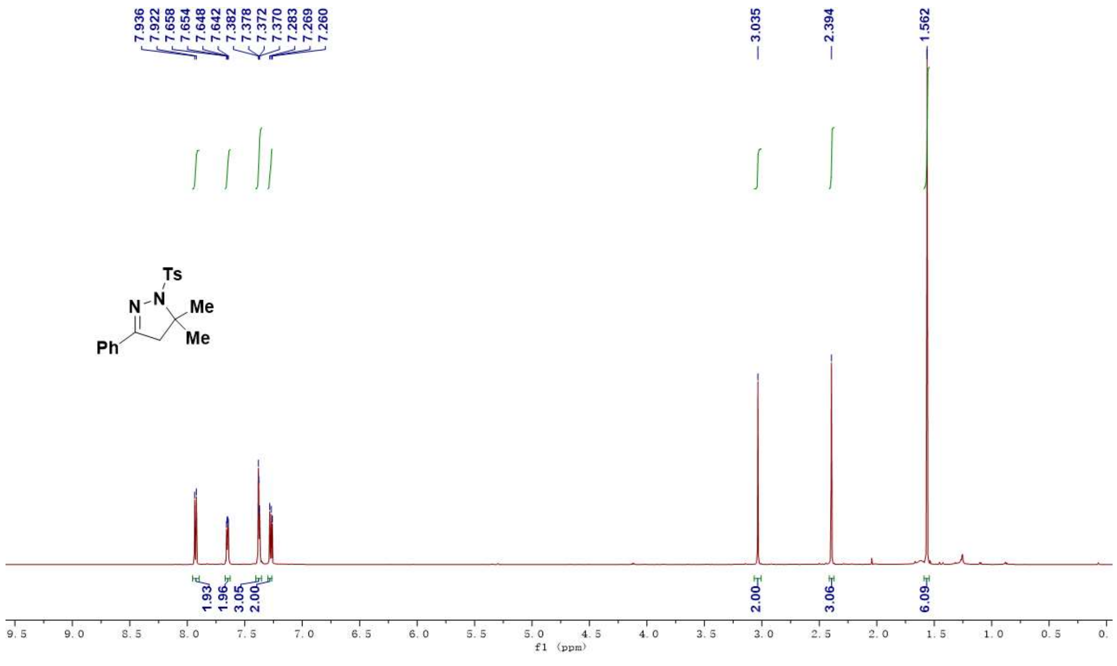

^{13}C NMR (150 MHz, $CDCl_3$) spectrum of product 2v

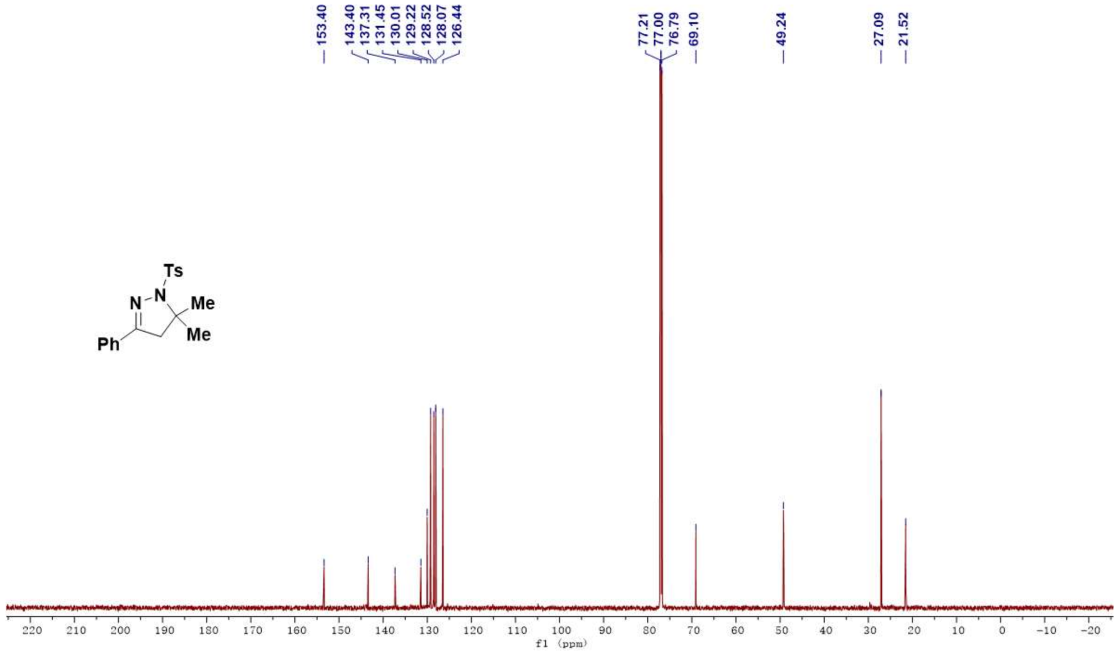

^{1}H NMR (600 MHz, $CDCl_3$) spectrum of product 2w

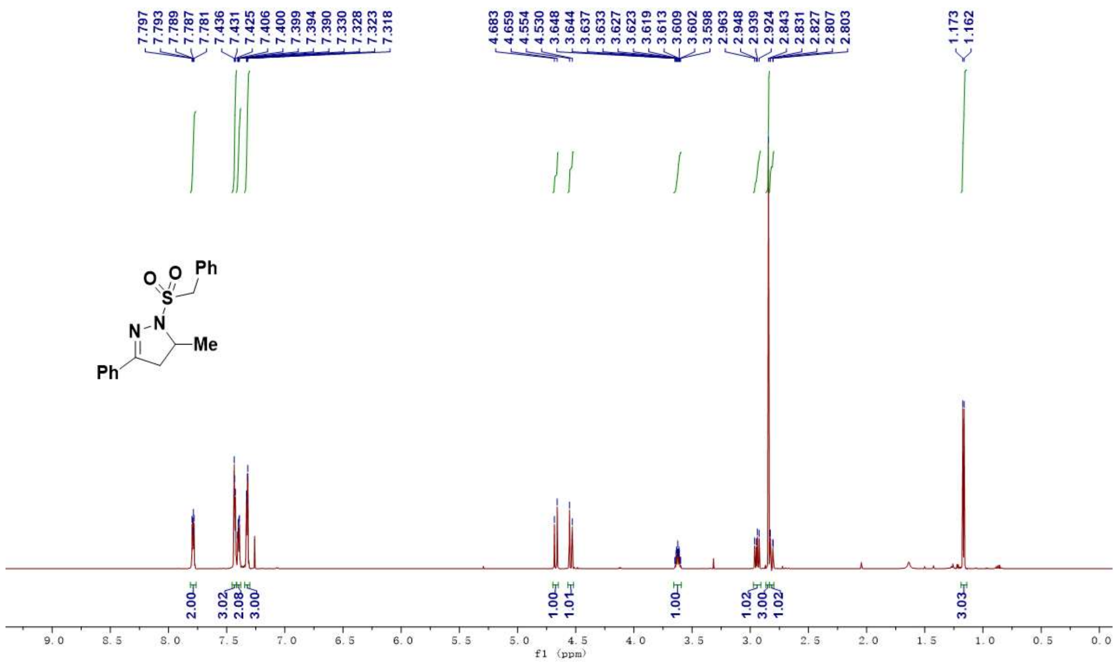

^{13}C NMR (150 MHz, $CDCl_3$) spectrum of product 2w

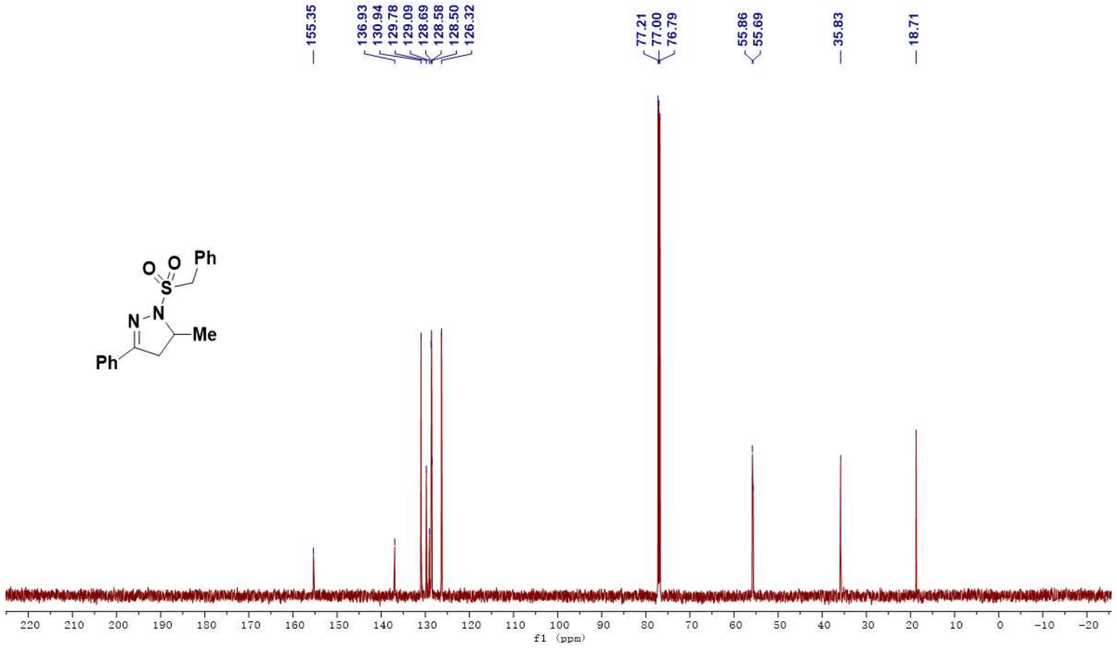

^{1}H NMR (600 MHz, $CDCl_3$) spectrum of product 2aa

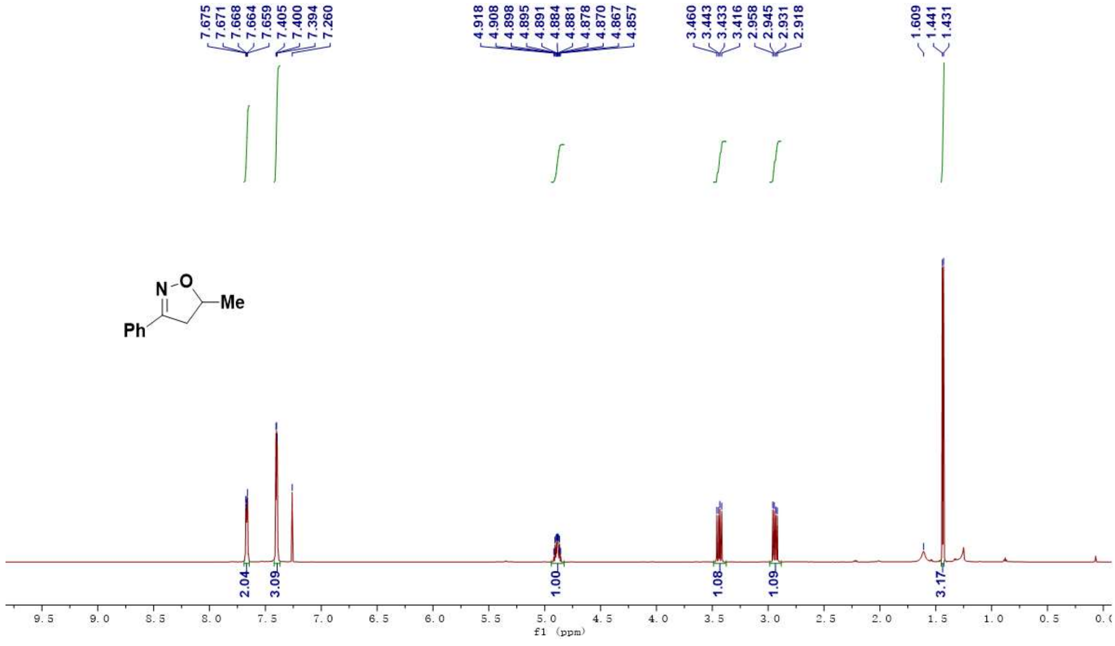

^{13}C NMR (150 MHz, $CDCl_3$) spectrum of product 2aa

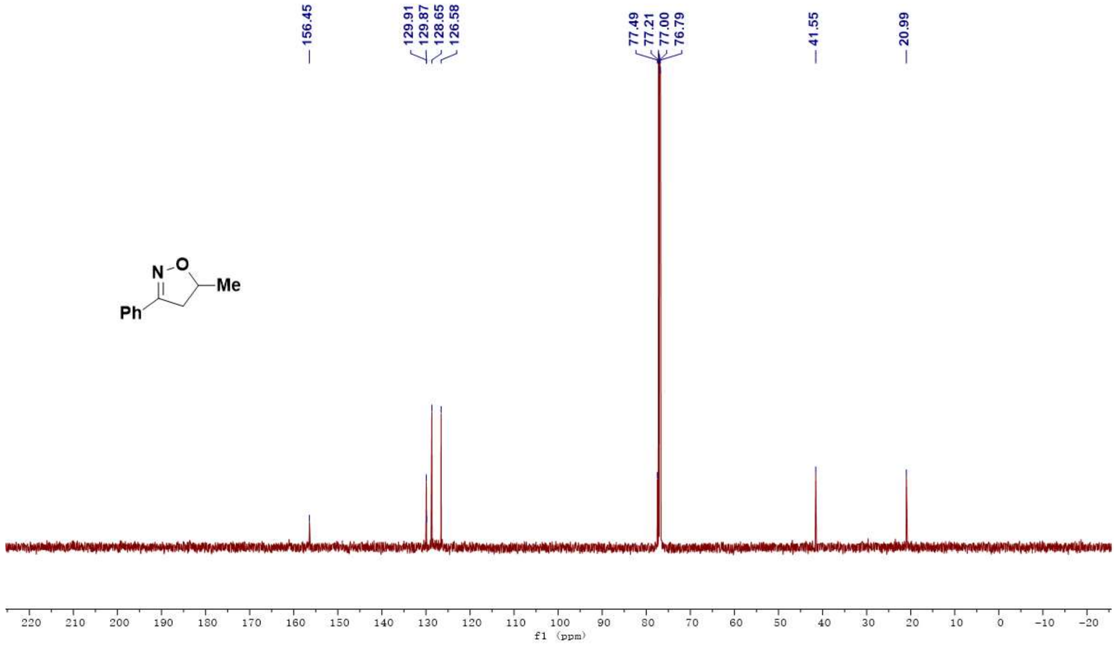

^{1}H NMR (600 MHz, $CDCl_3$) spectrum of product 3a

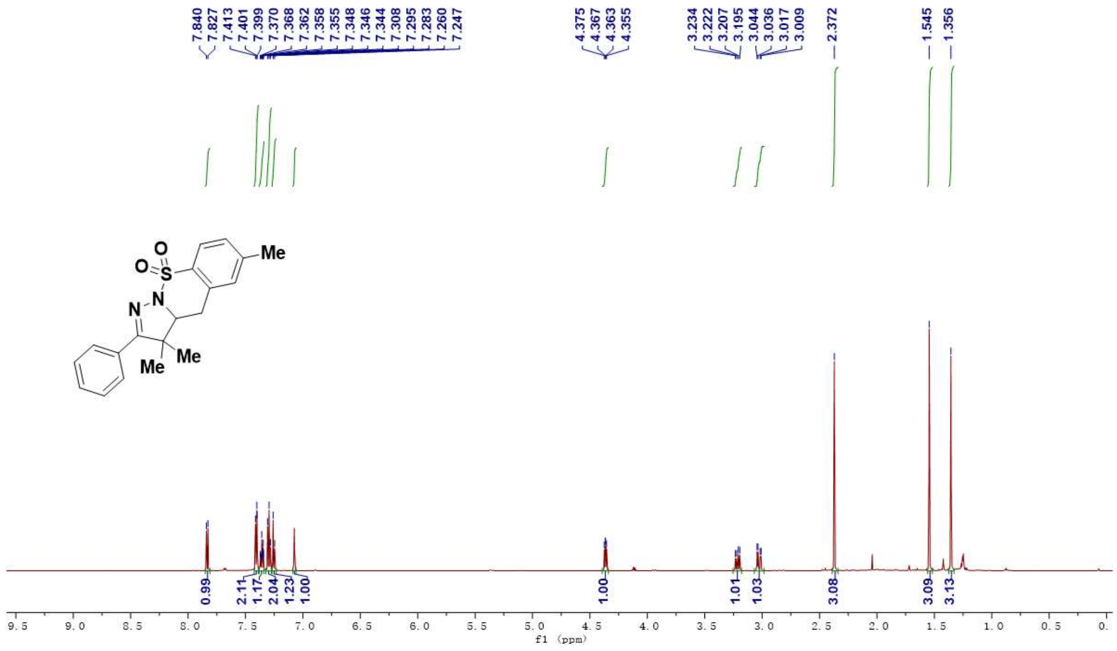

^{13}C NMR (150 MHz, $CDCl_3$) spectrum of product 3a

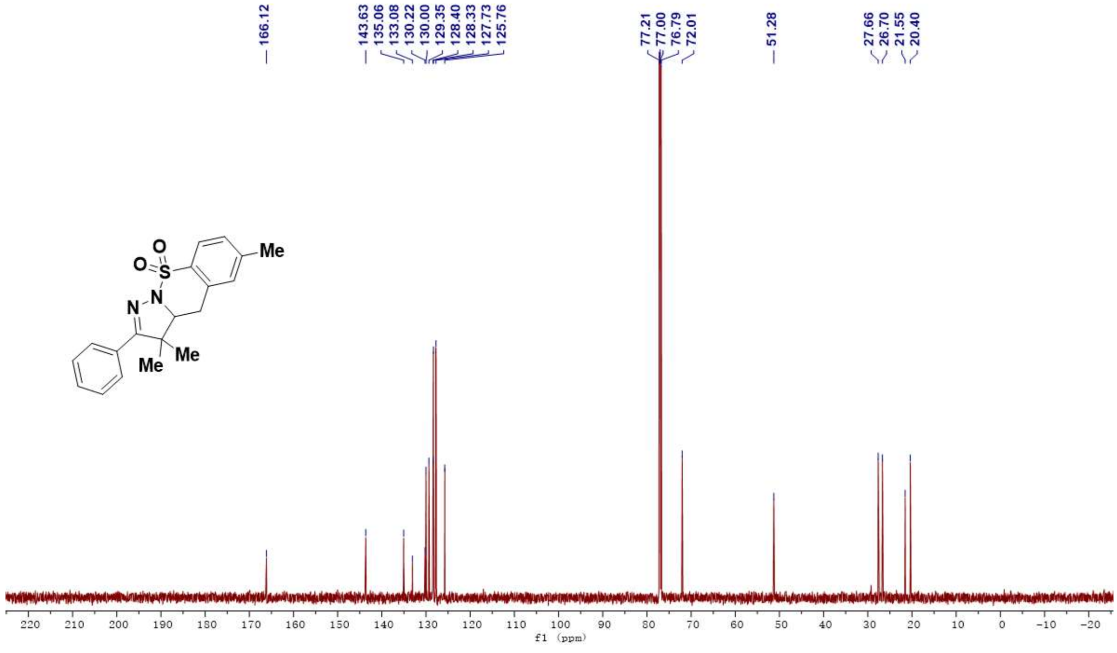

^{1}H NMR (600 MHz, CDCl$_3$) spectrum of product 3b

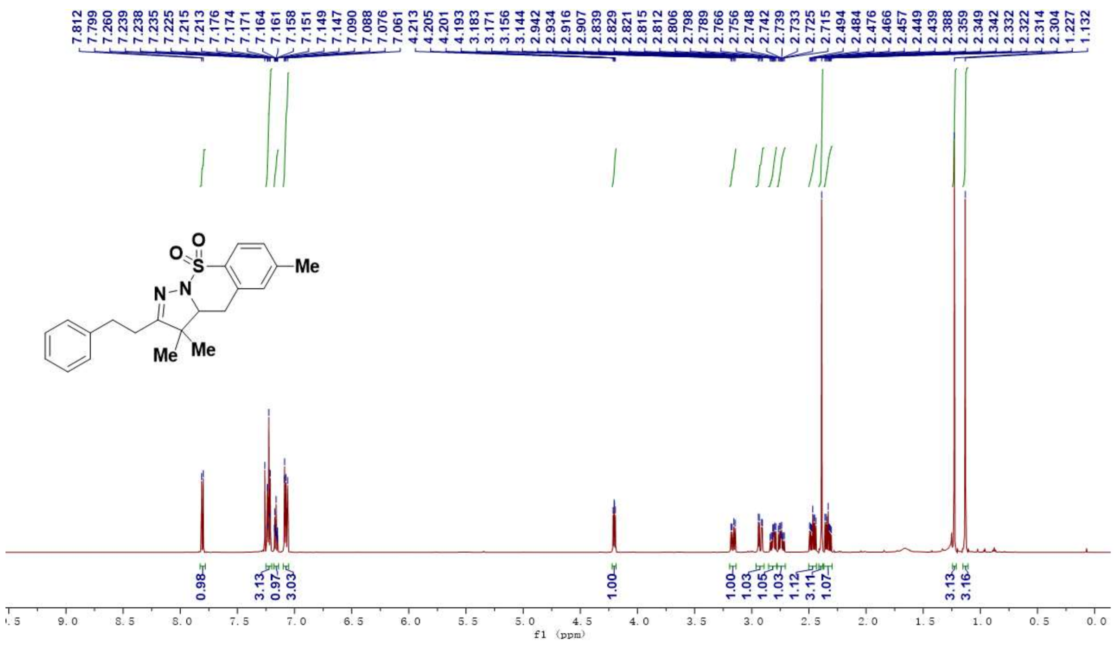

^{13}C NMR (150 MHz, CDCl$_3$) spectrum of product 3b

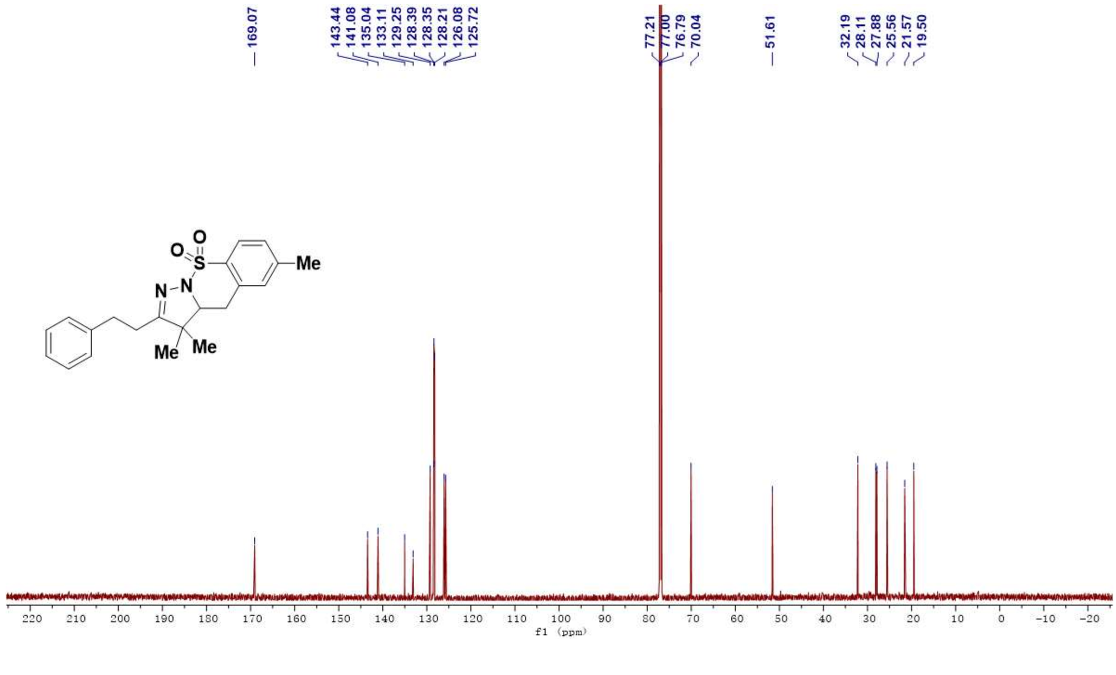

^{1}H NMR (600 MHz, $CDCl_3$) spectrum of product 5a

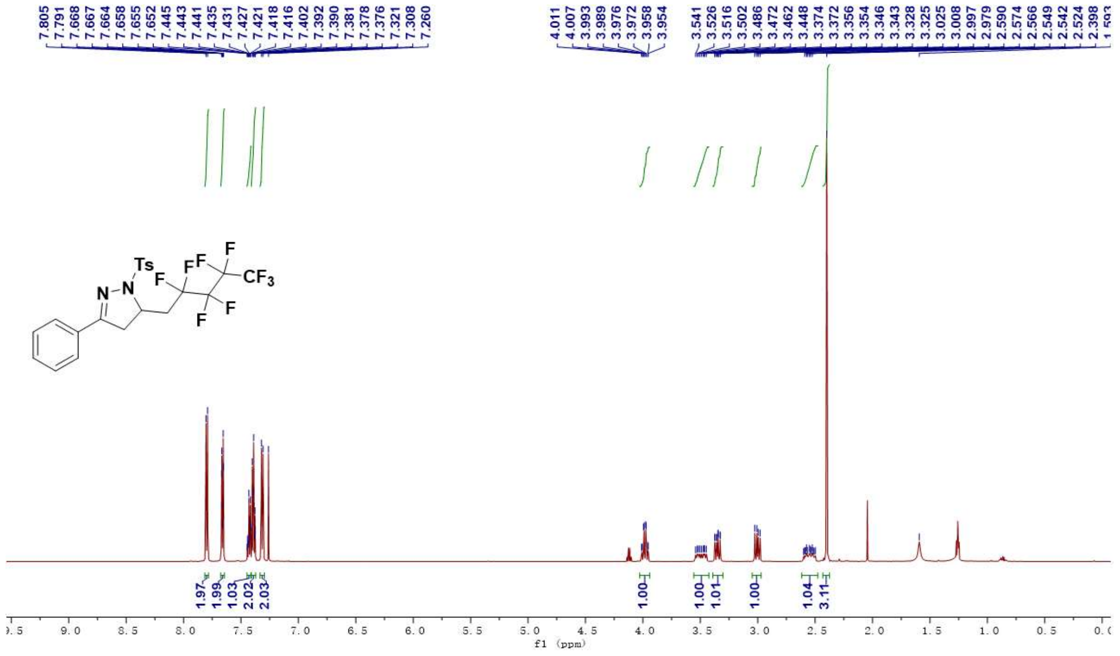

^{13}C NMR (150 MHz, $CDCl_3$) spectrum of product 5a

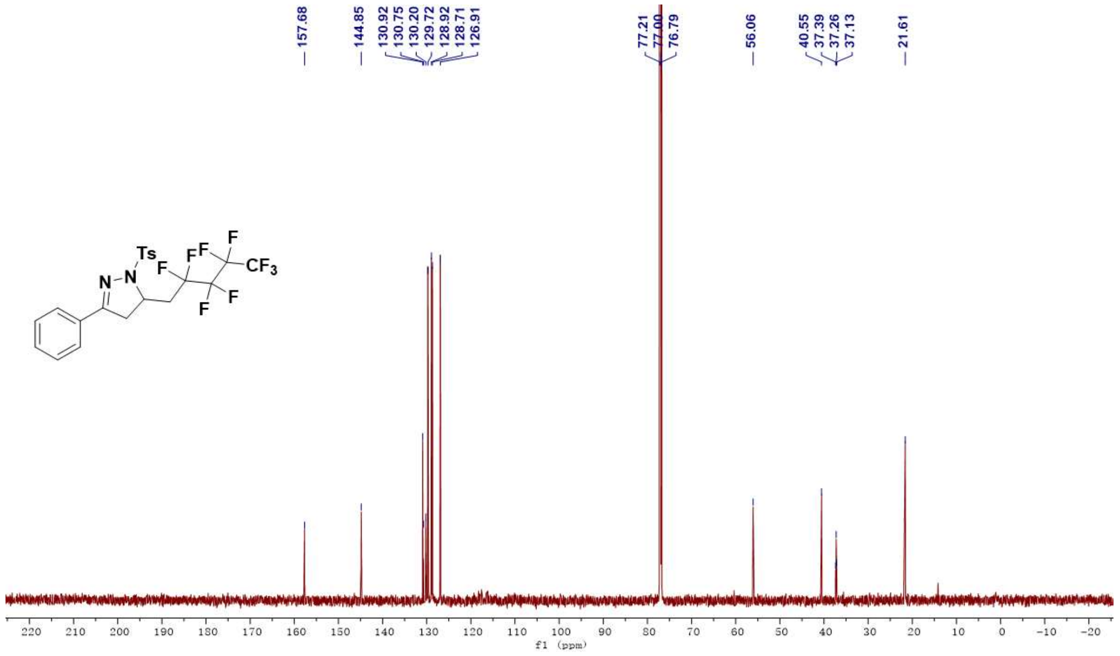

^{19}F NMR (564 MHz, $CDCl_3$) spectrum of product 5a

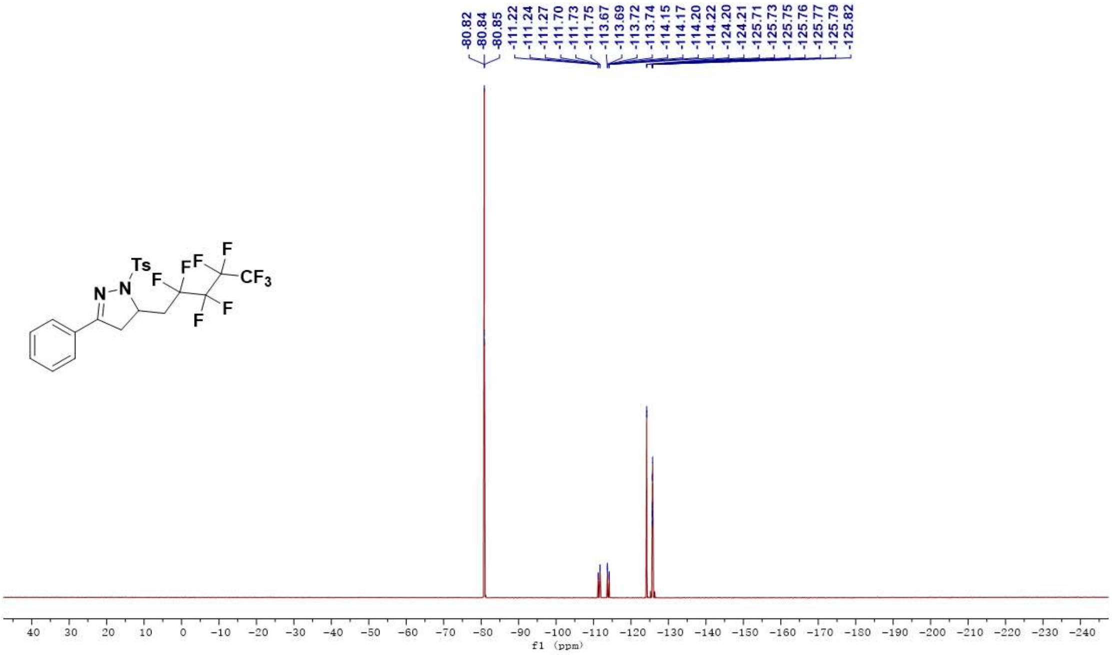

^{1}H NMR (600 MHz, CDCl₃) spectrum of product 5b

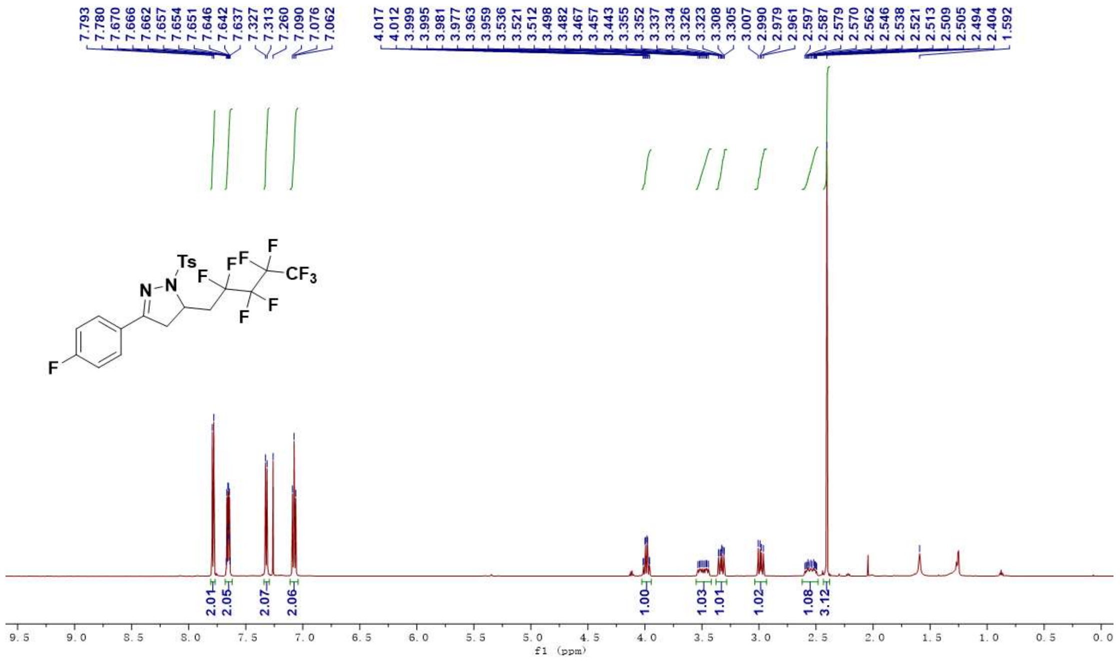

^{13}C NMR (150 MHz, CDCl₃) spectrum of product 5b

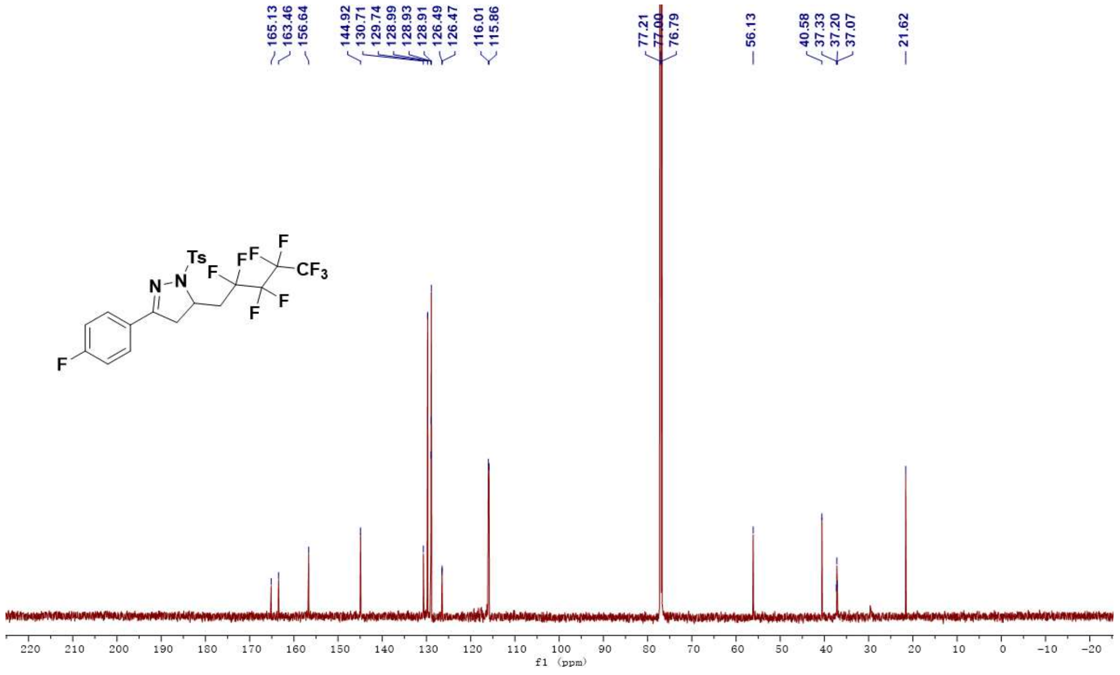

^{19}F NMR (564 MHz, $CDCl_3$) spectrum of product 5b

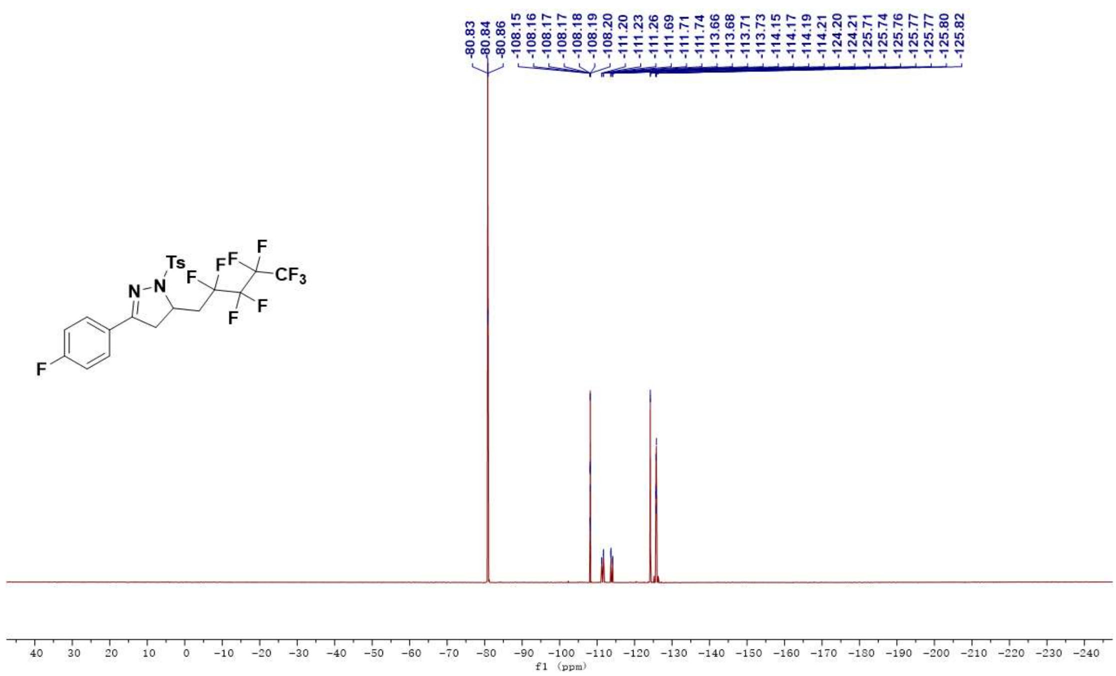

^{1}H NMR (600 MHz, $CDCl_3$) spectrum of product 5c

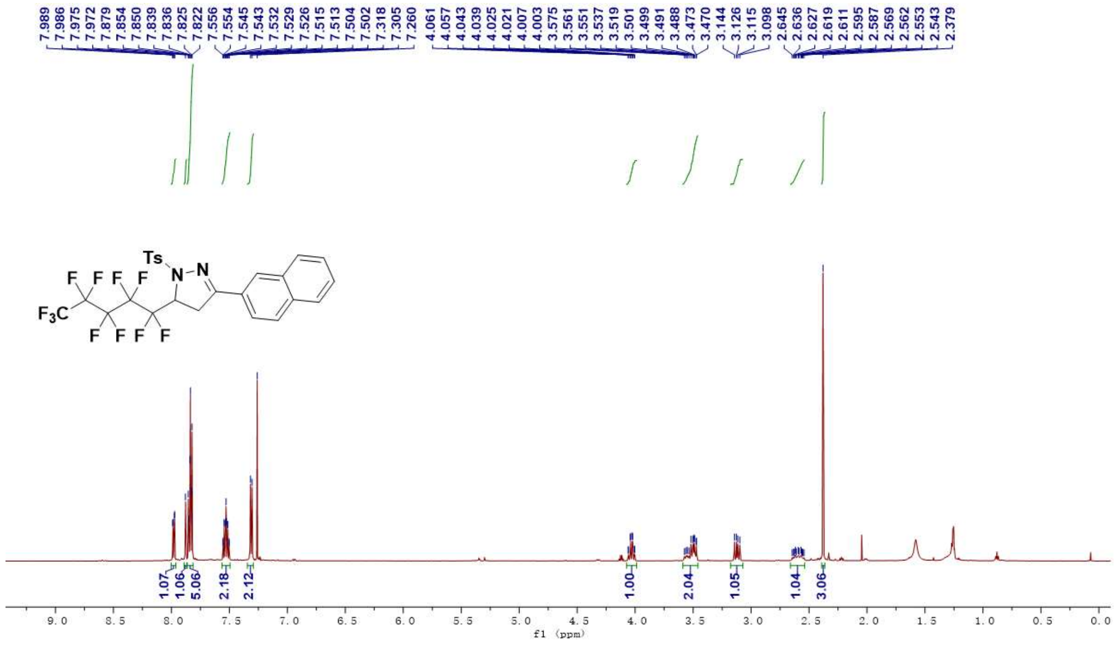

^{13}C NMR (150 MHz, $CDCl_3$) spectrum of product 5c

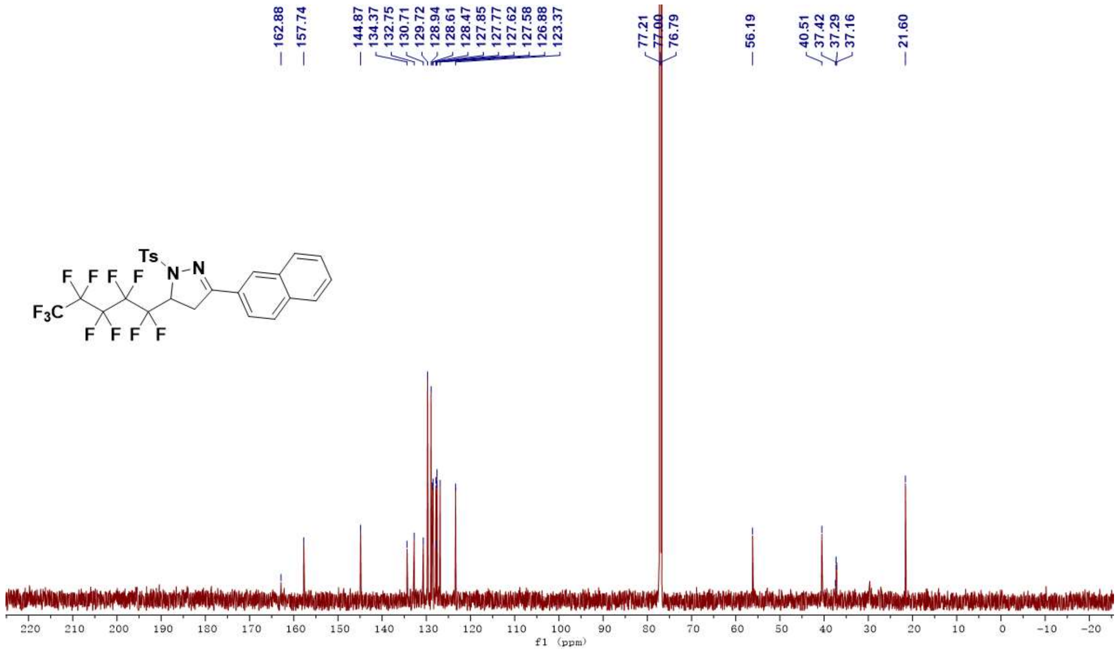

^{19}F NMR (564 MHz, CDCl$_3$) spectrum of product 5c

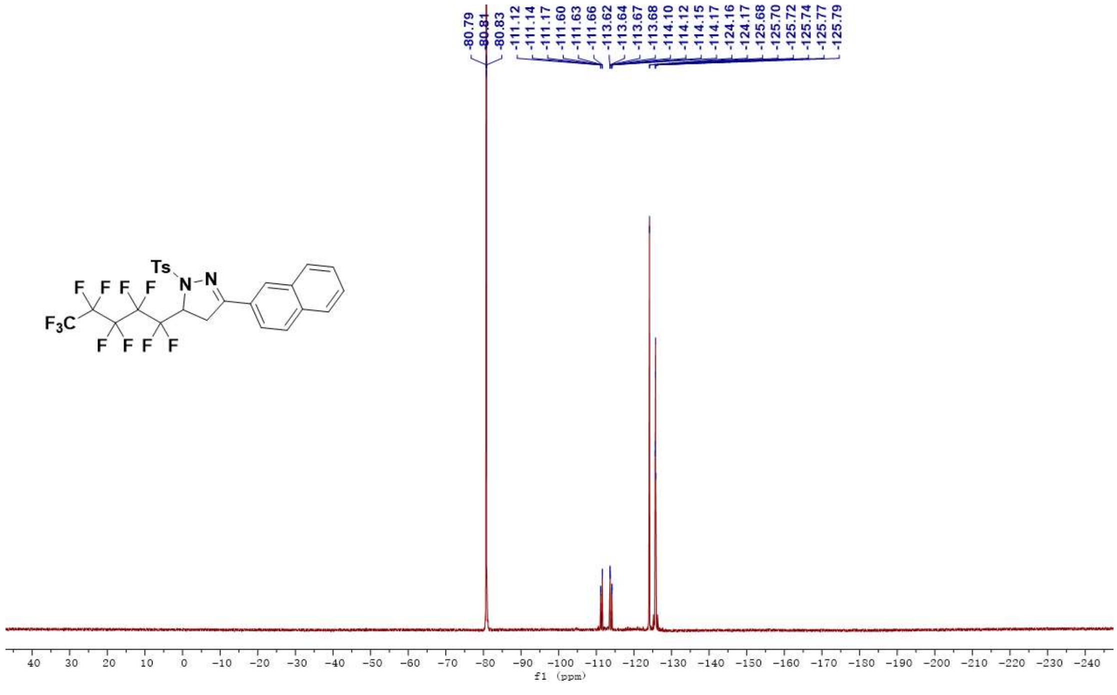

^{1}H NMR (600 MHz, $CDCl_3$) spectrum of product 7a

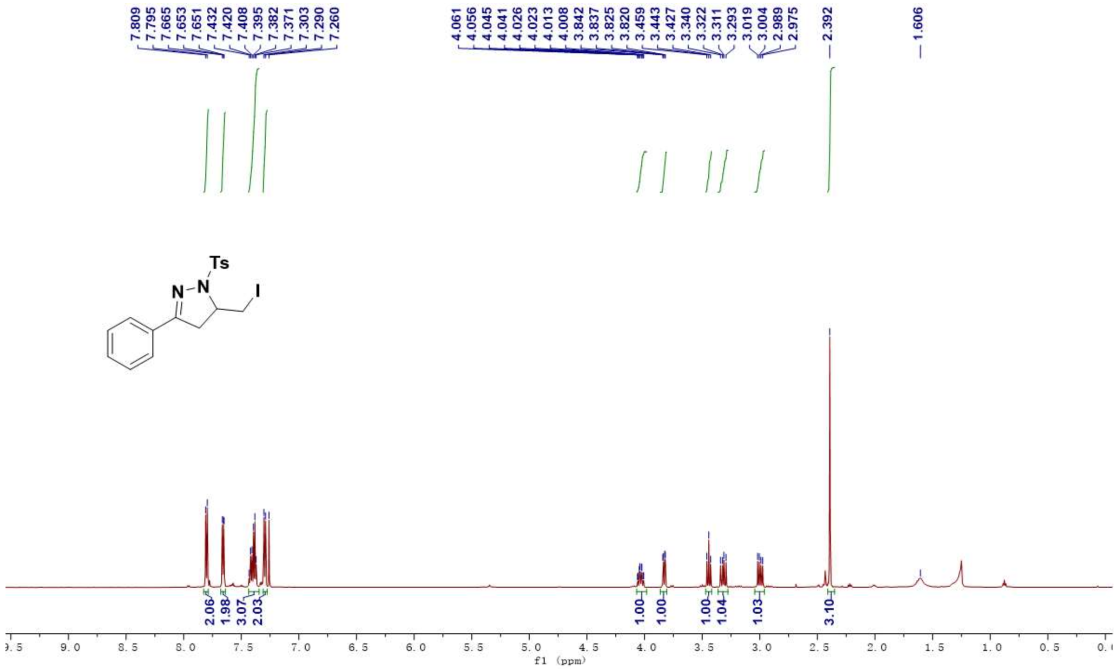

^{13}C NMR (150 MHz, $CDCl_3$) spectrum of product 7a

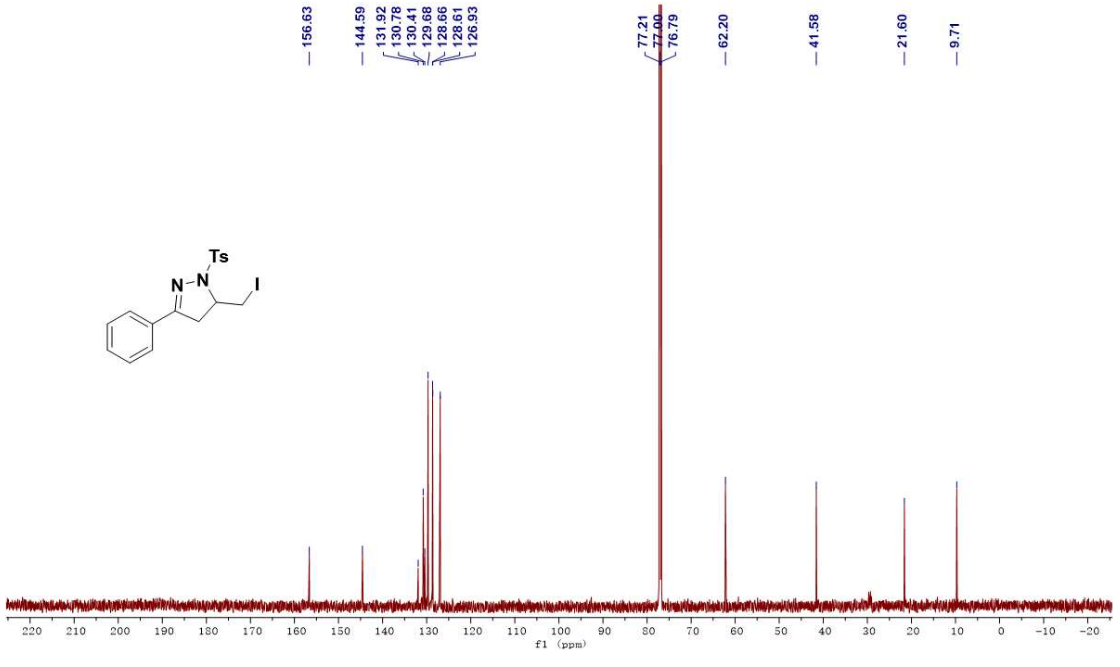

^{1}H NMR (600 MHz, $CDCl_3$) spectrum of product 7b

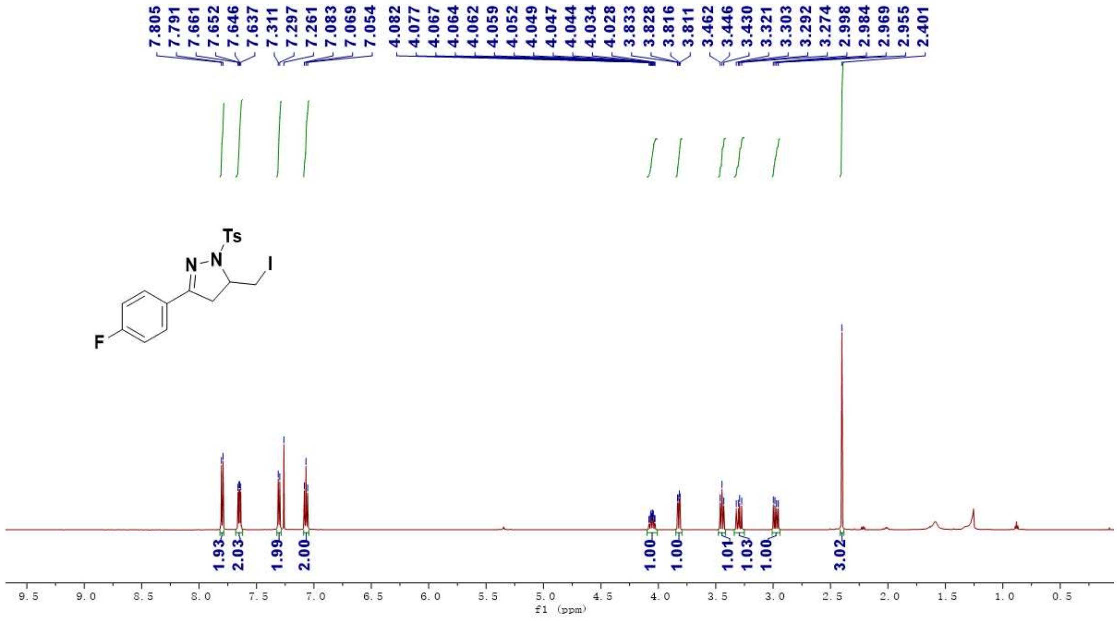

^{13}C NMR (150 MHz, $CDCl_3$) spectrum of product 7b

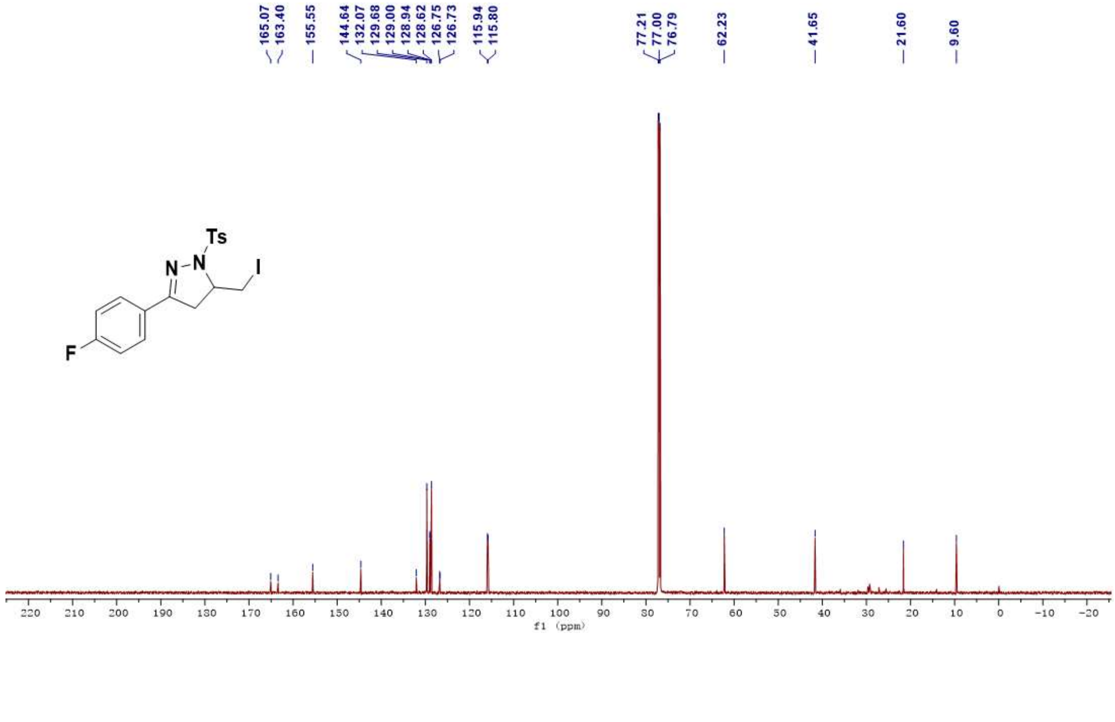

^{1}H NMR (600 MHz, CDCl$_3$) spectrum of product 7c

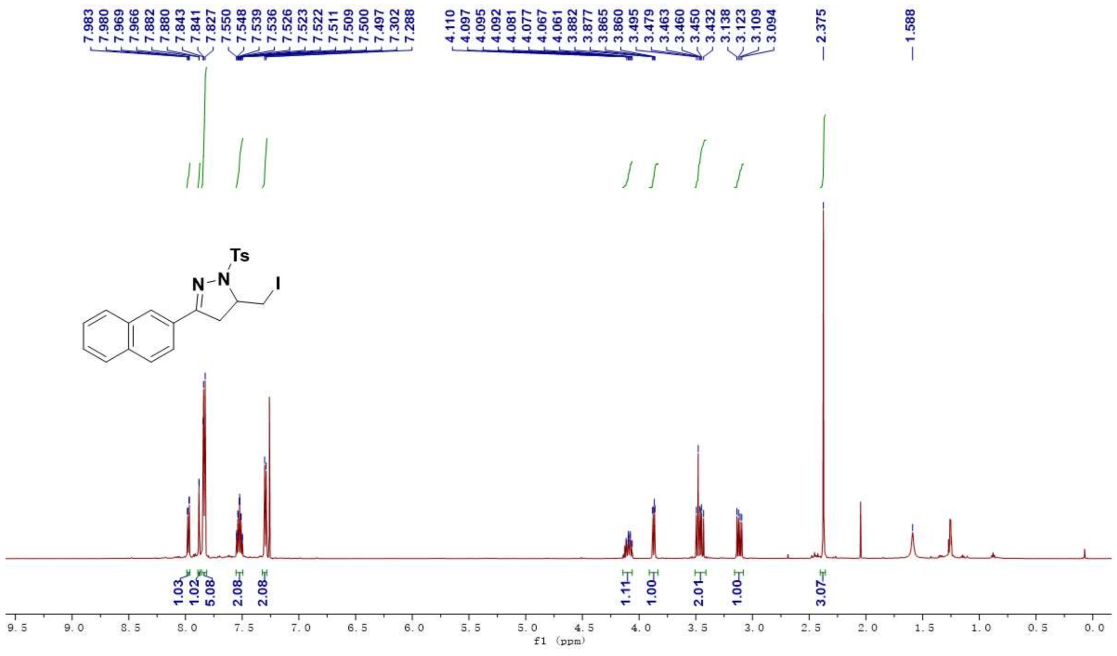

^{13}C NMR (150 MHz, CDCl$_3$) spectrum of product 7c

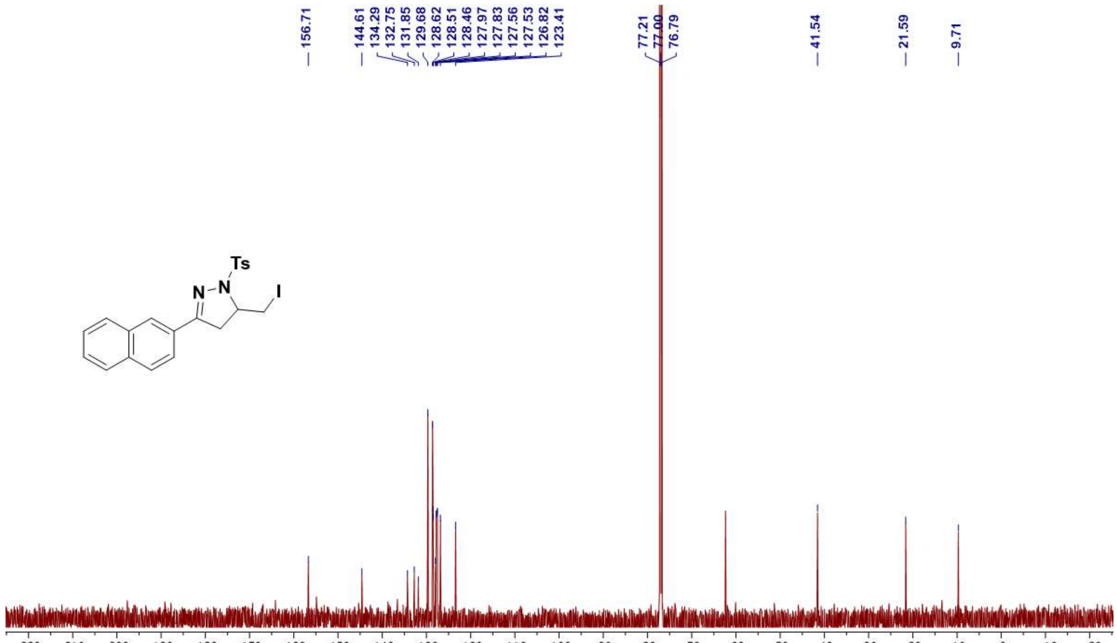

^{1}H NMR (600 MHz, $CDCl_3$) spectrum of product 8

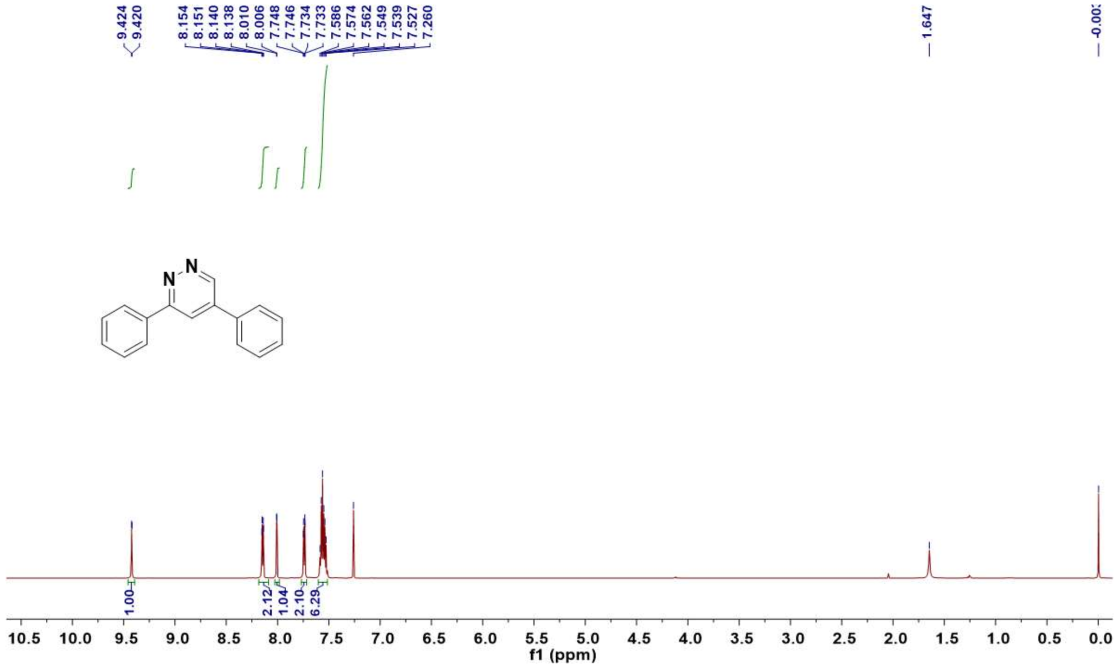

^{13}C NMR (150 MHz, $CDCl_3$) spectrum of product 8

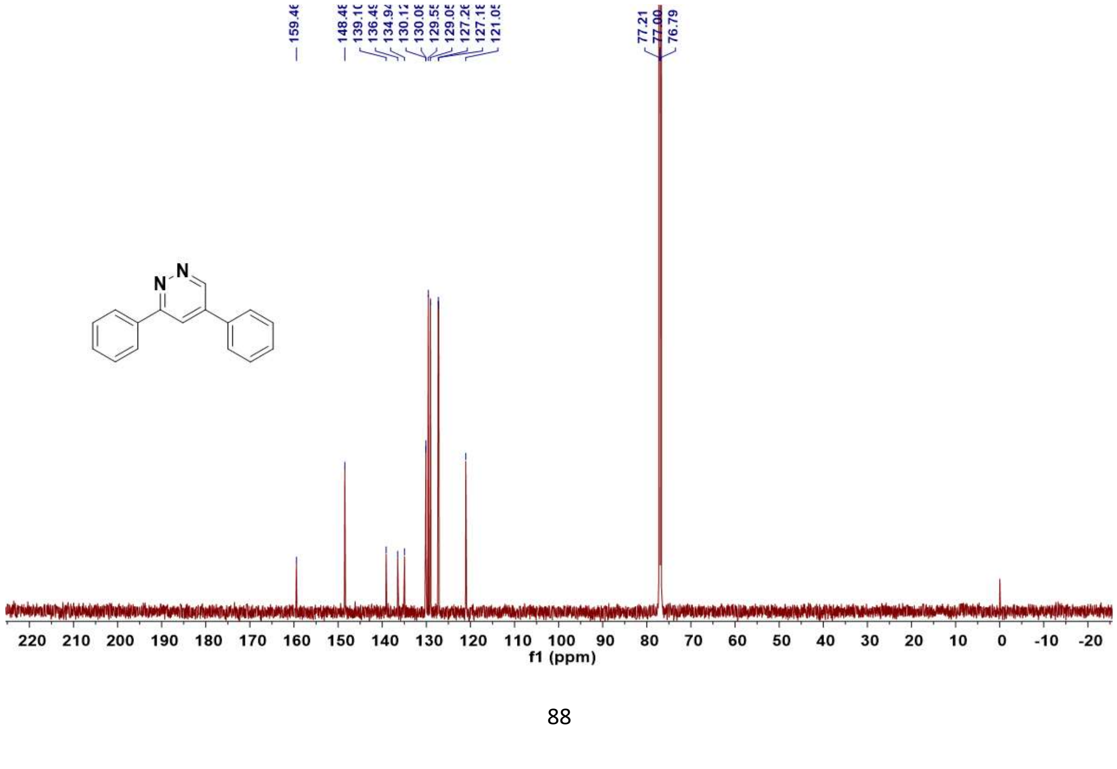

^{1}H NMR (600 MHz, $CDCl_3$) spectrum of product 9

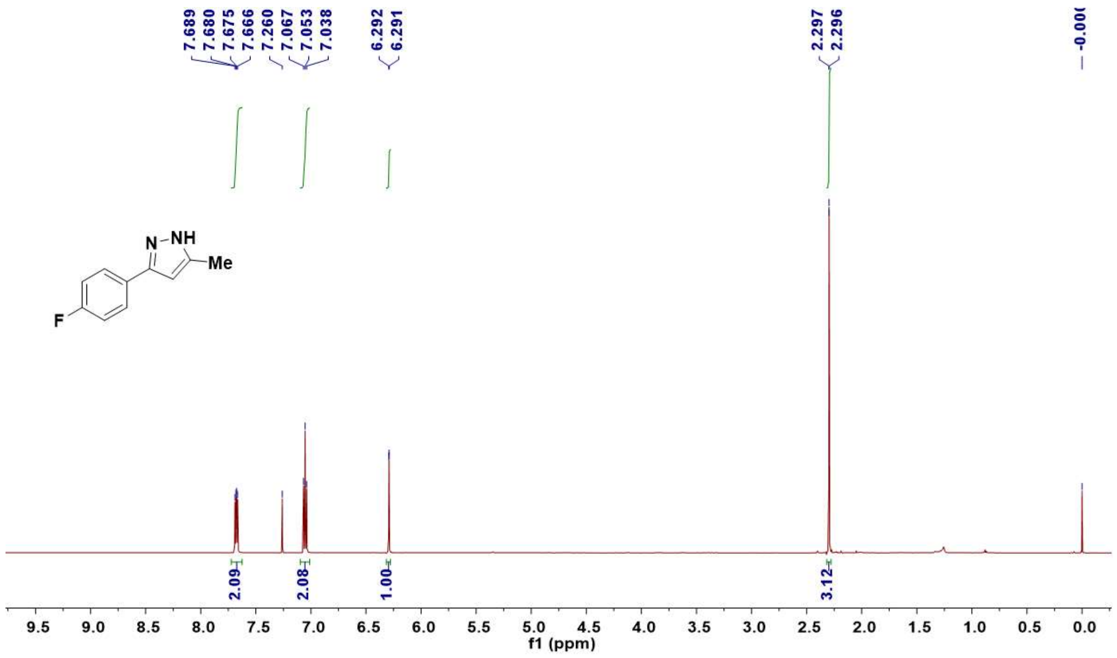

^{13}C NMR (150 MHz, $CDCl_3$) spectrum of product 9

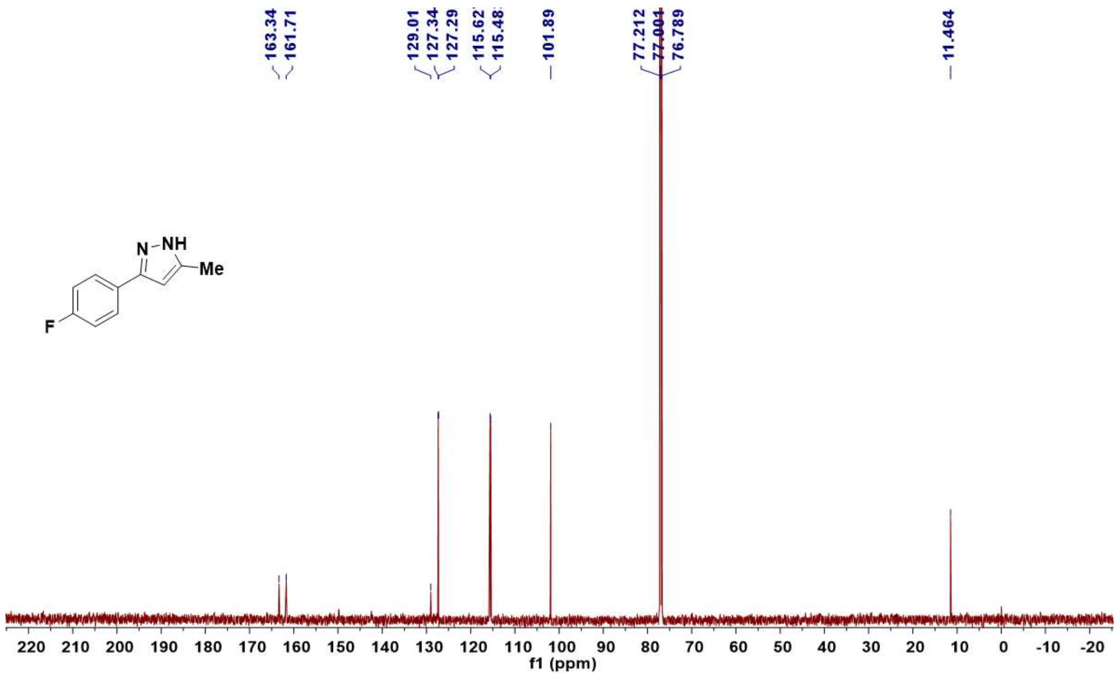

^{1}H NMR (600 MHz, CDCl₃) spectrum of product 10

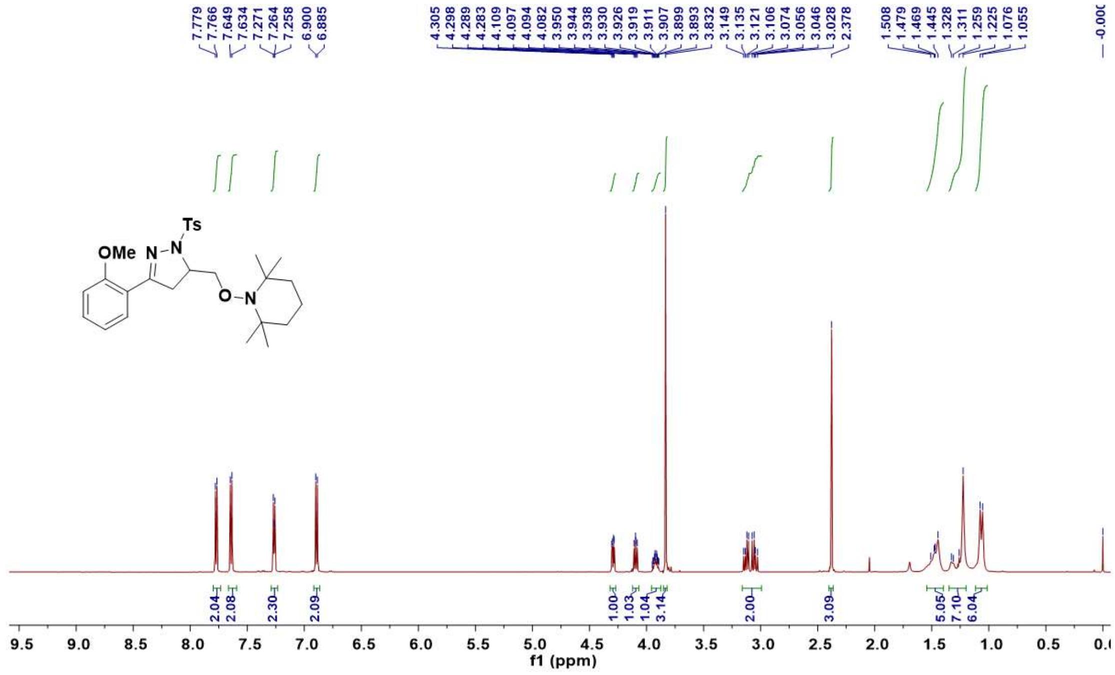

^{13}C NMR (150 MHz, CDCl₃) spectrum of product 10

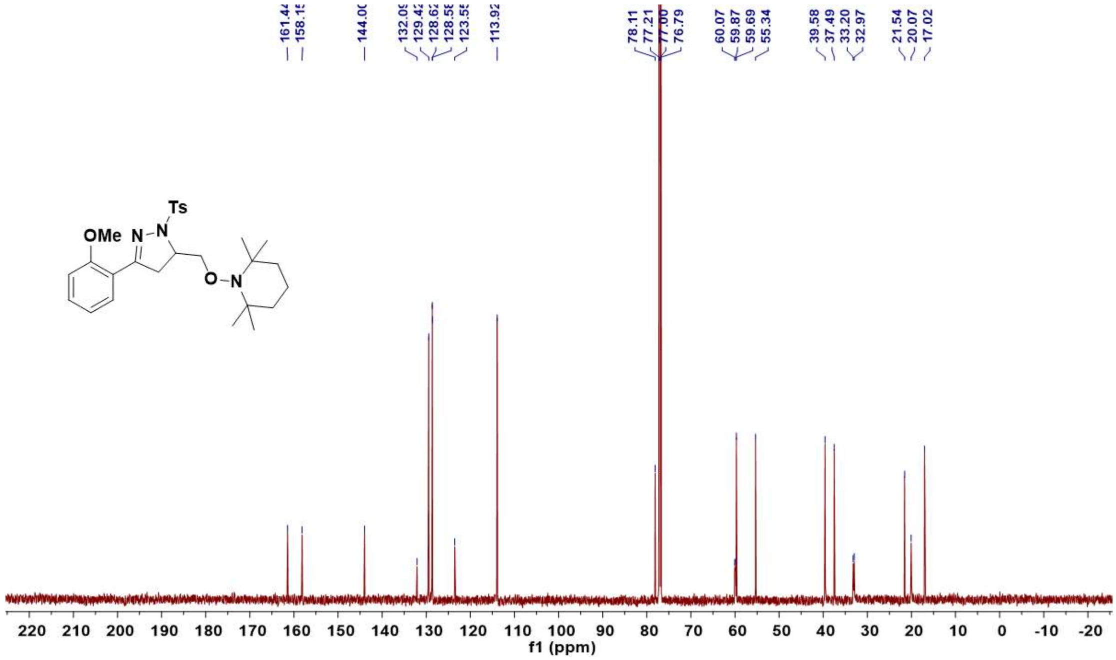

Supplement: Supplementary file 1 — Supplementary Information [file 41467_2022_32623_MOESM1_ESM.pdf]
